# Catalytic asymmetric synthesis of carbocyclic C-nucleosides

**DOI:** 10.1038/s42004-022-00773-6

**Published:** 2022-11-19

**Authors:** Sourabh Mishra, Florian C. T. Modicom, Conor L. Dean, Stephen P. Fletcher

**Affiliations:** Department of Chemistry, Chemistry Research Laboratory, University of Oxford, Oxford, OX1 3TA UK

**Keywords:** Synthetic chemistry methodology, Asymmetric catalysis, Asymmetric synthesis

## Abstract

Access to carbocyclic C-nucleosides (CC-Ns) is currently restricted. The few methods available to make CC-Ns suffer from long syntheses and poor modularity, hindering the examination of potentially important chemical space. Here we report an approach to CC-Ns which uses an asymmetric Suzuki-Miyaura type reaction as the key C-C bond forming step. After coupling the densely functionalized racemic bicyclic allyl chloride and heterocyclic boronic acids, the trisubstituted cyclopentenyl core is elaborated to RNA analogues via a hydroborylation-homologation-oxidation sequence. We demonstrate that the approach can be used to produce a variety of enantiomerically enriched CC-Ns, including a carbocyclic derivative of Showdomycin.

## Abstract (web summary)

Carbocyclic nucleosides show a broad spectrum of antiviral activity with enhanced flexibility, lipophilicity and metabolic stability, however, synthetic access to carbocyclic C-nucleosides (CC-Ns) remains very challenging. Here, the authors use an asymmetric Suzuki-Miyaura coupling reaction as the key C-C bond forming step to produce a variety of enantiomerically enriched CC-Ns, including antiviral showdomycin.

## Introduction

**Fig. 1:**
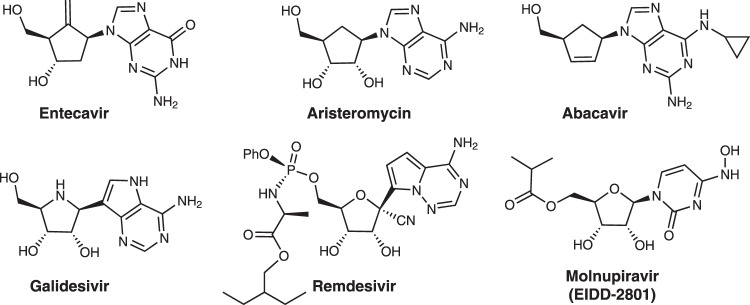
Antiviral nucleosides analogs. Few examples of nucleoside analogs as commercially available antiviral drugs.

Nucleosides and their analogs are widely studied antiviral and anticancer agents^1–3^. Carbocyclic nucleoside analogs show a broad spectrum of antiviral activity and are known to exhibit enhanced flexibility, lipophilicity and metabolic stability^4–6^. For instance, Entecavir is clinically used to treat hepatitis B;^7,8^ Aristeromycin and Abacavir are active against HIV^9,10^. Additionally, nucleoside analog Galidesivir is a potent inhibitor of Ebola and Zika viruses^11,12^. There has been much recent interest in using nucleoside in the treatment of severe acute respiratory syndrome coronavirus 2 (SARS-COV-2); Remdesivir^13,14^ and Molnupiravir^15,16^ demonstrate activity against SARS-COV-2 and have been approved for high-risk patients with severe symptoms (Fig. 1).

**Fig. 2:**
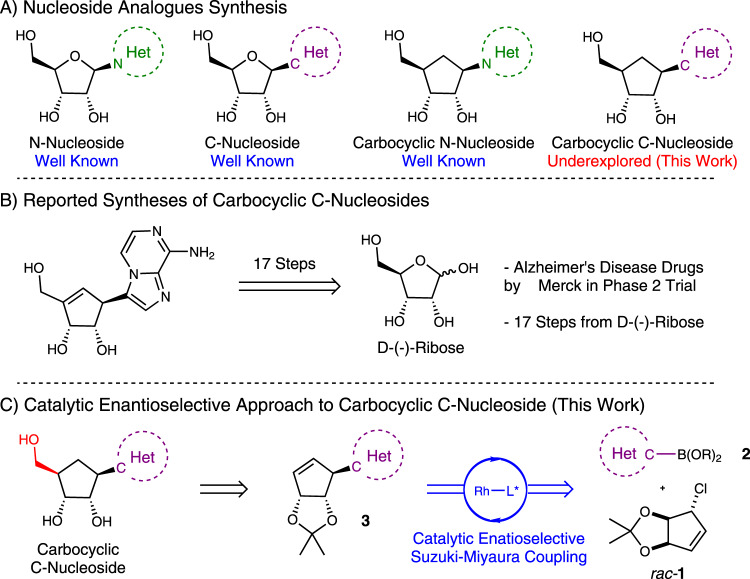
Classification and synthesis of carbocyclic C-nucleosides. **A** Nucleoside analog synthesis. **B** Reported syntheses of carbocyclic C-Nucleosides. **C** Catalytic enantioselective approach to carbocyclic C-Nucleosides (this work).

The synthesis and bioactivities of N-nucleosides, C-nucleosides, and carbocyclic N-nucleosides (Fig. 2A) are extensively studied^17–21^. Nucleophilic addition to carbohydrate-derived oxocarbeniums is widely used to access N- and C-nucleosides^20^. Strategies for the synthesis of carbocyclic nucleosides revolve around construction of the carbocycle and the mode of nucleobase addition^18^, for example Pd-catalyzed allylic amination followed by late-stage construction of the nucleobase allows access to carbocyclic N-nucleoside derivatives^19^.

Carbocyclic C-nucleosides (CC-Ns) are rare, and this is almost certainly due to difficulties in their synthesis (Fig. 2A)^22–25^. Few methods for the synthesis of CC-Ns are known, and these methods tend to be long and non-modular. For instance, the synthesis of a potential Alzheimer’s disease drug by Merck is reported in 17 steps from D-Ribose (Fig. 2B)^23^. Here, we report a cross-coupling approach to CC-Ns which uses an asymmetric Suzuki-Miyaura type reaction as the key C–C bond forming step followed by hydroborylation-homologation-oxidation strategy to access the RNA analogs.

## Strategy

Using a cross-coupling strategy to solve the CC-N synthesis problem may be ideal as it could provide a general and modular route to access many derivatives. With the development of a catalytic asymmetric cross-coupling strategy in mind, we envisaged that coupling heteroaryl boronic acids **2** with a suitably functionalized halide such as racemic **1** could provide enantioenriched **3**. Allyl halide **1** is racemic, but is pseudo-symmetrical about the allyl halide unit, which would allow a successful desymmetrizing reaction to set the absolute and relative stereochemistry of the three contiguous stereocenters on the cyclopentene core while simultaneously forming the key C–C bond. These cyclopentenes may be suitable precursors to CC-Ns (Fig. 2C) if modification of **3** by alkene functionalization could produce RNA analogs.

Our approach relies on a catalytic asymmetric Suzuki–Miyaura coupling (SMC) reaction to make the C–C bond^26–31^. However, CC-Ns that resemble other nucleoside derivatives (c.f. Fig. 1 for example), and that we naively suspect would be more biologically interesting, would feature rather elaborate nucleobase units capable of H-bonding/acid-base interactions.

**Fig. 3:**
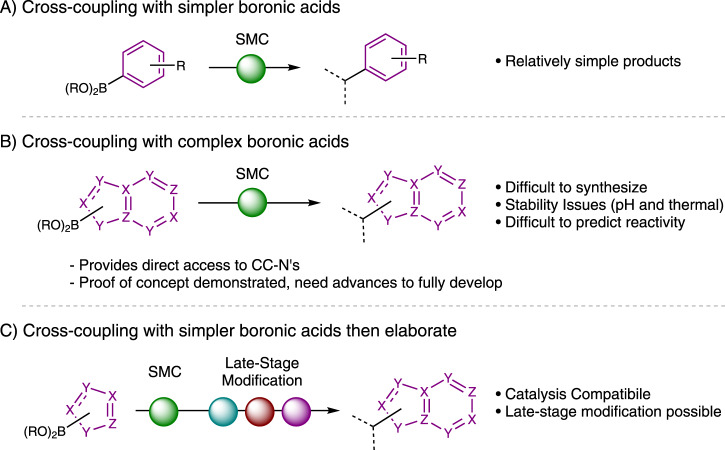
Synthetic strategies toward CC-Ns. **A** Cross-coupling with simpler boronic acids leads to simple CC-Ns. **B** Cross-coupling with complex boronic acids. **C** Cross-coupling with simpler boronic acids then elaborate to complex CC-Ns.

While we felt that relatively simple boronic acids (Fig. 3A) may be suitable for the asymmetric cross-coupling strategy to CC-Ns, the use of these coupling partners would give relatively simple (although almost unknown) products.

Accessing more typical nucleobases would require using functionalized heterocyclic boronic acids (Fig. 3B) that are significantly more complex than have been used in comparable asymmetric transformations. At the outset of this project it was not at all clear from the literature if appropriately complex boronic acid derivatives would undergo SCM reactions, what impact they would have on enantioselectivity over using benzene-derived boronic acids, or if they would even be stable^32,33^. The incompatibility of complex heterocycles with transition-metal catalyzed reactions (particularly asymmetric transformations) remains a major challenge for a number of reasons, including catalyst poisoning, undesired reactivity patterns, and proto-demetallation (or deborylation)^34–39^. The use of complex boronic acids early in the sequence would also require that the installed nucleobase units were compatible with the chemistry used to convert the cyclopentene core **3** to CC-Ns.

Another strategy to potentially access CC-Ns involves using relatively less complex boronic acids (Fig. 3C) which are designed so that late-stage modifications would reveal or allow construction of heterobase units. While this approach involves more steps than Strategy B, it has significant advantages in terms of how easy it would be to perform each step in the sequence. This would likely make Strategy C more suitable for the production of libraries of related compounds, as having to heavily optimize each individual reaction for each target molecule would be unwelcome. Despite this approach’s relative length, it would still compare favorably to known approaches to CC-Ns involving long, non-modular sequences starting from the chiral pool (c.f. Fig. 2B).

## Results and discussion

We first targeted relatively simple CC-Ns derived from the addition of six-membered rings; benzenes, pyridines and pyrimidines. Previous reports of asymmetric SMC conditions, extensively explored variables such as solvent and ligand^28^, and using these conditions addition of PhB(OH)_2_ to racemic bicycle **1** gave **3a** as a single diastereoisomer in 91% yield with very high enantioselectivity (95% ee, Table 1, Entry 1). The absolute configuration was assigned by analogy to previous work^28^. The efficiency and selectivity of this reaction, combined with previous observations that a wide variety of benzene-derived nucleophiles are generally well tolerated in related reactions, encouraged us not to dwell on simple all carbon nucleophiles and to instead explore nitrogen containing six-membered rings.

**Table 1:** Examination of pyridyl boronic acids.


Entry	Boronic acid	Product,^a^Yield (%)^b^	ee (%)
1	2a	3a, 90	95
2	2b	No conversion	–
3	2c	No conversion	–
4	2d	No conversion	–
5^c^	2e	3b, 50	94
6^c^	2e	3b, 70	94
7^c^	2f	3c, 90	92

^a^Reaction Conditions: **1** (0.4 mmol, 1 equiv), **2a** (0.8 mmol, 2 equiv), [Rh(COD)(OH)]_2_ (0.01 mmol, 2.5 mol%), ligand (0.024 mmol, 6 mol%), Aq. CsOH (0.4 mmol, 1 equiv), THF (2 mL), 60 °C, 18 h.

^b^Isolated yield.

^c^10 vol% water was used as co-solvent.

When addition of pyridines and pyrimidines was attempted no desired product was observed (Table 1, entries 2–4). We chose to next look at addition of 2-halo-pyridyl boronic acids, which have two advantages over simple pyridines, (i) that the presence of the halogen at the 2-position moderates the Lewis-basicity of the heterocycle, and (ii) that the halogen can either be used as a handle in further functionalization reactions or simply removed to reveal the parent pyridine^32,34–39^. Addition of 2-chloro-5-pyridyl boronic acid **2e** to allyl chloride **1** provided ~60% conversion to the desired product, and **3b** was isolated in 50% yield and 95% ee (Table 1, Entry 5). The reaction could be improved by adding water as co-solvent which led to the full consumption of starting material and **3b** could be isolated as a single diastereoisomer in 70% yield and 94% ee (Table 1, Entry 6). Similar reactivity was observed with 2-fluoro-5-pyridine boronic acid **2** **f** providing **3c** in 90% yield and 92% ee (Table 1, Entry 7).

## Reaction scope

**Fig. 4:**
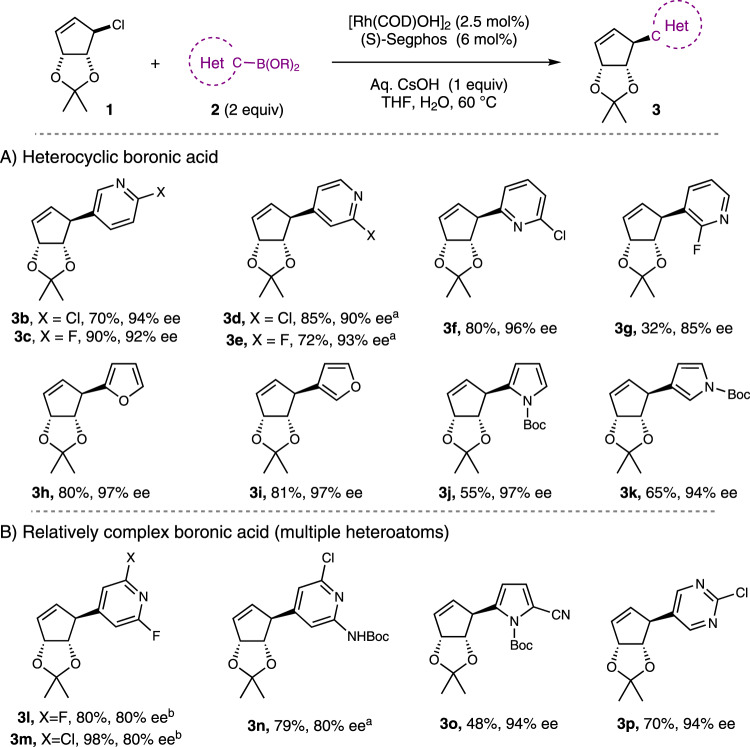
Asymmetric cross-coupling heterocyclic boronic acids with racemic **1**. **A** Heterocyclic boronic acid. **B** Relatively complex boronic acid (multiple heteroatoms). Reaction conditions: **1** (0.4 mmol, 1 equiv), **2a** (0.8 mmol, 2 equiv), [Rh(COD)(OH)]_2_ (0.01 mmol, 2.5 mol%), (*S*)-SegPhos (0.024 mmol, 6 mol%), Aq. CsOH (0.4 mmol, 1 equiv), THF (2 mL), H_2_O (0.2 mL), 60 °C, 18 h. ^a^Reaction performed at 50 °C. ^b^Reaction performed at 21 °C.

A variety of 2-halo-pyridyl boronic acid isomers were then used to give products **3b**–**g** as shown in Fig. 4. The reaction was conducted at 50 °C for 2-halo-pyridyl-4-boronic acids, and **3d** and **3e** were isolated in 85% yield and 90% ee, and 72% yield and 93% ee respectively. 2-chloro-pyridyl-6-boronic acid provided **3** **f** in 80% yield and 96% ee. Ortho-substitution of these pyridines such as 2-chloro-pyridyl-3-boronic acid, 2,6-difluoropyridyl-3-boronic acid and 2,6-difluoropyridyl-3-boronic acid gave very poor results, but we did find we could use 2-fluoro-pyridyl-3-boronic acid to obtain **3** **g** in 32% yield and 85% ee.

The use of 5-membered *O*- and *N*-containing heterocyclic nucleophiles was then explored, with 2- and 3-furan boronic acids providing **3** **h** (80%, 97% ee) and **3** **l** (81%, 97% ee). Pyrrole derived boronic acids similarly undergo highly efficient reactions to give highly enantioenriched (>94% ee) products, but **3j** and **3k** are a bit sensitive to decomposition and were isolated in 55% and 65% yield, respectively.

Encouraged by these results, we decided to explore more heavily functionalized 5- and 6-membered heterocycles. In the few examples we examined, di-halo-substituted pyridines performed poorly in reactions at 60 °C, we are unsure why but by simply doing the reaction at room temperature **3l** and **3m** were found to give a single diastereoisomer of product in >80% yield, and reasonable levels of enantioselectivity (80% ee).

A 2-chloro-aminopyridine derived boronic acid, which features both hydrogen-bond donating and accepting moieties, was also used and gave **3n** in 79% yield (80% ee). Similarly, we were able to add 2-cyano pyrrol-5-boronic acid to give **3o** (48%, 94% ee). We note that this 2-cyano pyrrole moiety is important because it is used in the late-stage construction of the pyrrolo-triazine nucleobase found in Remdesivir, and nucleobase which is otherwise difficult to make (Fig. 4)^40^.

Boronic acids featuring more than one heteroatom are a further step away from benzene rings to more elaborate cross coupling partners that are unexplored in asymmetric catalysis and have less well understood reactivity patterns in the context of asymmetric cross-coupling reactions; 2-chloro-pyrimidine-5-boronic acid was successfully employed and **3p** was isolated in 70% yield (94% ee).

The ability to run reactions on scale is tremendously important to any multi-step synthetic sequence and so we investigated the synthesis of **3h** and **3k** on a 5 mmol scale (~1 g). We found these could repeatedly make these products with consistent yields and enantioselectivities, and so these reactions – with challenging nucleophiles – should allow them to be used in the synthesis of complex products such as CC-Ns.

## Synthesis of carbocyclic C-nucleosides

**Fig. 5:**
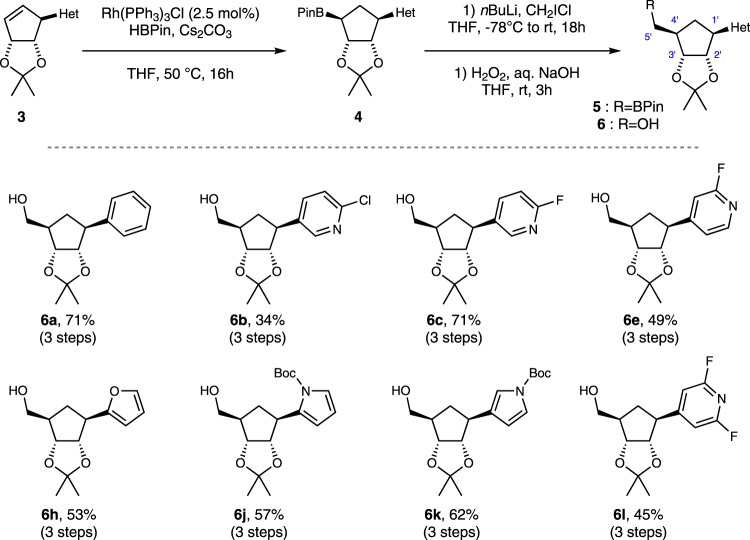
Hydroborylation and homologation of cyclopentenes **3**. Elaboration of cyclopentenes **3** to RNA analogs. Reaction Conditions - Hydroborylation: **3** (1 equiv), pinacol borane (2 equiv), [Rh(PPh_3_)Cl] (2.5 mol%), CsCO_3_ (1 equiv), THF, 50 °C, 16 h. Homologation: **4** (1 equiv), iodochloromethane (3 equiv), *n*BuLi (2.5 equiv), THF, −78 °C to rt, 18 h.

In order to produce carbocyclic ribose analogs from **3** a 5’-hydroxymethyl group must be added to the 4’ carbon in the ring (ribose numbering). From the carbocyclic alkene in **3** both the regiochemistry (attachment at the 4’ position) and stereochemistry (*cis* or *trans* relative to the nucleobase) must be controlled with the *cis* or β-stereochemistry desired as it mimics the stereochemistry most often found in natural nucleosides. After considerable exploratory work, including examining metal catalyzed direct carbonylation and carboxylation, hydrozirconation followed by trapping and photochemical approaches, we were able to identify a useful 3-step hydroborylation-homologation-oxidation sequence in order to add the hydroxymethyl group with complete stereo- and regio-chemical control in roughly 50% yield (Fig. 5) – however it does involve the use of fairly reactive species and so it would not be expected to be compatible with all functional groups.

The homologation first uses Wilkinson’s catalyst (2.5 mol%) with pinacol borane to give boronic ester **4** as a single isomer. The presence of cesium carbonate is crucial for the suppression of competitive and undesired alkene reduction. Matteson homologation using *n*-BuLi and CH_2_ICl was then used to convert the secondary boronic ester **4** to primary ester **5**, and subsequent hydrogen peroxide oxidation furnished alcohol **6** as a key CC-N precursor (Fig. 5). The route successfully furnished phenyl (**6a**), fluoropyridines (**6c**, **6e**, **6l**), furan (**6h**) and pyrrole (**6j** and **6k**) derivatives in high yields. However, most chloropyridines (except **6b**) and the cyano-pyrrole decomposed during attempted hydroborylation. The hydroborylation-homologation-oxidation sequence was successfully performed on 4 mmol scales to give **6h** and **6k**.

**Fig. 6:**
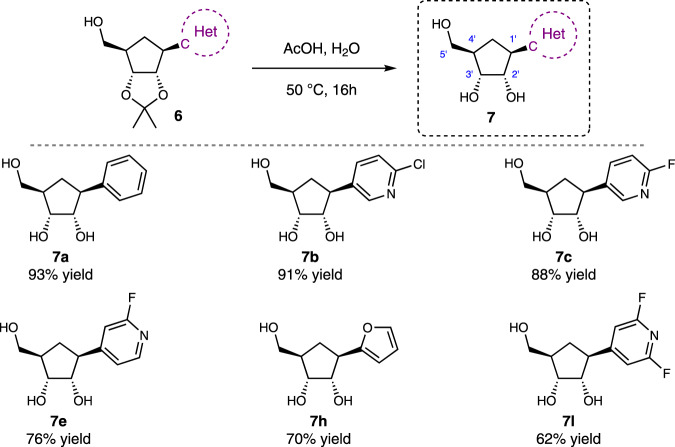
Carbocyclic C-nucleosides bearing relatively simple ‘nucleobase’ moieties. Access toward simpler CC-Ns featuring phenyl, halo-pyridines, and furan nucleobase.

By performing a final acetonide deprotection under standard acidic conditions (Fig. 6), a series of phenyl, pyridine and furan derived CC-Ns (**7a**–**7c**, **7e**, **7h**, and **7k**) was isolated in high yields.

Encouraged by the success in obtaining a collection of CC-Ns bearing simple nucleobase moieties we became interested in the problem of how to make more elaborate CC-Ns. We started preparing some complex heterocyclic boronic acids and exploring their synthesis and compatibility with the SMC reaction, (for example see SI; See supplemantry data for (a) Attepmts toward complex boronic acids. (b) Detailed synthetic schemes and procedures) Overall, we found that a wide selection of what we imagined to be suitably complex boronic acids (and corresponding esters) were difficult to prepare and not very stable.

**Fig. 7:**
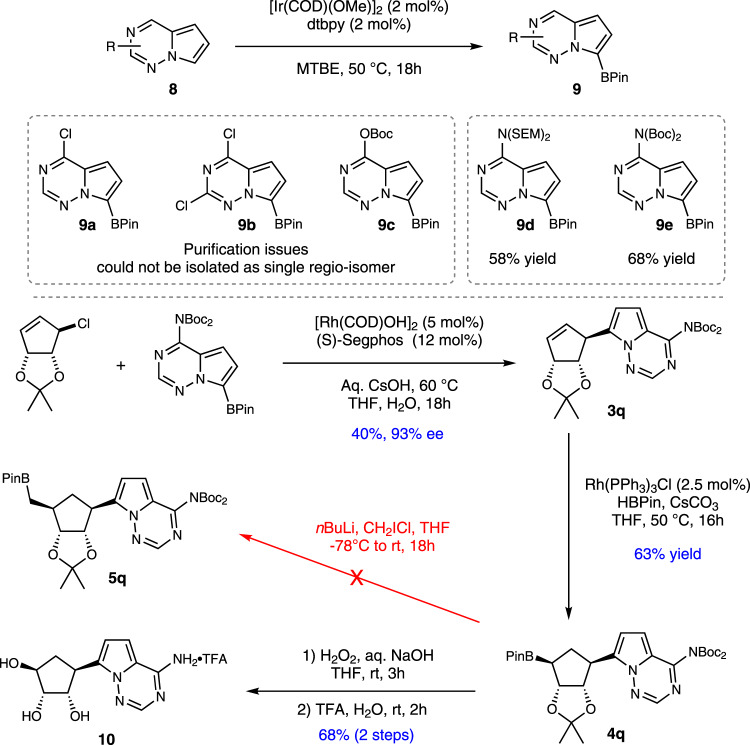
Attempts toward complex CC-Ns. Cross-coupling attempts with pyrrolo-trazine boronic acids.

In the absence of a specific target molecule that would provide the motivation to overcome stability problems we decided to focus our efforts on nucleobase **9**, featured in Remdesivir (Fig. 1). These pyrrolo-triazines contain 4 nitrogen atoms, and were additionally attractive because boronic ester **9d** had been reported and seemed easy to prepare^25^. Our first attempts at making suitable boronic acid derivatives were based on the above success with 2-halo-substitution, and we chose to examine the synthesis of **9a** and **9b**. While a halogen-lithium exchange approach was unsuccessful, presumably due to incompatibility with nBuLi, we were able to make boronic esters using Ir-catalyzed C-H borylation^41–43^. However, borylation to give **9a-c** was complicated because mixture of regioisomers were obtained which proved difficult to separate. Eventually, we found that **9d** and **9e** could be prepared as single isomers and these were examined in the asymmetric cross-coupling reaction. Repeated attempts to add **9d** gave poor conversion, however when using **9e** (Fig. 7) we obtained **3q** in 40% yield and 93% ee when doubling the normal catalyst loading.

The pyrrolo-triazine carbocycle **3q** underwent hydroborylation to **4q**, but attempts to homologate this boronic ester to add the hydroxymethyl group were unsuccessful and gave decomposition products, almost certainly due to incompatibility of Matteson homologation using *n*-BuLi and CH_2_ICl with the rich array of nitrogen atoms in **4q**. We were able to access carbocyclic triol **10** however by oxidation followed by trifloroacetic acid mediated global deprotection to give the TFA salt in 43% yield over 3 steps (Fig. 7).

Overall, the synthesis of complex boronic acids featuring nucleobases was not only difficult, but they were more often than not either inherently unstable or incompatible with the relatively mild conditions of the asymmetric SMC reaction. Most of the complex boronic acids led to protodeborylation and it was difficult to see any obvious correlation between heterocycle structure and boronic acid stability/reactivity.

As the approach (Fig. 3: Strategy 2) of adding highly complex boronic acids proved to be difficult and would likely require extensive optimization in cases where it was viable, and the addition of the 5’ hydroxyl methyl group also appeared challenging in the presence of complex heterocycles we decided to pursue the alternative approach (Fig. 3: Strategy 3) of adding relatively simple boronic acids which could later be transformed into more elaborate units.

**Fig. 8:**
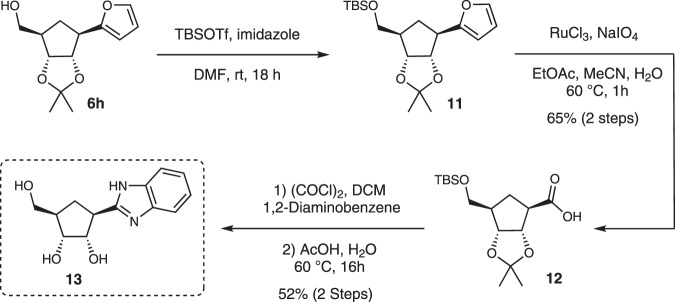
Late-stage modification strategy. Modification of furan to benzaimidazole derived CC-N.

We envisaged that the oxidative cleavage of furan could provide carboxylic acid moiety primed for the construction of complex heterocycles. Alcohol **6h** was first TBS-protected and then oxidative cleavage of furan moiety was accomplished by the combination of ruthenium chloride (10 mol%) and sodium periodate. The resulting carboxylic acid **12** was isolated in 65% yield over 2 steps (Fig. 8). As a simple proof of concept of this approach we chose to make the benzoimidazole derived carbocyclic C-nucleoside **13** which was accomplished via in situ generation of the corresponding acid chloride, and addition of 1,2-diaminobenzene to form the amide. Acetic acid mediated cyclization and global deprotection gave CC-N **11** in 52% isolated yield from **12** (Fig. 8). We note that carboxylic acids can readily be transformed into a whole range of structures and so it seems very likely that intermediate **12**^44^ could also be used to access many other complex CC-Ns.

**Fig. 9:**
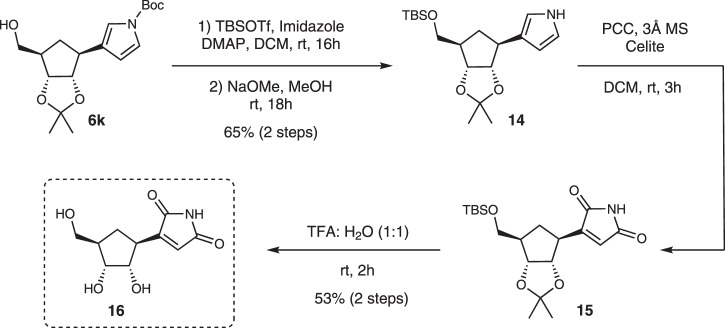
Synthesis of carbocyclic Showdomycin. Post-synthetic modification of Pyrrole to access carbocyclic analog of Showdomycin.

Showdomycin is a C-nucleoside with antiviral, antibacterial and antitumor properties^22,45,46^. We chose to produce a carbocyclic analog of Showdomycin in order to showcase our approach to enantiomerically enriched CC-N derivatives of natural products with important biological activity (Fig. 9). After hydroborylation-homologation-oxidation sequence of **3k** (94% ee) to **6k** (Fig. 5, 62% yield over three steps), the alcohol was subjected to TBS protection followed by pyrrole Boc-deprotection to give **14** (65% yield over 2 steps). Pyridinium chlorochromate (PCC) oxidation of the pyrrole group followed by deprotection with trifluoroacetic acid gave carbocyclic Showdomycin analog **16** in 53% isolated yield over 2 steps (Fig. 9).

## Conclusions

We present new synthetic strategies for the synthesis of carbocyclic C-nucleosides. The approaches use a key asymmetric Suzuki-Miyaura-type coupling reaction followed by late-stage addition of the hydroxymethyl group to give ribose analogs. Using relatively simple boronic acids nucleophiles Strategy A provides a route to simple CC-N’s featuring benzene derivatives or heterocycles featuring a single heteroatom in the ‘nucleobase’ moiety. We identified two strategies (B and C, Fig. 3) which could produce more complex CC-Ns.

While strategy B would provide direct access to complex CC-N’s by using complex boronic acid derivatives, these nucleophiles are difficult to prepare, and if they are compatible with the sequence then it seems very likely that optimization of many of the individual steps would be required to prepare each substrate (Fig. 7).

We have demonstrated that strategy C is capable of producing CC-Ns featuring more complex nucleobases including the carbocyclic analog of Showdomycin, a biologically active natural product. Strategy C involves the addition of relatively less complex boronic acids followed by late-stage modification, and therefore requires more steps than strategy B but we would recommend this approach to anyone seeking to produce more complex CC-N’s in the future (although there may of course be shorter alternative routes that can be devised to access a particular target); carboxylic acid **12** can obviously be converted into a wide range of structures and there are many relatively simple heterocycles available which would be expected to be compatible with our sequence and could very likely be converted into complex CC-N targets.

## Methods

### General procedure for enantioselective Suzuki–Miyaura coupling

[Rh(COD)OH]_2_ (4.6 mg, 0.010 mmol, 2.5 mol%) and (S)-Segphos (14.6 mg, 0.026 mmol, 6.0 mol%) were added to a 7 mL dram vial, sealed with a rubber septum under an argon atmosphere, dissolved in THF (0.80 mL) and stirred at 60 °C. After 30 min, a solution (or suspension) of boronic acid (0.80 mmol, 2.0 equiv) and allylic chloride (62 µL, 0.40 mmol, 1.0 equiv) in THF (0.8 mL) and H_2_O (0.2 mL) was added via syringe and the flask was rinsed with THF (0.4 mL). Lastly, CsOH (50 wt% aq. solution, 70 µL, 0.40 mmol, 1.00 equiv) was added and the resulting mixture was then stirred at indicted temperature for the period of time indicated. The mixture was then cooled to room temperature and diluted with Et_2_O (2 mL) before passing through a plug of SiO_2_. The plug was washed with an additional 10 mL of Et_2_O and the solvents were removed in vacuo. Purification by flash chromatography afforded the desired product **3**.

### General procedure for synthesis of **7a**–**l**

[Rh(PPh_3_)_3_Cl] (23.1 mg, 0.025 mmol, 2.5 mol%) and Cs_2_CO_3_ (325.8 mg, 1.0 mmol, 1 equiv) were added to a flame dried 25 mL round bottom flask, sealed with a rubber septum under an argon atmosphere, dissolved in THF (0.5 mL) and stirred at room temperature. After 5 min, a solution (or suspension) of **3** (1 mmol, 1.0 equiv) in THF (3 mL) was added via syringe and the flask was rinsed with THF (0.5 mL). Pinacolborane (0.29 mL, 2 mmol, 2.0 equiv) was added dropwise to the reaction mixture at 50 °C and stirred for 16 h at 50 °C. The mixture was then cooled to room temperature and diluted with Et_2_O (5 mL) before passing through a plug of silica. The plug was washed with an additional 10 mL of Et_2_O and the solvents were removed in vacuo. The crude product boronic acid pinacol ester **4** was charged to the next step without further purification.

In a flamed dried flask under nitrogen, *n*-butyllithium (2.5 M in hexanes, 0.75 mL, 1.88 mmol, 2.5 equiv) was added dropwise to a solution of boronic acid pinacol ester **4** obtained above (0.75 mmol, 1.0 equiv) and iodochloromethane (165 µL, 2.25 mmol, 3 equiv) in THF (3.5 mL) at −78 °C. The reaction mixture was slowly allowed to warm to ambient temperature over 12 h. The mixture was diluted with Et_2_O (5 mL) and the organic layer was washed with an aq. sat. solution of NH_4_Cl (2 × 3 mL) and dried over Na_2_SO_4_, filtered, and the solvent was removed in vacuo. The crude product boronic acid pinacol ester **5** was charged to the next step.

30%wt. H_2_O_2_ (1 mL) was added to a solution of boronic acid pinacol ester **5** obtained above (0.75 mmol, 1.0 equiv) and 2 N aq. NaOH (1.5 ml) in THF (4 mL) at 0 °C. The reaction mixture was slowly allowed to warm to ambient temperature. After 3 h, the mixture was diluted with Et_2_O (7 mL). The organic layer was separated and the aqueous layer was extracted with Et_2_O (2 × 5 mL). The combined organic layers were dried over anhydrous Na_2_SO_4_, filtered, and the solvent was removed in vacuo. Purification by flash chromatography afforded alcohol **6**.

A solution of alcohol **6** (0.2 mmol) in AcOH (0.5 mL) and H_2_O (0.5 mL) was stirred at 50 °C for 16 h. The reaction mixture was concentrated under reduced pressure and purification by flash chromatography afforded compound **7**.

### Supplementary information

Supplementary Information

Description of Additional Supplementary Files

Supplementary data 1

## Data Availability

The authors declare that supporting information containing experimental procedures, compound synthesis and characterization, and supporting discussion are available within the paper and its supplementary data files (Supplementary Methods and Supplementary Spectra).
