# Supplementary material for: Catalytic asymmetric synthesis of carbocyclic C-nucleosides
Source: Commun Chem. 2022 Nov 19;5:154. doi: 10.1038/s42004-022-00773-6 (PMC9676730; doi:10.1038/s42004-022-00773-6)
Supplement: Supplementary file 1 — Supplementary Information [file 42004_2022_773_MOESM1_ESM.pdf]

Catalytic Asymmetric Synthesis of Carbocyclic C-Nucleosides

Sourabh Mishra, Florian C. T. Modicom, Conor L. Dean, Stephen P. Fletcher*

Department of Chemistry, Chemistry Research Laboratory, University of Oxford, Oxford OX1 3TA, UK

Supporting Information – Experimental

Table of Contents

1. General Information 2

2. General Procedure for Enantioselective Suzuki-Miyaura Coupling 3

3. Synthesis of Carbocyclic C-Nucleosides (CC-Ns) 22

4. Suzuki-Miyaura Coupling Attempts with Complex Boronic Acids 38

5. Synthesis of Amino Pyrrolo Triazine Derived CC-Ns 38

6. Synthesis of Benzo-Imidazole Derived CC-Ns 42

7. Synthesis of Carbocyclic Showdomycin 16 45

8. Supplementary References 47

Supplementary Methods

1. General Information

All reactions were carried out in anhydrous solvents with continuous magnetic stirring under an inert argon atmosphere. Heating was performed using DrySyn heating blocks.

Nuclear magnetic resonance (NMR) spectroscopy measurements were carried out at room temperature. ^{1}H NMR, ^{13}C NMR, ^{19}F NMR, COSY, HSQC, HMBC and NOESY experiments were carried out using Bruker AVIII HD 400 (400/100 MHz) or AVIII HD 400 (500/125 MHz) spectrometers. Chemical shifts (δ) are reported in ppm relative to the residual solvent peak with corresponding coupling constants (*J*) in Hertz (Hz) and multiplicities (s: singlet, d: doublet, t: triplet, q: quartet, m: multiplet and combinations of these). Assignment follows HSQC, COSY, HMBC or/and NOESY spectra, chemical shift and coupling constant analysis.

Optical rotations ($[\alpha]_{25}^{D}$) were recorded using a Perkin Elmer-241 Polarimeter. Concentrations (c) are reported in g/100 mL.

Chiral SFC (supercritical fluid chromatography) separations were conducted on a Waters Acquity UPC2 system using Waters Empower software. Chiralpak columns (150x3 mm, particle size 3 µm) were used as specified in the text. Solvents used were of HPLC grade (Fisher Scientific, Sigma Aldrich or Rathburn).

High Resolution Mass spectra were carried out by internal service at the University of Oxford. (1) Electron spray ionisation (ESI^{+}) was recorded on a Fisons Platform II. (2) Electron ionisation (EI)/Chemical ionisation (CI): Analyses were performed on an Agilent 7200 quadrupole time of flight (Q-ToF) instrument equipped with a direct insertion probe supplied by Scientific instrument Manufacturer (SIM) GmbH. (3) Atmospheric pressure chemical ionisation ($APCI^{+}$): Analyses were performed using a Thermo Exactive mass spectrometer equipped with Waters Acquity liquid chromatography system.

Commercially available reagents and ligands were purchased from Sigma Aldrich, Alfa Aesar, Acros Organics, Fluorochem and Strem Chemicals and were used without further purification unless stated otherwise. $[Rh(cod)OH]_2$ was bought from Sigma Aldrich. All aryl and heteroarylboronic acids were purchased and used without additional purification unless stated otherwise. Dry solvents were purchased from Thermo Scientific™, Extra Dry over Molecular Sieve, Stabilized, AcroSeal™ and were degassed with argon prior to usage. Deuterated solvents were purchased from Sigma Aldrich. Allyl chloride 1 (4-chloro-2,2-dimethyl-3a,6a-dihydro-4H-cyclopenta [d] [1,3] dioxole) was prepared according to a literature procedure.[1,2]

2. General Procedure for Enantioselective Suzuki-Miyaura Coupling

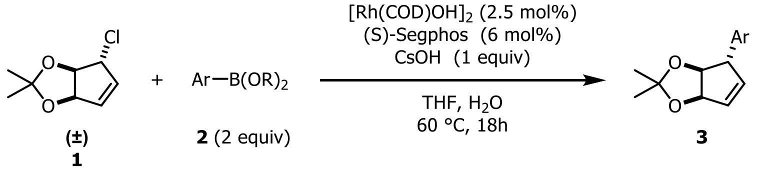

$[Rh(cod)OH]_2$ (4.6 mg, 0.010 mmol, 2.5 mol%) and (S)-Segphos (14.6 mg, 0.026 mmol, 6.0 mol%) were added to a 7 mL dram vial, sealed with a rubber septum under an argon atmosphere, dissolved in THF (0.80 mL) and stirred at 60 °C. After 30 min, a solution (or suspension) of boronic acid (0.80 mmol, 2.0 equiv) and allylic chloride (62 µL, 0.40 mmol, 1.0 equiv) in THF (0.8 mL) and H_2O (0.2 mL) was added via syringe and the flask was rinsed with THF (0.4 mL). Lastly, CsOH (50 wt% aq. solution, 70 µL, 0.40 mmol, 1.00 equiv) was added and the resulting mixture was then stirred at indicted temperature for the period of time indicated. The mixture was then cooled to room temperature and diluted with Et_2O (2 mL) before passing through a plug of SiO_2. The plug was washed with an additional 10 mL of Et_2O and the solvents were removed in vacuo. Purification by flash chromatography afforded the desired product.

Racemates: Racemic samples were synthesized with (±)-BINAP instead of (S)-Segphos.

Upscale: Larger-scale experiments (up to 5 mmol scale) were performed in direct analogy to the general procedure A.

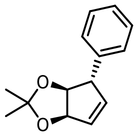

(3a*S*,4*S*,6a*R*)-2,2-dimethyl-4-phenyl-3a,6a-dihydro-4H-cyclopenta [d] [1,3] dioxole (3a). The corresponding compound was prepared following general procedure **A** using phenylboronic acid. The mixture was stirred at 60 °C for 18 hours. Purification by flash chromatography (5% Et_2O/petrol to 10% Et_2O/petrol) afforded compound **3a** as a colourless oil (91% yield, 94% ee) as a single diastereomer (dr >20:1).

^{1}H NMR ($CDCl_3$, 400 MHz): δ (ppm) 7.37 – 7.29 (m, 2H), 7.26 – 7.21 (m, 1H), 7.17 – 7.05 (m, 2H), 6.00 (dt, J = 5.7, 1.8 Hz, 1H), 5.97 – 5.82 (m, 1H), 5.31 (dq, J = 5.9, 1.4 Hz, 1H), 4.54 (d, J = 5.7 Hz, 1H), 4.04 (d, J = 2.1 Hz, 1H), 1.49 (s, 3H), 1.34 (s, 3H). The spectroscopic data satisfactorily matched previously reported data.[1]

SFC Conditions: Chiralpak ID; 1500 PSI, 30 °C; flow: 1.5 mL/min; from 1% to 30% MeOH in 5 min; 97:3 er (major enantiomer t_R = 1.20 min; minor enantiomer t_R = 1.38 min), **94% ee**. **$[\alpha]^{25}_D$** = – 242.2 (c = 1.0, $CHCl_3$).

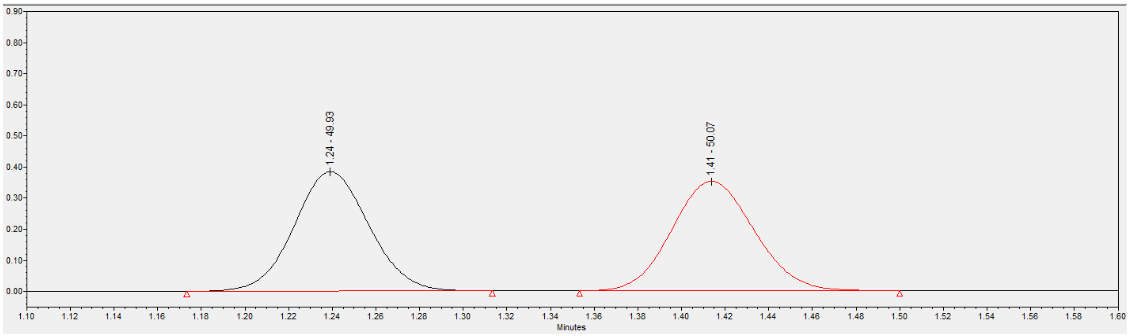

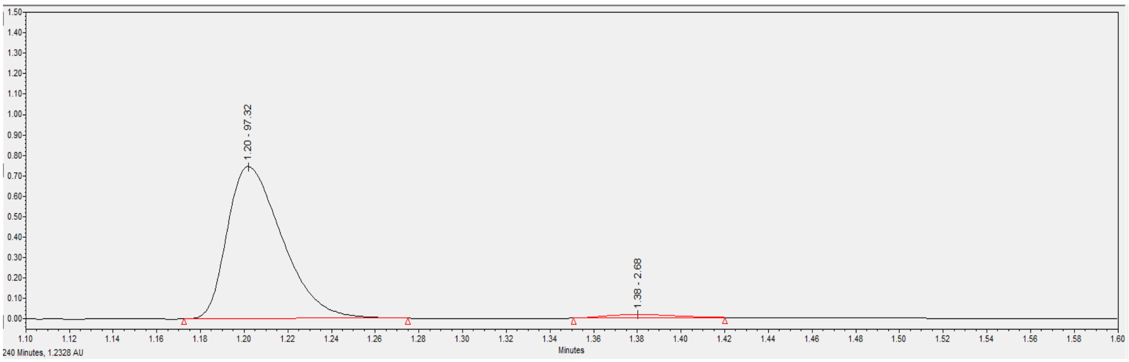

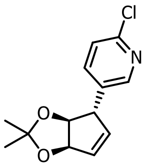

2-chloro-5-((3aS,4S,6aR)-2,2-dimethyl-3a,6a-dihydro-4H-cyclopenta [d] [1,3] dioxol-4-yl) pyridine (3b). The corresponding compound was prepared following general procedure **A** using (6-chloropyridin-3-yl) boronic acid. The mixture was stirred at 60 °C for 18 hours. Purification by flash chromatography (10% Et_2O/petrol to 25% Et_2O/petrol) afforded compound **3b** as a colourless oil (70% yield, 94% ee) as a single diastereomer (dr >20:1).

^{1}H NMR ($CDCl_3$, 400 MHz): δ (ppm) 8.21 (d, J = 2.5 Hz, 1H), 7.38 (dd, J = 8.2, 2.5 Hz, 1H), 7.28 (d, J = 8.2 Hz, 1H), 6.07 (dt, J = 5.9, 1.7 Hz, 1H), 5.89 (ddd, J = 5.7, 2.5, 1.0 Hz, 1H), 5.31 (dq, J = 5.7, 1.4 Hz, 1H), 4.49 (d, J = 5.7 Hz, 1H), 4.04 (s, 1H), 1.48 (s, 3H), 1.35 (s, 3H). The spectroscopic data satisfactorily matched previously reported data.[1]

SFC Conditions: Chiralpak IF; 1500 PSI, 30 °C; flow: 1.5 mL/min; from 1% to 30% MeOH in 5 min; 97:3 er (major enantiomer t_R = 3.94 min; minor enantiomer t_R = 3.76 min), **94% ee**. $[\alpha]^{25}_D$ = –210.5 (c = 1.0, $CHCl_3$).

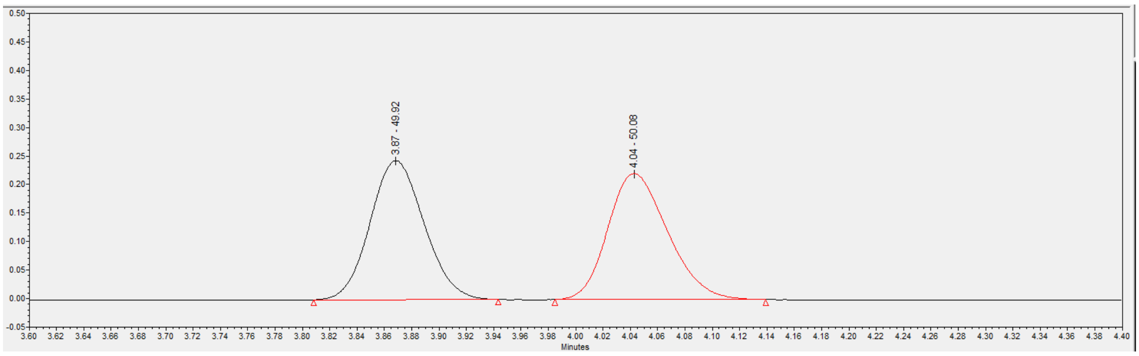

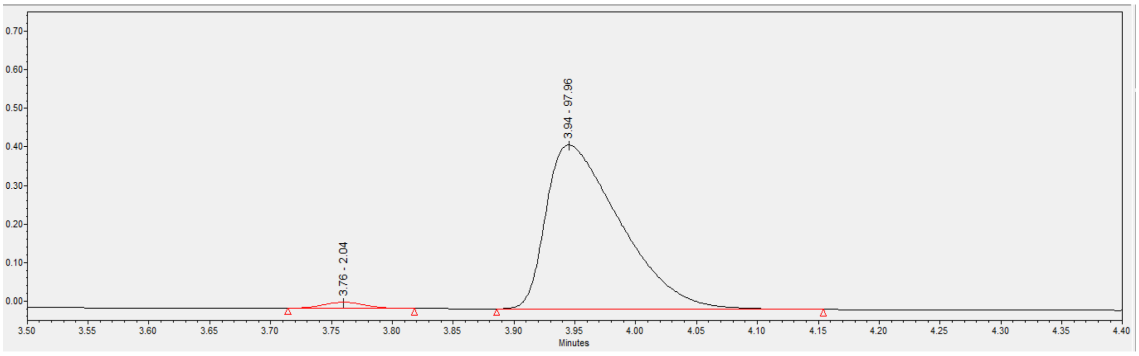

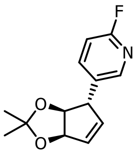

2-fluoro-5-((3aS,4S,6aR)-2,2-dimethyl-3a,6a-dihydro-4H-cyclopenta [d] [1,3] dioxol-4-yl) pyridine (3c). The corresponding compound was prepared following general procedure **X** using (6-fluoropyridin-3-yl) boronic acid. The mixture was stirred at 60 °C for 18 hours. Purification by flash chromatography (10% Et_2O/petrol to 25% Et_2O/petrol) afforded compound **3c** as a colourless oil (90% yield, 92% ee) as a single diastereomer (dr >20:1).

^{1}H NMR ($CDCl_3$, 400 MHz): δ (ppm) 8.01 (d, *J* = 2.5 Hz, 1H), 7.50 (td, *J* = 8.2, 2.5 Hz, 1H), 6.87 (dd, *J* = 8.2, 2.5 Hz, 1H), 6.05 (dt, *J* = 5.8, 1.9 Hz, 1H), 5.90 – 5.87 (m, 1H), 5.30 (d, *J* = 5.7 Hz, 1H), 4.47 (d, *J* = 5.7 Hz, 1H), 4.05 (s, 1H), 1.47 (s, 3H), 1.34 (s, 3H). **^{13}C NMR** (101 MHz, $CDCl_3$): δ (ppm) 162.9 (d, *J* = 238.6 Hz), 146.7 (d, *J* = 14.7 Hz), 140.3 (d, *J* = 7.7 Hz), 134.8 (d, *J* = 4.7 Hz), 134.5, 133.0, 111.0, 109.6 (d, *J* = 37.5 Hz), 85.7, 85.3, 54.8, 27.5, 25.8. **^{19}F NMR** (376 MHz, $CDCl_3$) δ –70.5 (d, *J* = 4.8 Hz).

HRMS (ESI): m/z calcd for $C_{13}H_{15}O_2NF^+$ $[M + H]^+$ 236.1081 found 236.1081.

IR (ν_{max}/cm^{-1}) 2981, 2916, 2890, 2360, 2341, 1597, 1490, 1396, 1381, 1251, 1214, 1159, 1075, 1052, 954, 892, 863, 833, 803, 767, 732, 812, 757, 731, 771, 759, 715.

SFC Conditions: Chiralpak IF; 1500 PSI, 30 °C; flow: 1.5 mL/min; from 1% to 30% MeOH in 5 min; 96:4 er (major enantiomer t_R = 2.09 min; minor enantiomer t_R = 2.47 min), **92% ee**. **$[\alpha]^{25}_D$** = –156.3 (c = 1.0, $CHCl_3$).

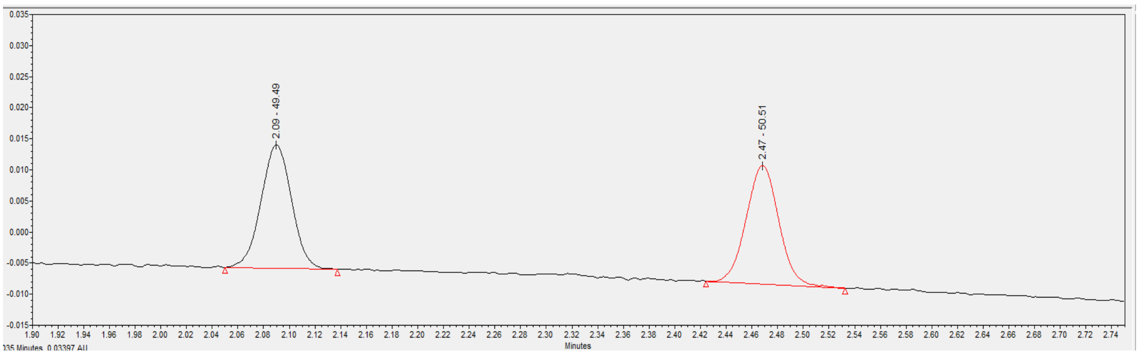

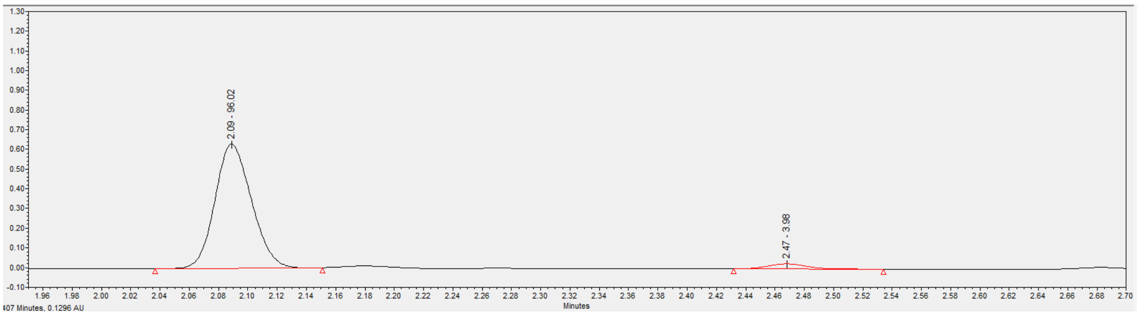

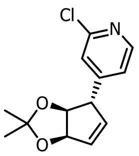

4-((3aS,4S,6aR)-2,2-dimethyl-3a,6a-dihydro-4H-cyclopenta [d] [1,3] dioxol-4-yl)-2-chloro pyridine (3d). The corresponding compound was prepared following general procedure **A** using (2-chloropyridin-4-yl) boronic acid. The mixture was stirred at 50 °C for 24 hours. Purification by flash chromatography (10% Et_2O/petrol to 25% Et_2O/petrol) afforded compound **3d** as a colourless oil (79% yield, 90% ee) as a single diastereomer (dr >20:1).

^{1}H NMR ($CDCl_3$, 400 MHz): δ (ppm) 8.30 (d, *J* = 5.2 Hz, 1H), 7.08 (d, *J* = 1.5 Hz, 1H), 7.00 (dd, *J* = 5.2, 1.5 Hz, 1H), 6.08 (dt, *J* = 5.8, 1.6 Hz, 1H), 5.86 (dd, *J* = 5.8, 2.4 Hz, 1H), 5.28 (dd, *J* = 5.8, 1.6 Hz, 1H), 4.49 (d, *J* = 5.8 Hz, 1H), 3.99 (s, 1H), 1.46 (s, 3H), 1.33 (s, 3H).

^{13}C NMR (101 MHz, $CDCl_3$): δ (ppm) 154.0, 152.2, 150.1, 133.9, 133.6, 123.3, 121.8, 111.2, 85.19, 85.18, 57.3, 27.5, 25.8.

HRMS (ESI): m/z calcd for $C_{13}H_{15}O_2NCl^+$ $[M + H]^+$ 252.0786 found 252.0786.

IR (ν_{max}/cm^{-1}) 2981, 2938, 2890, 2361, 2341, 1592, 1546, 1463, 1386, 1250, 1213, 1158, 1126, 1076, 1052, 991, 956, 864, 843, 803, 692.

SFC Conditions: Chiralpak IF; 1500 PSI, 30 °C; flow: 1.5 mL/min; from 1% to 30% MeOH in 5 min; 95:5 er (major enantiomer t_R = 2.43 min; minor enantiomer t_R = 3.12 min), **90% ee**. **$[\alpha]^{25}_D$** = –277.2 (c = 1.0, $CHCl_3$).

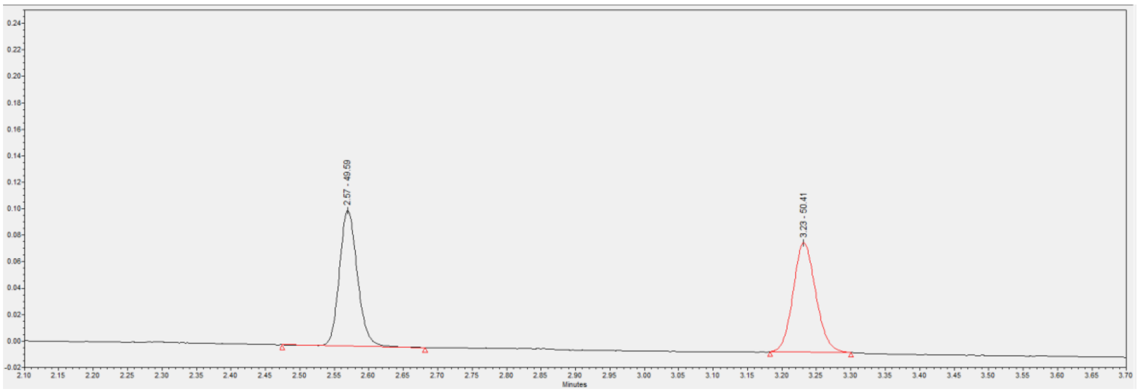

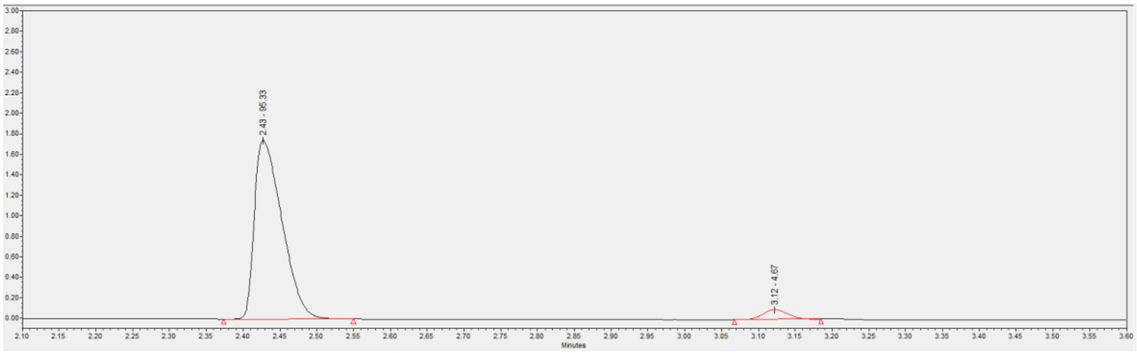

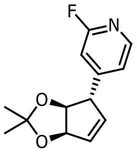

4-((3aS,4S,6aR)-2,2-dimethyl-3a,6a-dihydro-4H-cyclopenta [d] [1,3] dioxol-4-yl)-2-fluoro pyridine (3e). The corresponding compound was prepared following general procedure **A** using (2-fluoropyridin-4-yl) boronic acid. The mixture was stirred at rt for 24 hours. Purification by flash chromatography (10% Et_2O/petrol to 25% Et_2O/petrol) afforded compound **3e** as a colourless oil (72% yield, 93% ee) as a single diastereomer (dr >20:1).

^{1}H NMR ($CDCl_3$, 400 MHz): δ (ppm) 8.13 (d, *J* = 5.1 Hz, 1H), 6.96 (dt, *J* = 5.1, 1.7 Hz, 1H), 6.68 (t, *J* = 1.7 Hz, 1H), 6.08 (dt, *J* = 5.7, 1.6 Hz, 1H), 5.89 – 5.87 (m, 1H), 5.29 (dq, *J* = 5.8, 1.6 Hz, 1H), 4.50 (d, *J* = 5.8 Hz, 1H), 4.04 (s, 1H), 1.46 (s, 3H), 1.33 (s, 3H). **^{13}C NMR** (101 MHz, $CDCl_3$): δ (ppm) 164.4 (d, *J* = 239.4 Hz), 156.6 (d, *J* = 7.6 Hz), 148.1 (d, *J* = 15.2 Hz), 133.8, 133.6, 120.7 (d, *J* = 4.0 Hz), 111.1, 108.4 (d, *J* = 37.4 Hz), 85.2, 85.2, 57.4 (d, *J* = 2.9 Hz), 27.5, 25.8. **^{19}F NMR** (376 MHz, $CDCl_3$) δ –67.8.

HRMS (ESI): m/z calcd for $C_{13}H_{15}O_2NF^+$ $[M + H]^+$ 236.1081 found 236.1081.

IR (ν_{max}/cm^{-1}) 2988, 2935, 1737, 1610, 1566, 1556, 1481, 1457, 1413, 1372, 1289, 1268, 1248, 1211, 1158, 1109, 1076, 1050, 997, 975, 868, 840, 793, 773.

SFC Conditions: Chiralpak IC; 1500 PSI, 30 °C; flow: 1.5 mL/min; from 1% to 30% MeOH in 5 min; 96.5:3.5 er (major enantiomer t_R = 2.18 min; minor enantiomer t_R = 2.67 min), **93% ee**. **$[\alpha]^{25}_D$** = –182.7 (c = 1.0, $CHCl_3$).

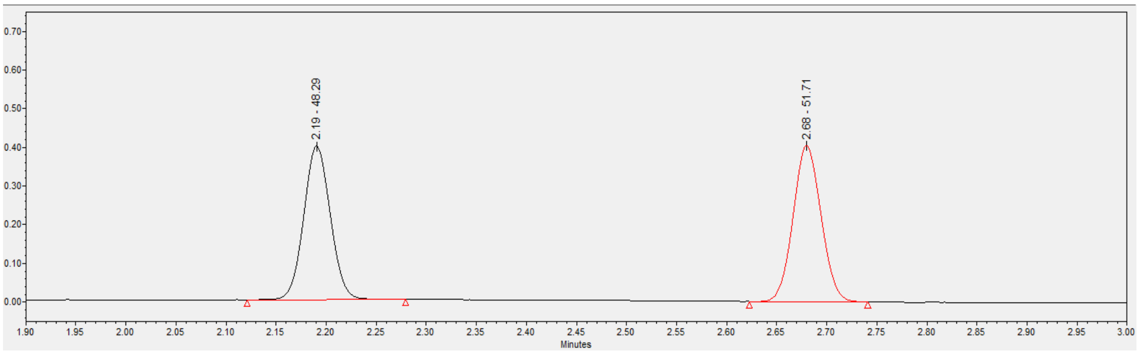

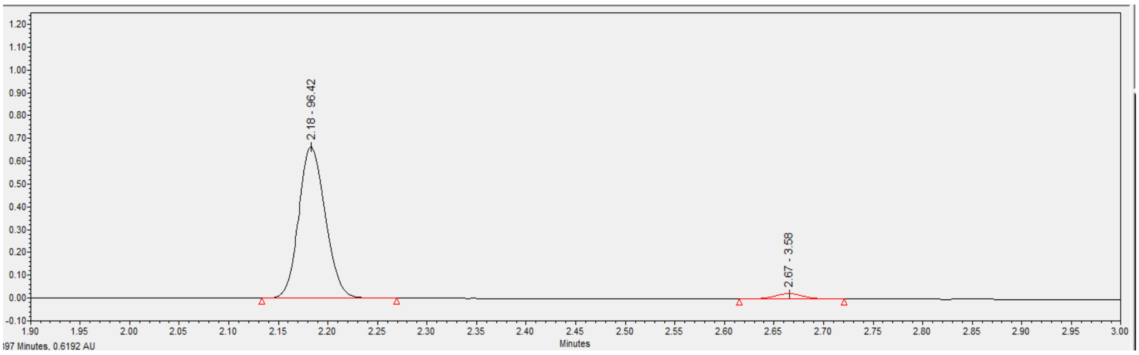

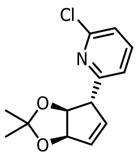

2-chloro-6-((3aS,4S,6aR)-2,2-dimethyl-3a,6a-dihydro-4H-cyclopenta [d] [1,3] dioxol-4-yl) pyridine (3f). The corresponding compound was prepared following general procedure **A** using 3-chloro-2-pyridylboronic acid. The mixture was stirred at 60 °C for 18 hours. Purification by flash chromatography (10% Et_2O/petrol to 25% Et_2O/petrol) afforded compound **3f** as a colourless oil (80% yield, 96% ee) as a single diastereomer (dr >20:1).

^{1}H NMR ($CDCl_3$, 400 MHz): δ (ppm) 7.01 (t, *J* = 1.2 Hz, 1H), 7.17 (dd, *J* = 7.9, 0.8 Hz, 1H), 7.06 (dd, *J* = 7.5, 0.8 Hz, 1H), 6.00 (dt, *J* = 5.7, 1.9 Hz, 1H), 5.90 (ddt, *J* = 5.7, 2.5, 0.9 Hz, 1H), 5.36 (ddt, *J* = 5.8, 1.6, 0.9 Hz, 1H), 4.80 (dt, *J* = 5.8, 1.1 Hz, 1H), 4.15 (tt, *J* = 2.6, 1.4 Hz, 1H), 1.47 (d, *J* = 0.7 Hz, 3H), 1.36 (d, *J* = 0.7 Hz, 3H). **^{13}C NMR** (101 MHz, $CDCl_3$): δ (ppm) 162.2, 151.5, 139.2, 134.4, 132.9, 122.5, 120.7, 110.7, 85.7, 84.2, 59.8, 27.6, 26.0.

HRMS (ESI): m/z calcd for $C_{13}H_{14}O_2NClNa^+$ $[M + Na]^+$ 274.0605 found 274.0605.

IR (ν_{max}/cm^{-1}) 2981, 2918, 2360, 2341, 1741, 1604, 1578, 1381, 1250, 1214, 1160, 1076, 1052, 955, 893, 865, 803, 760, 733, 800, 753, 712.

SFC Conditions: Chiralpak IG; 1500 PSI, 30 °C; flow: 1.5 mL/min; from 1% to 30% MeOH in 5 min; 98:2 er (major enantiomer t_R = 2.82 min; minor enantiomer tR = 3.15 min), **96% ee**. **$[\alpha]^{25}_D$** = –189.6 (c = 1.0, $CHCl_3$).

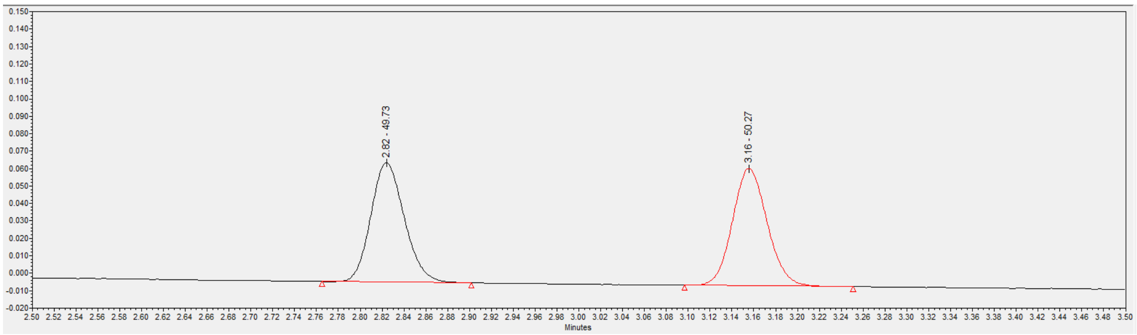

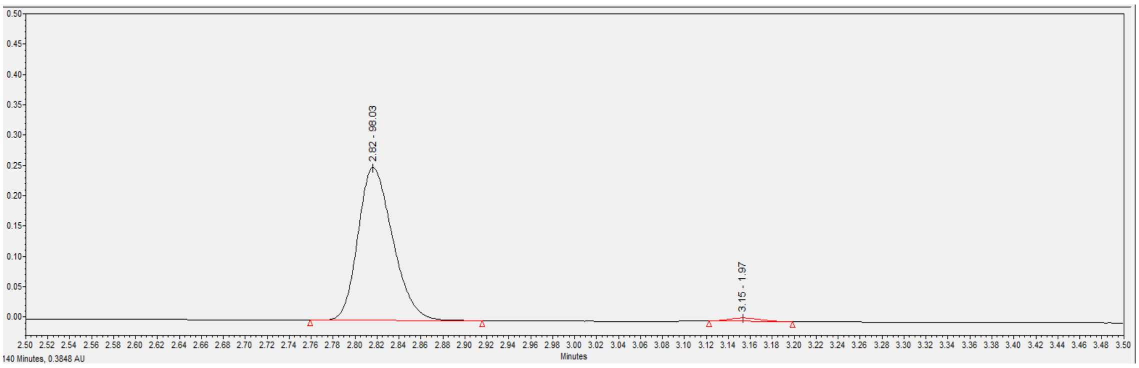

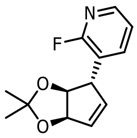

2-((3aS,4S,6aR)-2,2-dimethyl-3a,6a-dihydro-4H-cyclopenta [d] [1,3] dioxol-4-yl)-6-fluoro pyridine (3g). The corresponding compound was prepared following general procedure **A** using (2-fluoropyridin-3-yl) boronic acid. The mixture was stirred at 60 °C for 18 hours. Purification by flash chromatography (10% Et_2O/petrol to 25% Et_2O/petrol) afforded compound **3g** as a colourless oil (35% yield, 85% ee) as a single diastereomer (dr >20:1).

^{1}H NMR ($CDCl_3$, 400 MHz): δ (ppm) 8.04 (dt, *J* = 4.8, 1.6 Hz, 1H), 7.39 (ddd, *J* = 9.5, 7.2, 1.9 Hz, 1H), 7.06 (ddd, *J* = 7.2, 4.8, 1.9 Hz, 1H), 5.99 (dt, *J* = 5.8, 2.0 Hz, 1H), 5.79 – 5.77 (m, 1H), 5.25 – 5.23 (m, 1H), 4.52 (dd, *J* = 5.7, 1.1 Hz, 1H), 4.12 (d, *J* = 2.0 Hz, 1H), 1.41 (s, 3H), 1.29 (s, 3H). **^{13}C NMR** (101 MHz, $CDCl_3$): δ (ppm) 161.63 (d, *J* = 239.6 Hz), 146.09 (d, *J* = 14.7 Hz), 133.45, 132.96, 139.33 (d, *J* = 5.5 Hz), 123.41, 121.54 (d, *J* = 4.4 Hz), 110.84, 85.30, 84.35, 51.64 (d, *J* = 2.6 Hz), 27.45, 25.74. **^{19}F NMR** (376 MHz, $CDCl_3$) δ –70.4 (d, *J* = 9.7 Hz).

HRMS (ESI): m/z calcd for $C_{13}H_{15}O_2NF^+$ $[M + H]^+$ 236.1081 found 236.1081.

IR (ν_{max}/cm^{-1}) 2981, 2889, 2360, 2341, 1583, 1560, 1437, 1411, 1380, 1252, 1210, 1157, 1136, 1072, 1051, 1012, 989, 956, 865, 800, 753, 712, 692.

SFC Conditions: Chiralpak IF; 1500 PSI, 30 °C; flow: 1.5 mL/min; from 1% to 30% MeOH in 5 min; 92.5:7.5 er (major enantiomer t_R = 2.21 min; minor enantiomer t_R = 2.63 min), **85% ee**. **$[\alpha]^{25}_D$** = –126.5 (c = 1.0, $CHCl_3$).

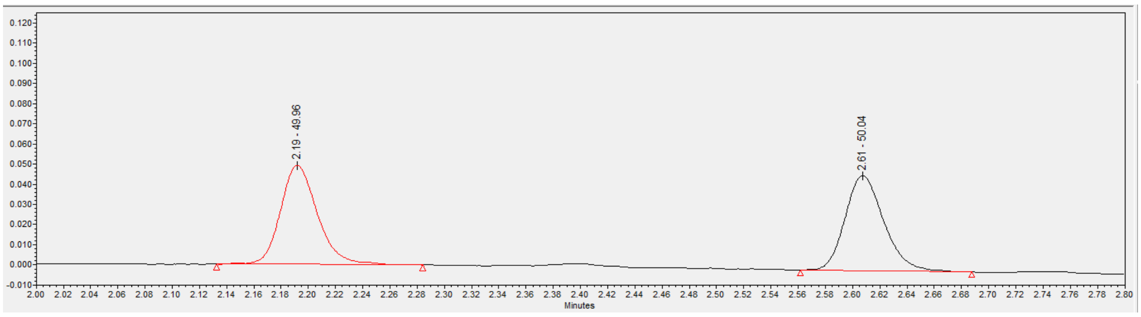

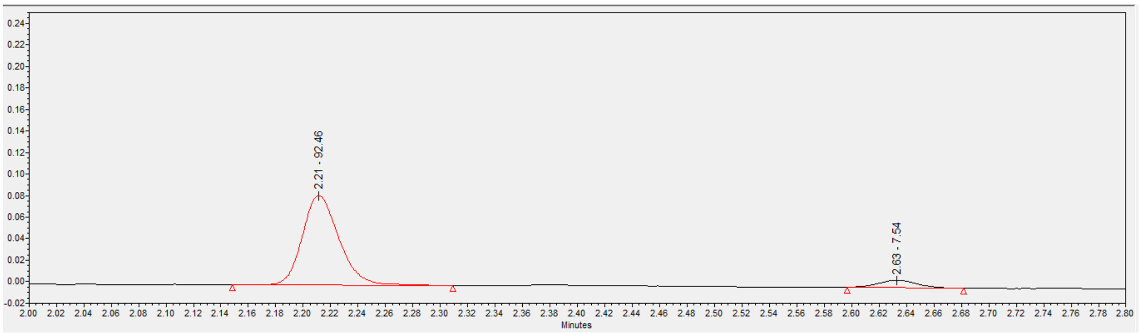

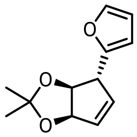

(3aS,4R,6aR)-4-(furan-2-yl)-2,2-dimethyl-3a,6a-dihydro-4H-cyclopenta [d] [1,3] dioxole (3h). The corresponding compound was prepared following general procedure **A** using 2-furanylboronic acid. The mixture was stirred at 60 °C for 18 hours. Purification by flash chromatography (5% Et_2O/petrol to 10% Et_2O/petrol) afforded compound **3h** as a colourless oil (80% yield, 97% ee) as a single diastereomer (dr >20:1).

^{1}H NMR ($CDCl_3$, 400 MHz): δ (ppm) 7.33 (dd, *J* = 1.9, 0.9 Hz, 1H, 6.28 (dd, *J* = 3.2, 1.9 Hz, 1H), 6.00 (dt, *J* = 3.2, 0.9 Hz, 1H), 5.97 (dt, *J* = 5.7, 1.9 Hz, 1H), 5.89 (ddt, *J* = 5.7, 2.4, 0.9 Hz, 1H), 5.27 (dq, *J* = 5.9, 1.5 Hz, 1H), 4.68 (d, *J* = 5.7, 1H), 4.08 (dq, *J* = 2.4, 1.3 Hz, 1H), 1.46 (s, 3H), 1.35 (s, 3H). **^{13}C NMR** (101 MHz, $CDCl_3$): δ (ppm) 154.6, 142.0, 132.9, 132.6, 110.8, 110.4, 105.6, 85.3, 83.2, 51.3, 27.6, 25.9.

IR (ν_{max}/cm^{-1}) 2983, 2935, 2360, 2342, 1506, 1458, 1371, 1333, 1282, 1251, 1211, 1159, 1107, 1070, 1050, 1014, 952, 920, 871, 806, 789.

SFC Conditions: Chiralpak IG; 1500 PSI, 30 °C; flow: 1.5 mL/min; from 1% to 30% MeOH in 5 min; 98.5:1.5 er (major enantiomer t_R = 1.71 min; minor enantiomer t_R = 2.13 min), **97% ee.** **$[\alpha]^{25}_D$** = –214.8 (c = 1.0, $CHCl_3$).

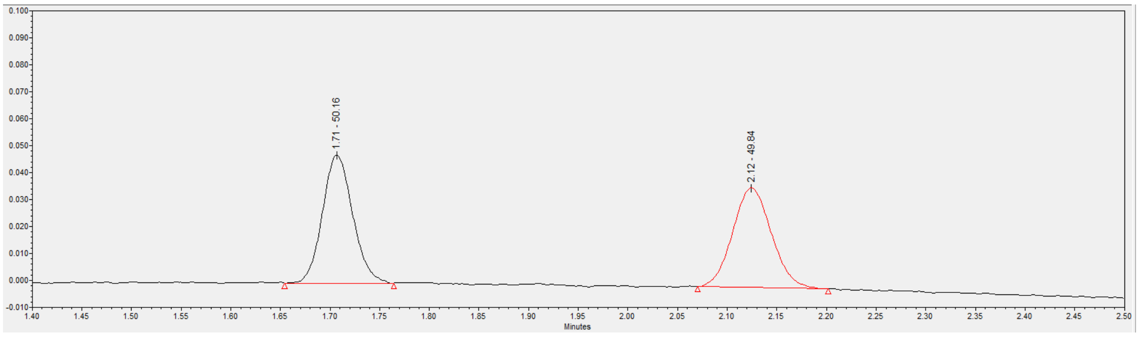

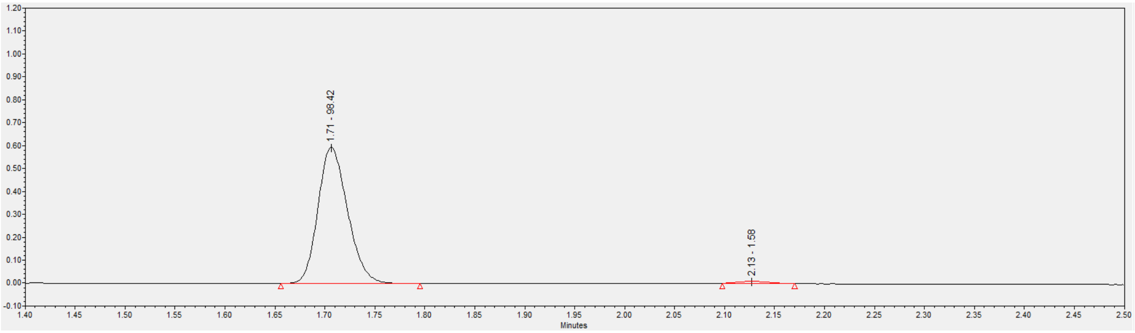

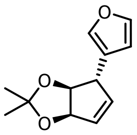

(3a*S*,4*S*,6a*R*)-4-(furan-3-yl)-2,2-dimethyl-3a,6a-dihydro-4H-cyclopenta [d] [1,3] dioxole (3i). The corresponding compound was prepared following general procedure **A** using furan-3-ylboronic acid. The mixture was stirred at 60 °C for 18 hours. Purification by flash chromatography (5% Et_2O/petrol to 10% Et_2O/petrol) afforded compound **3i** as a colourless oil (81% yield, 97% ee) as a single diastereomer (dr >20:1).

^{1}H NMR ($CDCl_3$, 400 MHz): δ (ppm) 7.37 (t, *J* = 1.7 Hz, 1H), 7.18 (dt, *J* = 1.7, 0.9 Hz, 1H), 6.24 (dd, *J* = 1.9, 0.9 Hz, 1H), 6.10 – 5.70 (m, 2H), 5.25 (dq, *J* = 5.7, 1.3 Hz, 1H), 4.52 (dq, *J* = 5.7, 0.8 Hz, 1H), 3.89 (s, 1H), 1.45 (s, 3H), 1.34 (s, 3H). The spectroscopic data satisfactorily matched previously reported data.[1]

SFC Conditions: Chiralpak IF; 1500 PSI, 30 °C; flow: 1.5 mL/min; from 1% to 30% MeOH in 5 min; 98.5:1.5 er (major enantiomer t_R = 1.42 min; minor enantiomer t_R = 1.51 min), **97% ee**. **$[\alpha]^{25}_D$** = –170.7 (c = 1.0, $CHCl_3$).

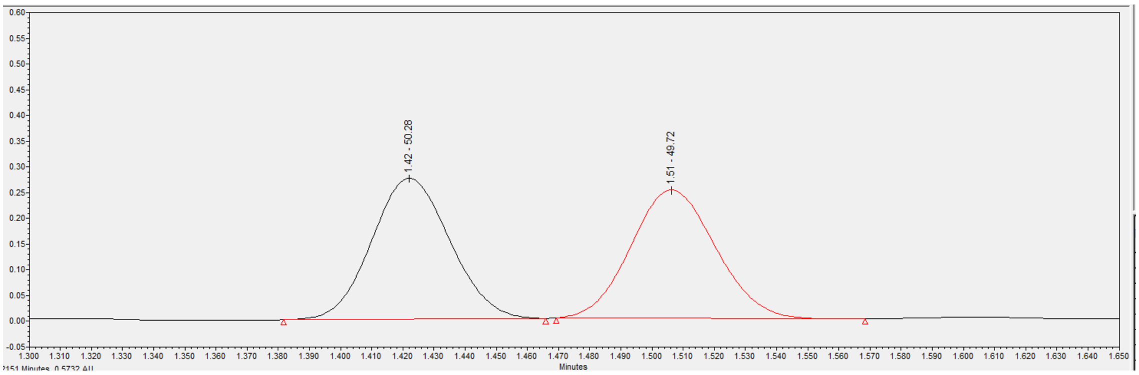

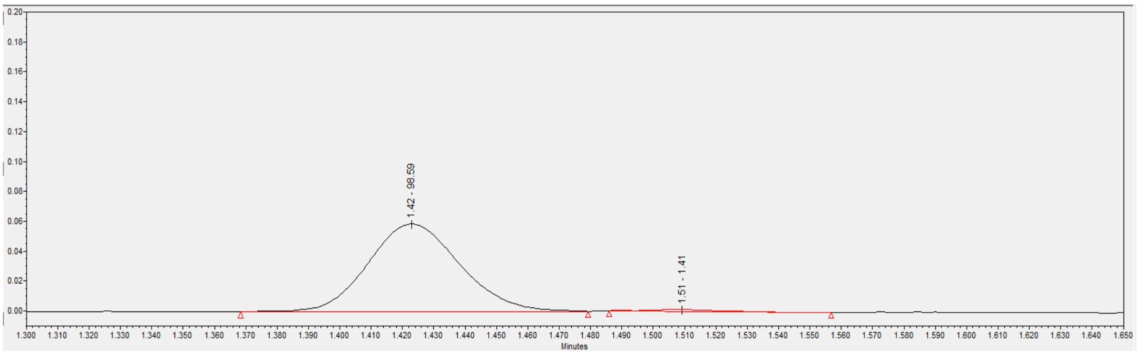

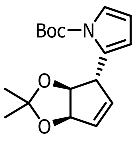

2-((3aS,4S,6aR)-2,2-dimethyl-3a,6a-dihydro-4H-cyclopenta [d] [1,3] dioxol-4-yl)-1-(tert-butoxycarbonyl)-1H-pyrrole (3j). The corresponding compound was prepared following general procedure **A** using (1-(tert-butoxycarbonyl)-1H-pyrrol-2-yl) boronic acid. The mixture was stirred at 60 °C for 18 hours. Purification by flash chromatography (5% Et_2O/petrol to 20% Et_2O/petrol) afforded compound **3j** as a colourless oil (55% yield, 97% ee) as a single diastereomer (dr >20:1).

^{1}H NMR ($CDCl_3$, 400 MHz): δ (ppm) 7.27 (ddd, J = 3.3, 1.9, 0.5 Hz, 1H), 6.04 (t, J = 3.3 Hz, 1H), 5.97 – 5.88 (m, 2H), 5.82 (ddd, J = 3.3, 1.9, 1.0 Hz, 1H), 5.18 (dq, J = 5.4, 0.9 Hz, 1H), 4.61 (s, 1H), 4.54 (dq, J = 5.4, 0.7 Hz, 1H), 1.63 (s, 9H), 1.45 (s, 3H), 1.33 (s, 3H). **^{13}C NMR** (101 MHz, $CDCl_3$): δ (ppm) 149.6, 134.7, 133.8, 132.7, 122.3, 111.5, 110.4, 109.9, 85.0, 84.4, 83.9, 50.5, 28.2, 27.9, 26.3.

HRMS (ESI): m/z calcd for $C_{17}H_{23}O_4N_1Na^+$ $[M + Na]^+$ 328.1519 found 328.1519.

IR (ν_{max}/cm^{-1}) 2981, 2936, 2361, 2341, 1738, 1488, 1458, 1395, 1371, 1255, 1213, 1075, 1052, 952, 899, 872, 848, 801, 1050, 1014, 952, 920, 871, 806, 789.

SFC Conditions: Chiralpak IC; 1500 PSI, 30 °C; flow: 1.5 mL/min; from 1% to 30% MeOH in 5 min; 98.5:1.5 er (major enantiomer t_R = 1.45 min; minor enantiomer t_R = 1.35 min), **97% ee**. $[\alpha]^{25}_D$ = –29.0 (c = 1.0, $CHCl_3$).

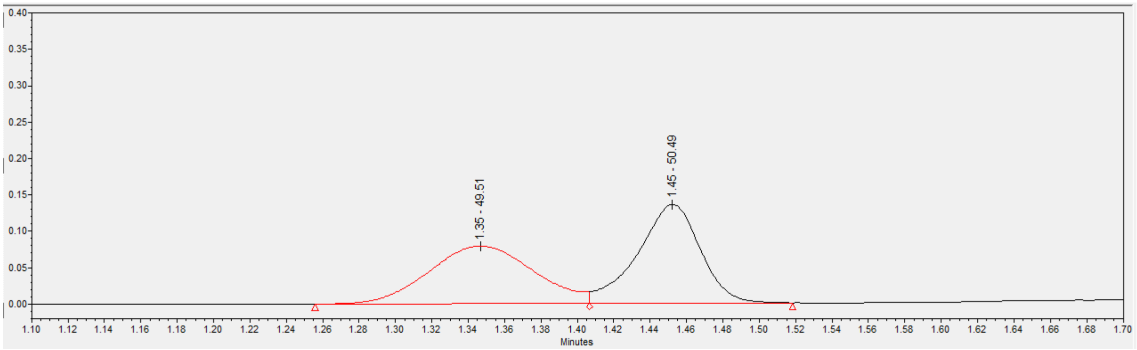

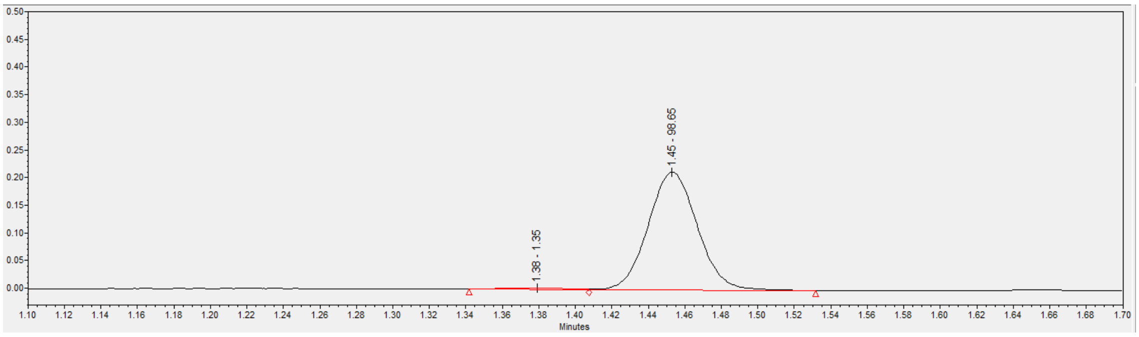

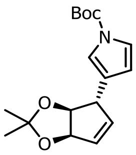

3-((3aS,4S,6aR)-2,2-dimethyl-3a,6a-dihydro-4H-cyclopenta [d] [1,3] dioxol-4-yl)-1-(tert-butoxycarbonyl)-1H-pyrrole (3k). The corresponding compound was prepared following general procedure **A** using (1-(tert-butoxycarbonyl)-1H-pyrrol-3-yl) boronic acid pinacol ester. The mixture was stirred at 60 °C for 18 hours. Purification by flash chromatography (5% Et_2O/petrol to 20% Et_2O/petrol) afforded compound **3k** as a colourless oil (65% yield, 94% ee) as a single diastereomer (dr >20:1).

^{1}H NMR ($CDCl_3$, 400 MHz): δ (ppm) 7.17 (dd, *J* = 3.3, 2.2 Hz, 1H), 6.94 – 6.93 (m, 1H), 6.06 (dd, *J* = 3.3, 1.7 Hz, 1H), 5.92 – 5.88 (m, 2H), 5.24 (dd, *J* = 5.6, 1.9 Hz, 1H), 4.50 (dd, *J* = 5.6, 0.8 Hz, 1H), 3.88 (s, 1H), 1.57 (s, 9H), 1.45 (s, 3H), 1.34 (s, 3Hz). **^{13}C NMR** (101 MHz, $CDCl_3$): δ (ppm) 148.9, 135.3, 131.4, 127.8, 120.8, 116.7, 111.9, 110.5, 85.4, 85.3, 83.7, 50.0, 28.1, 27.6, 25.9.

HRMS (ESI): m/z calcd for $C_{17}H_{23}O_4NNa^+$ $[M + Na]^+$ 328.1519 found 328.1519.

IR (ν_{max}/cm^{-1}) 2981, 2890, 2361, 2341, 1742, 1486, 1459, 1371, 1352, 1324, 1285, 1254, 1216, 1074, 1052, 972, 870, 798, 772.

SFC Conditions: Chiralpak IF; 1500 PSI, 30 °C; flow: 1.5 mL/min; from 1% to 30% MeOH in 5 min; 97:3 er (major enantiomer t_R = 2.07 min; minor enantiomer t_R = 3.10 min), **94% ee**. **$[\alpha]^{25}_D$** = –103.1 (c = 1.0, $CHCl_3$).

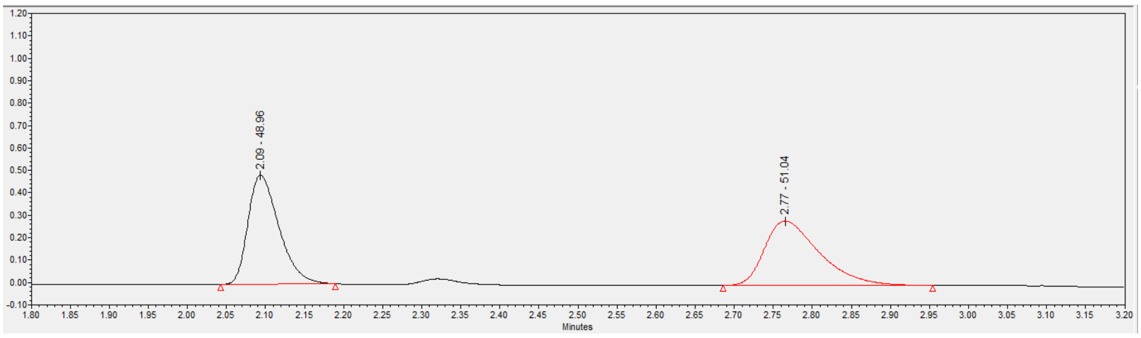

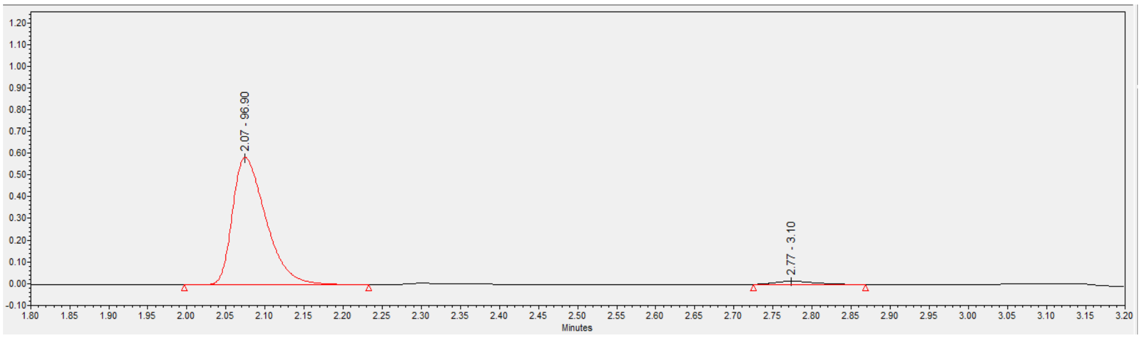

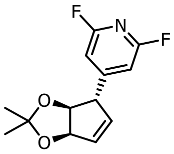

4-((3aS,4S,6aR)-2,2-dimethyl-3a,6a-dihydro-4H-cyclopenta [d] [1,3] dioxol-4-yl)-2,6-difluoropyridine (3l). The corresponding compound was prepared following general procedure **A** using (2,6-difluoropyridin-4-yl) boronic acid. The mixture was stirred at rt for 24 hours. Purification by flash chromatography (10% Et_2O/petrol to 20% Et_2O/petrol) afforded compound **3l** as a colourless oil (80% yield, 80% ee) as a single diastereomer (dr >20:1).

^{1}H NMR ($CDCl_3$, 400 MHz): δ (ppm) 6.6 (s, 2H), 6.12 (dt, *J* = 5.8, 1.9 Hz, 1H), 5.88 (ddd, *J* = 5.8, 2.1, 1.1 Hz, 1H), 5.29 (dq, *J* = 5.8, 1.4 Hz, 1H), 4.51 (dd, *J* = 5.8, 1.1 Hz, 1H), 4.07 (s, 1H), 1.47 (s, 3H), 1.35 (s, 3H). **^{13}C NMR** (101 MHz, $CDCl_3$): δ (ppm) 162.4 (d, *J* = 247.4 Hz), 162.2 (d, *J* = 247.2 Hz), 161.4 (d, *J* = 7.2 Hz), 134.4, 133.2, 111.4, 111.0 (d, *J* = 37.7 Hz),

105.1 (d, J = 39.9 Hz), 85.14, 84.08, 57.5 (d, J = 3.3 Hz), 25.8, 25.0. **^{19}F NMR** (376 MHz, $CDCl_3$) δ –68.2.

HRMS (ESI): m/z calcd for $C_{13}H_{14}O_2NF_2^+$ $[M + H]^+$ 254.0987 found 254.0986.

IR (ν_{max}/cm^{-1}) 2986, 2936, 2361, 1625, 1569, 1552, 1473, 1423, 1404, 1382, 1351, 1245, 1213, 1189, 1158, 1143, 1110, 1078, 1052, 1031, 1021, 984, 966, 908, 866, 849, 777, 759.

SFC Conditions: Chiralpak IF; 1500 PSI, 30 °C; flow: 1.5 mL/min; from 1% to 30% MeOH in 5 min; 90:10 er (major enantiomer t_R = 1.40 min; minor enantiomer t_R = 1.79 min), **80% ee.** **[α]^{25}$_D$** = –161.3 (c = 1.0, $CHCl_3$).

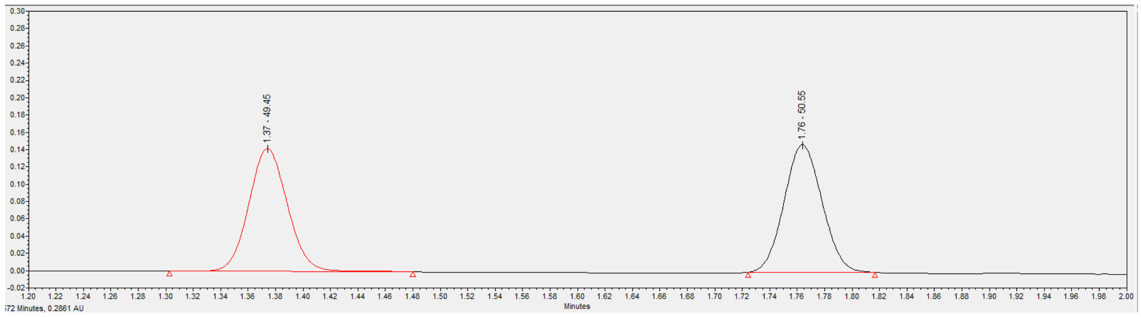

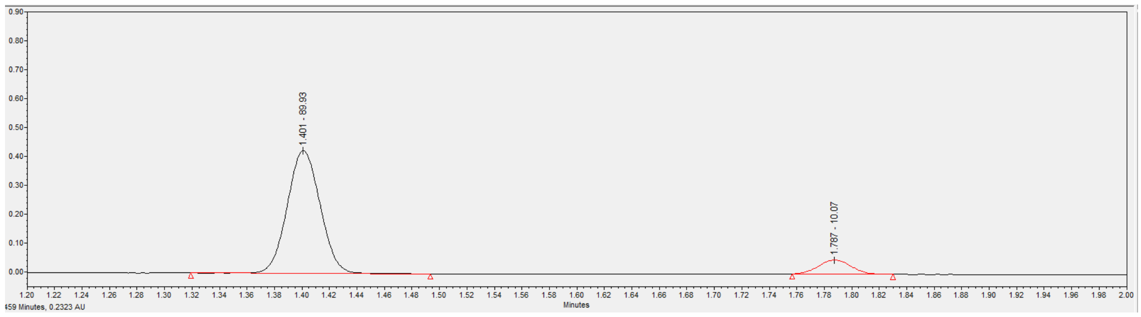

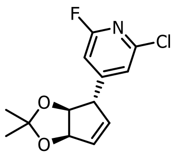

2-chloro-4-((3a*S*,4*S*,6a*R*)-2,2-dimethyl-3a,6a-dihydro-4H-cyclopenta [d] [1,3] dioxol-4-yl)-6-fluoropyridine (3m). The corresponding compound was prepared following general procedure **A** using 3-chloro-5-fluoro-4-pyridylboronic acid. The mixture was stirred at rt for 24 hours. Purification by flash chromatography (15% Et_2O/petrol to 30% Et_2O/petrol) afforded compound **3m** as a colourless oil (92% yield, 80% ee) as a single diastereomer (dr >20:1).

^{1}H NMR ($CDCl_3$, 400 MHz): δ (ppm) 7.01 (t, *J* = 1.2 Hz, 1H), 6.64 (t, *J* = 1.5 Hz, 1H), 6.1j2 (dt, *J* = 5.8, 1.9 Hz, 1H), 5.86 (ddd, *J* = 5.7, 2.3, 1.1 Hz, 1H), 5.29 (dq, *J* = 5.8, 1.5 Hz, 1H), 4.49 (dt, *J* = 5.7, 1.1 Hz, 1H), 4.03 (s, 1H), 1.47 (s, 3H), 1.34 (s, 3H). **^{13}C NMR** (101 MHz, $CDCl_3$): δ (ppm) 162.9, (d, *J* = 246.0 Hz),159.2 (d, *J* = 7.2 Hz), 149.5 (d, *J* = 15.1 Hz), 133.8 (d, *J* = 138.3 Hz), 120.8 (d, *J* = 4.7 Hz), 111.4, 106.9 (d, *J* = 35.4 Hz), 85.1, 85.0, 57.4 (d, *J* = 2.8 Hz), 27.5, 25.8. **^{19}F NMR** (377 MHz, $CDCl_3$): δ (ppm) –66.3.

HRMS (ESI): m/z calcd for $C_{13}H_{14}O_2NClF$ $[M + H]^+$ 270.0692 found 290.0692.

IR (ν_{max}/cm^{-1}) 2984, 2930, 2361, 2342, 1741, 1603, 1558, 1458, 1437, 1406, 1380, 1301, 1250, 1211, 1174, 1159, 1077, 1052, 985, 908, 855, 806, 753.

SFC Conditions: Chiralpak IC; 1500 PSI, 30 °C; flow: 1.5 mL/min; from 1% to 30% MeOH in 5 min; 90:10 er (major enantiomer t_R = 1.79 min; minor enantiomer t_R = 2.32 min), **80% ee**. **$[\alpha]^{25}_{D}$** = –93.8 (c = 1.0, $CHCl_3$).

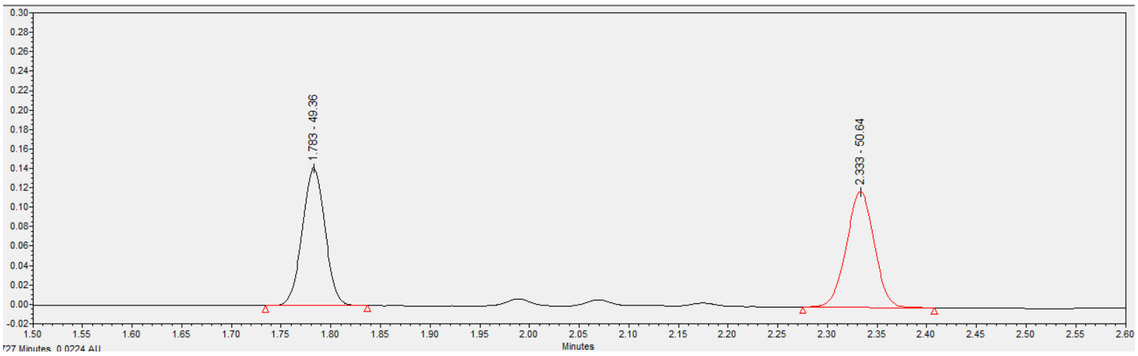

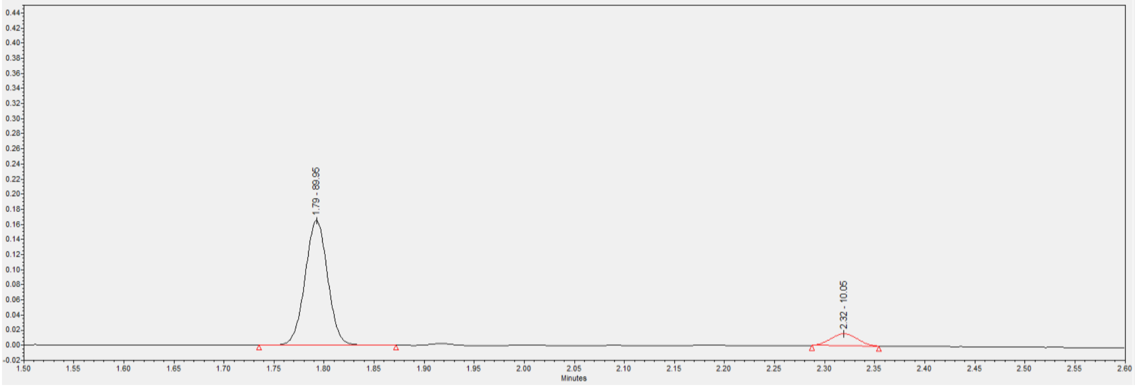

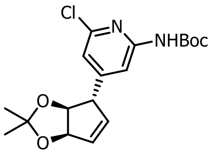

6-chloro-4-((3aS,4S,6aR)-2,2-dimethyl-3a,6a-dihydro-4H-cyclopenta [d] [1,3] dioxol-4-yl) pyridin-2-tert-butyl carbamate (3n). The corresponding compound was prepared following general procedure **A** using (2-tert-butyl carbamate-6-chloropyridin-4-yl) boronic acid. The mixture was stirred at 60 °C for 18 hours. Purification by flash chromatography (10% Et_2O/petrol to 25% Et_2O/petrol) afforded compound **3n** as a colourless oil (79% yield, 80% ee) as a single diastereomer (dr >20:1).

^{1}H NMR ($CDCl_3$, 400 MHz): δ (ppm) 7.67 (d, *J* = 1.2 Hz, 1H), 6.73 (d, *J* = 1.2 Hz, 1H), 6.08 (dt, *J* = 5.7, 2.1 Hz, 1H), 5.87 (ddd, *J* = 5.7, 2.1, 1.3 Hz, 1H), 5.30 (dq, *J* = 5.7, 1.3 Hz, 1H), 4.51 (d, *J* = 5.7 Hz, 1H), 3.99 (s, 1H), 1.51 (s, 9H), 1.46 (s, 3H), 1.33 (s, 3H). **^{13}C NMR** (101 MHz, $CDCl_3$): δ (ppm) 156.4, 152.2, 152.0, 149.5, 133.9, 133.8, 117.5, 111.1, 109.4, 85.3, 85.2, 81.7, 57.7, 28.3, 27.5, 25.8.

HRMS (ESI): m/z calcd for $C_{18}H_{24}O_4N_2Cl^+$ [M + H]$^+$ 367.1419 found 367.1421.

IR (ν_{max}/cm^{-1}) 3280, 2981, 2890, 2360, 2341, 1732, 1602, 1561, 1524, 1424, 1393, 1370, 1278, 1224, 1075, 1053, 955, 863, 802, 763.

SFC Conditions: Chiralpak IC; 1500 PSI, 30 °C; flow: 1.5 mL/min; from 1% to 30% MeOH in 5 min; 90:10 er (major enantiomer t_R = 2.20 min; minor enantiomer t_R = 2.30 min), 80% ee. **[α]$^{25}_{D}$** = –136.3 (c = 1.0, $CHCl_3$).

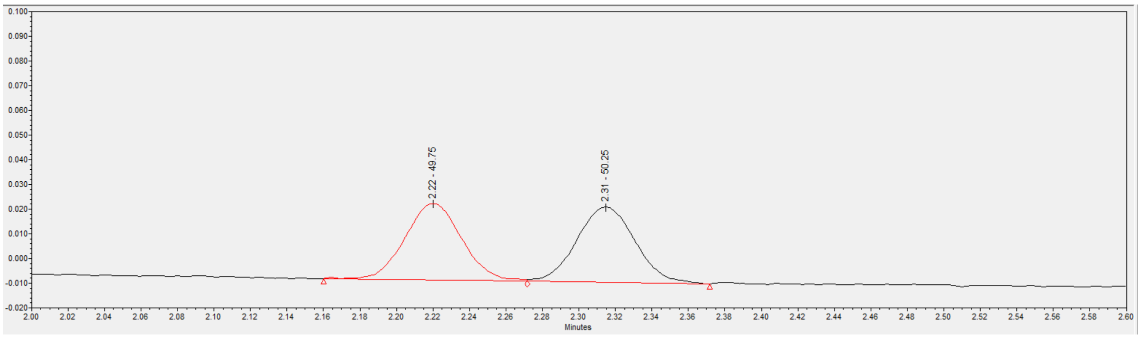

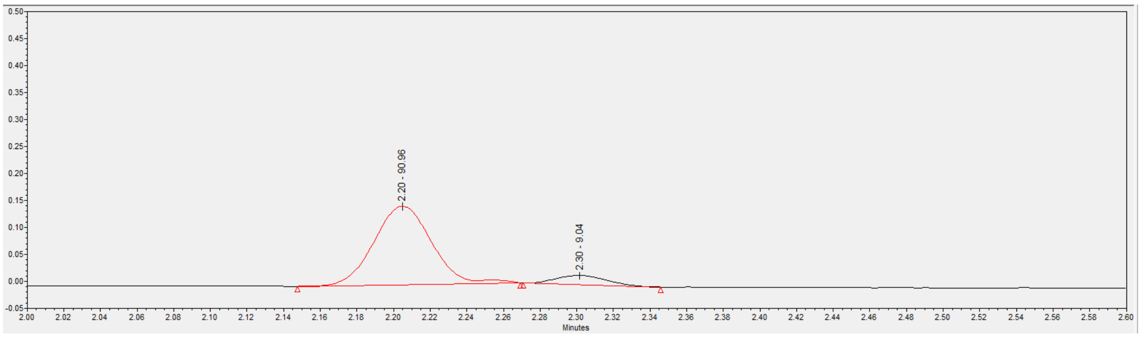

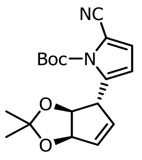

2-cyano-5-((3aS,4S,6aR)-2,2-dimethyl-3a,6a-dihydro-4H-cyclopenta [d] [1,3] dioxol-4-yl)-1-(tert-butoxycarbonyl)-1H-pyrrole (3o). The corresponding compound was prepared following general procedure **A** using (1-(tert-butoxycarbonyl)-5-cyano-1H-pyrrol-2-yl) boronic acid. The mixture was stirred at 60 °C for 18 hours. Partial deprotection of Boc group was observed under the reaction condition providing 2:1 mixture of desired product and Boc-deprotected product. The mixture was subjected to Boc protection reaction conditions then purified by flash chromatography (10% Et_2O/petrol to 25% Et_2O/petrol) affording compound **3o** as a colourless oil (58% yield, 94% ee) as a single diastereomer (dr >20:1).

^{1}H NMR ($CDCl_3$, 400 MHz): δ (ppm) 6.81 (dd, *J* = 3.8, 0.4 Hz, 1H), 5.99 (dt, *J* = 5.8, 1.8 Hz, 1H), 5.92 – 5.84 (m, 2H), 5.17 (dd, *J* = 5.4, 1.4 Hz, 1H), 4.59 (s, 1H), 4.52 (dd, *J* = 5.4, 0.8 Hz, 1H), 1.69 (s, 9H), 1.44 (s, 3H), 1.33 (s, 3H).**^{13}C NMR** (101 MHz, $CDCl_3$): δ (ppm) 147.6, 141.0, 133.9, 132.7, 124.3, 113.5, 111.5, 110.8, 105.4, 87.9, 84.8, 83.7, 50.8, 27.9, 27.8, 26.2.

HRMS (ESI): m/z calcd for $C_{18}H_{22}O_4N_2Na^+$ $[M + Na]^+$ 353.1472 found 353.1472.

SFC Conditions: Chiralpak IG; 1500 PSI, 30 °C; flow: 1.5 mL/min; from 1% to 30% MeOH in 5 min; 97:3 er (major enantiomer t_R = 1.8 min; minor enantiomer t_R = 2.08 min), **94% ee**.

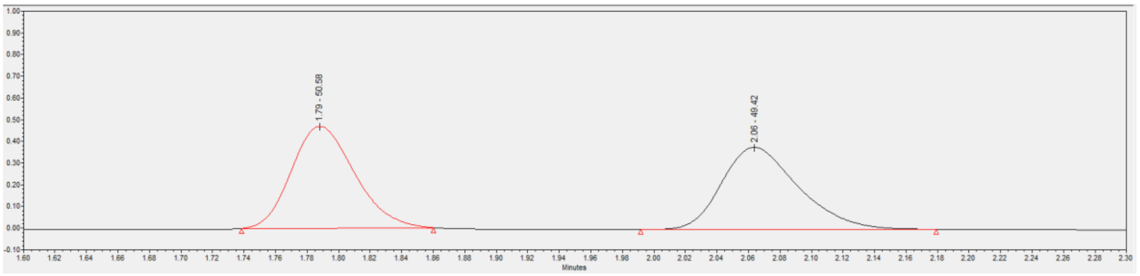

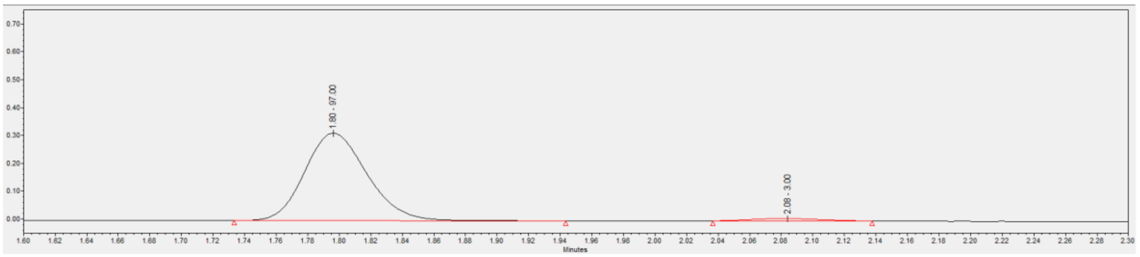

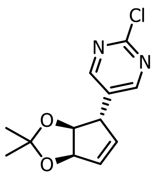

2-chloro-5-((3aS,4S,6aR)-2,2-dimethyl-3a,6a-dihydro-4H-cyclopenta [d] [1,3] dioxol-4-yl) pyrimidine (3p). The corresponding compound was prepared following general procedure **A** using (2-chloropyrimidin-5-yl) boronic acid with 5 mol% of Rh dimer and 12 mol% of (*S*)-Segphos. The mixture was stirred at 60 °C for 18 hours. Purification by flash chromatography (10% Et_2O/petrol to 25% Et_2O/petrol) afforded compound **3p** as a colourless oil (67% yield, 94% ee) as a single diastereomer (dr >20:1).

^{1}H NMR ($CDCl_3$, 400 MHz): δ (ppm) 8.40 (s, 2H), 6.12 (dt, *J* = 5.8, 1.9 Hz, 1H), 5.88 (ddt, *J* = 5.8, 2.4, 1.1 Hz, 1H), 5.32 (dq, *J* = 5.7, 1.4 Hz, 1H), 4.49 (dt, *J* = 5.7, 1.1 Hz, 1H), 4.03 (dt, *J* = 2.4, 1.4 Hz, 1H), 1.47 (s, 2H), 1.34 (s, 2H). **^{13}C NMR** (101 MHz, $CDCl_3$): δ (ppm) 160.4, 158.8, 134.3, 133.6, 133.0, 111.5, 85.3, 85.2, 52.8, 27.5, 25.8.

HRMS (ESI): m/z calcd for $C_{12}H_{14}O_2N_2Cl^+$ $[M + H]^+$ 253.0738 found 253.0726.

IR (ν_{max}/cm^{-1}) 2981, 2889, 2361, 2342, 1577, 1548, 1461, 1393, 1382, 1371, 1255, 1235, 1217, 1106, 1056, 1028, 953, 895, 883, 865, 839, 802, 771, 759, 715.

SFC Conditions: Chiralpak IC; 1500 PSI, 30 °C; flow: 1.5 mL/min; from 1% to 30% MeOH in 5 min; 97:3 er (major enantiomer t_R = 2.85 min; minor enantiomer t_R = 3.73 min), **94% ee**. **$[\alpha]^{25}_D$** = –217.6 (c = 1.0, $CHCl_3$).

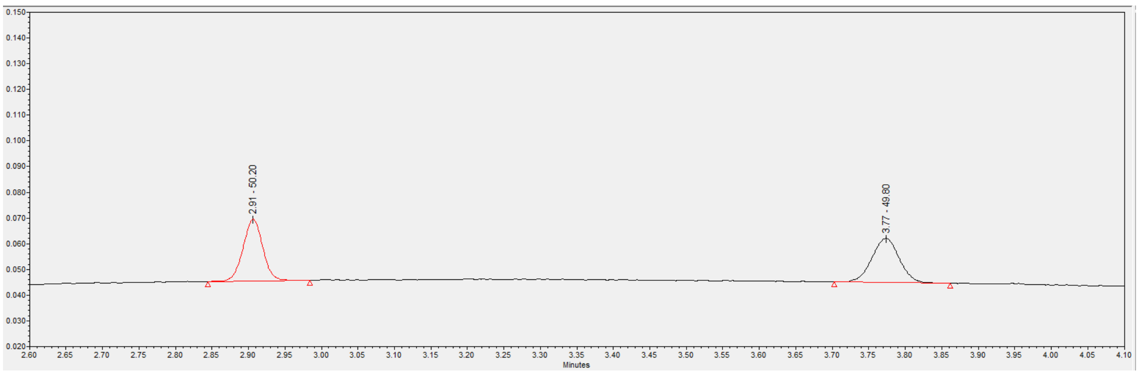

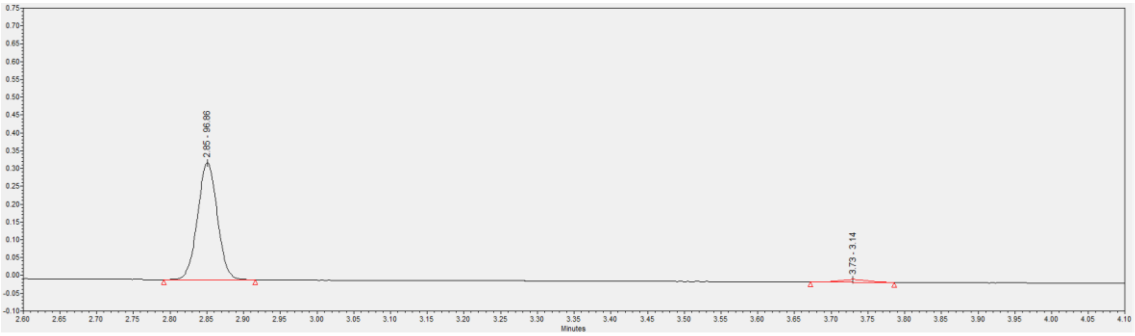

2.85 - 96.86
3.73 - 3.14
Minutes

3. Synthesis of Carbocyclic C-Nucleosides (CC-Ns)

(1S,2R,3R,5S)-3-(hydroxymethyl)-5-phenylcyclopentane-1,2-diol (7a):

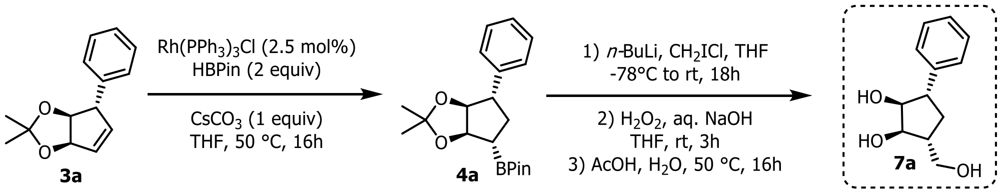

According to modified procedure by Srebnik et. al,[3] [Rh(PPh_3)$_3$Cl] (23.1 mg, 0.025 mmol, 2.5 mol%) and Cs_2CO_3 (325.8 mg, 1.0 mmol, 1 equiv) were added to a flame dried 25 mL round bottom flask, sealed with a rubber septum under an argon atmosphere, dissolved in THF (0.5 mL) and stirred at room temperature. After 5 min, a solution (or suspension) of **3a** (1 mmol, 1.0 equiv) in THF (3 mL) was added via syringe and the flask was rinsed with THF (0.5 mL). Pinacolborane (0.29 mL, 2 mmol, 2.0 equiv) was added dropwise to the reaction mixture at 50 °C and stirred for 16 hours at 50 °C. The mixture was then cooled to room temperature and diluted with Et_2O (10 mL) before passing through a plug of celite. The plug was washed with an additional 15 mL of Et_2O and the solvents were removed in vacuo. Purification by flash chromatography (10% Et_2O/petrol to 25% Et_2O/petrol) afforded compound **4a** as a colourless oil (83% yield) as a single diastereomer (dr >20:1).

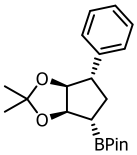

((3aR,4S,6S,6aS)-2,2-dimethyl-6-phenyltetrahydro-4H-cyclopenta[d][1,3]dioxol-4-yl) boronic acid pinacol ester (4a): ^{1}**H NMR** ($CDCl_3$, 400 MHz): δ (ppm) 7.35 – 7.25 (m, 4H), 7.25 – 7.19 (m, 1H), 4.83 (dd, *J* = 6.8, 5.3 Hz, 1H), 4.61 (t, *J* = 6.3 Hz, 1H), 3.23 (dt, *J* = 10.7, 6.3 Hz, 1H), 2.43 (dt, *J* = 12.7, 7.3 Hz, 1H), 1.83 (dt, *J* = 12.7, 10.7 Hz, 1H), 1.69 (ddd, *J* = 11.5, 7.3, 5.3 Hz, 1H), 1.58 (s, 3H), 1.36 (s, 3H), 1.26 (s, 6H), 1.23 (s, 6H). ^{13}**C NMR** (101 MHz, $CDCl_3$): δ (ppm) 142.7, 128.5, 127.4, 126.4, 112.7, 87.8, 83.6, 83.1, 52.2, 34.8, 27.9, 25.4, 25.0, 24.9.

In a flamed dried flask under nitrogen, *n*-butyllithium (2.5 M in hexanes, 0.75 mL, 1.88 mmol, 2.5 equiv) was added dropwise to a solution of pinacol boronic ester **4a** (0.75 mmol, 1.0 equiv) and iodochloromethane (165 µL, 2.25 mmol, 3 equiv) in THF (3.5 mL) at -78 °C. The reaction mixture was slowly allowed to warm to ambient temperature over 12 h. The mixture was diluted with Et_2O (5 mL) and the organic layer was washed with an aq. sat. solution of NH_4Cl (2 x 3

mL) and dried over Na_2SO_4, filtered, and the solvent was removed in vacuo. The crude product boronic acid pinacol ester **5a** was charged to the next step.

30%wt. H_2O_2 (1 mL) was added to a solution of boronic acid pinacol ester **5a** obtained above (0.75 mmol, 1.0 equiv) and 2N aq. NaOH (1.5 ml) in THF (4 mL) at 0 °C. The reaction mixture was slowly allowed to warm to ambient temperature. After 3h, the mixture was diluted with Et_2O (7 mL). The organic layer was separated and the aqueous layer was extracted with Et_2O (2 × 5 mL). The combined organic layers were dried over anhydrous Na_2SO_4, filtered, and the solvent was removed in vacuo. The crude alcohol **6a** was charged to the next step.

A solution of **7a** obtained above (0.2 mmol) in AcOH (0.5 mL) and H_2O (0.5 mL) was stirred at 50 °C for 16 h. The reaction mixture was concentrated under reduced pressure and purification by flash chromatography (1% MeOH/EtOAc to 10% MeOH/EtOAc) afforded compound **8a** as a colourless oil (77% yield over 3 steps) as a single diastereomer (dr >20:1).

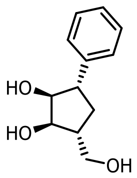

(1S,2R,3R,5S)-3-(hydroxymethyl)-5-phenylcyclopentane-1,2-diol (7a): ^{1}H NMR (DMSO-*d*$_6$, 400 MHz): δ (ppm) 7.35 – 7.22 (m, 4H), 7.21 – 7.11 (m, 1H), 3.73 – 3.69 (m, 2H), 3.46 – 3.37 (m, 2H), 3.01 – 2.94 (m, 1H), 2.07 – 1.93 (m, 2H), 1.32 – 1.23 (m, 1H). The spectroscopic data satisfactorily matched previously reported data.[4] Decomposition peaks observed in DMSO-*d*$_6$. Full data collected in Methanol-d$_4$.

^{1}H NMR (CD_3OD, 400 MHz): δ (ppm) 7.33 – 7.25 (m, 5H), 7.20 – 7.14 (m, 1H), 3.94 – 3.87 (m, 2H), 3.70 – 3.56 (m, 2H), 3.15 – 3.06 (m, 1H), 2.24 – 2.09 (m, 3H), 1.50 – 1.35 (m, 1H). **^{13}C NMR** (101 MHz, CD_3OD): δ (ppm) 144.6, 129.4, 128.6, 127.2, 79.9, 75.5, 64.9, 50.9, 48.2, 33.2. **$[\alpha]^{25}_D$** = –51.4 (c = 1.0, CH_3OH).

HRMS (ESI): m/z calcd for $C_{12}H_{16}O_3Na^+$ $[M + Na]^+$ 231.0992 found 231.0993.

IR (ν_{max}/cm^{-1}) 3348, 3029, 2919, 2865, 2360, 2341, 1602, 1494, 1455, 1425, 1375, 1338, 1275, 1155, 1122, 1078, 1056, 995, 967, 914, 896, 812, 757, 731, 771, 759, 715.

(1R,2S,3S,5R)-3-(6-chloropyridin-3-yl)-5-(hydroxymethyl)cyclopentane-1,2-diol (7b):

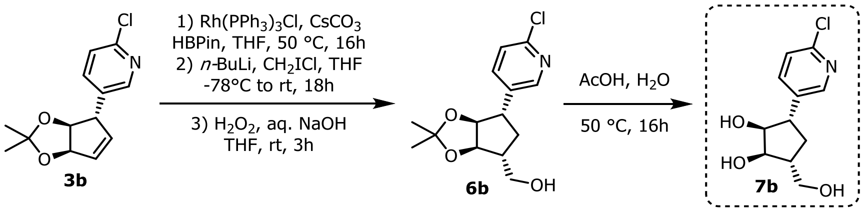

According to modified procedure by Srebnik et. al,[3] [Rh(PPh$_3$)$_3$Cl] (23.1 mg, 0.025 mmol, 2.5 mol%) and Cs_2CO_3 (325.8 mg, 1.0 mmol, 1 equiv) were added to a flame dried 25 mL round bottom flask, sealed with a rubber septum under an argon atmosphere, dissolved in THF (0.5 mL) and stirred at room temperature. After 5 min, a solution (or suspension) of **3b** (1 mmol, 1.0 equiv) in THF (3 mL) was added via syringe and the flask was rinsed with THF (0.5 mL). Pinacolborane (0.29 mL, 2 mmol, 2.0 equiv) was added dropwise to the reaction mixture at 50 °C and stirred for 16 hours at 50 °C. The mixture was then cooled to room temperature and diluted with Et_2O (5 mL) before passing through a plug of silica. The plug was washed with an additional 10 mL of Et_2O and the solvents were removed in vacuo. The crude product boronic acid pinacol ester **4b** was charged to the next step without further purification.

In a flamed dried flask under nitrogen, *n*-butyllithium (2.5 M in hexanes, 0.75 mL, 1.88 mmol, 2.5 equiv) was added dropwise to a solution of boronic acid pinacol ester **4b** obtained above (0.75 mmol, 1.0 equiv) and iodochloromethane (165 µL, 2.25 mmol, 3 equiv) in THF (3.5 mL) at -78 °C. The reaction mixture was slowly allowed to warm to ambient temperature over 12 h. The mixture was diluted with Et_2O (5 mL) and the organic layer was washed with an aq. sat. solution of NH_4Cl (2 x 3 mL) and dried over Na_2SO_4, filtered, and the solvent was removed in vacuo. The crude product boronic acid pinacol ester **5b** was charged to the next step.

30%wt. H_2O_2 (1 mL) was added to a solution of boronic acid pinacol ester **5b** obtained above (0.75 mmol, 1.0 equiv) and 2N aq. NaOH (1.5 ml) in THF (4 mL) at 0 °C. The reaction mixture was slowly allowed to warm to ambient temperature. After 3h, the mixture was diluted with Et_2O (7 mL). The organic layer was separated and the aqueous layer was extracted with Et_2O (2 × 5 mL). The combined organic layers were dried over anhydrous Na_2SO_4, filtered, and the solvent was removed in vacuo. Purification by flash chromatography (15% Et_2O/petrol to 30% Et_2O/petrol) afforded alcohol **6b** as a colourless oil (34% yield over 3 steps) as a single diastereomer (dr >20:1).

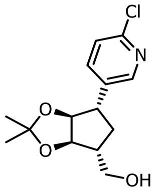

(1R,2S,3S,5R)-3-(6-chloropyridin-3-yl)-5-(hydroxymethyl)cyclopentane-1,2-diol (6b): ^{1}H NMR ($CDCl_3$, 400 MHz): δ (ppm) 8.29 (d, *J* = 2.6 Hz, 1H), 7.58 (ddd, *J* = 8.2, 2.6, 0.7 Hz, 1H), 7.28 (d, *J* = 8.2 Hz, 1H), 4.54 (dd, *J* = 7.3, 5.0 Hz, 1H), 4.45 (t, *J* = 7.1 Hz, 1H), 3.81 (dd, *J* = 10.5, 5.4 Hz, 1H), 3.73 (dd, *J* = 10.5, 6.3 Hz, 1H), 3.20 (dt, *J* = 12.8, 6.6 Hz, 1H), 2.40 (tdd, *J* = 11.5, 6.3, 5.1 Hz, 1H), 2.31 (dt, *J* = 12.9, 6.6 Hz, 1H), 1.70 (td, *J* = 12.9, 11.5 Hz, 1H), 1.56 (s, 3H), 1.32 (s, 3H). **^{13}C NMR** (101 MHz, $CDCl_3$): δ (ppm) 149.8, 148.5, 137.7, 136.7, 124.2, 113.8, 86.8, 82.9, 64.1, 60.5, 48.0, 47.3, 34.4, 27.8, 25.3, 25.0, 21.2, 14.3. **$[\alpha]^{25}_{D}$** = –51.5 (c = 1.0, $CHCl_3$).

A solution of alcohol **6b** (0.2 mmol) in AcOH (0.5 mL) and H_2O (0.5 mL) was stirred at 50 °C for 16 h. The reaction mixture was concentrated under reduced pressure and purification by flash chromatography (1% MeOH/EtOAc to 10% MeOH/EtOAc) afforded compound **7b** as a colourless oil (91% yield) as a single diastereomer (dr >20:1).

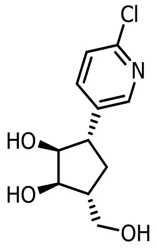

(1R,2S,3S,5R)-3-(6-chloropyridin-3-yl)-5-(hydroxymethyl)cyclopentane-1,2-diol (7b): ^{1}H NMR (CD_3OD, 400 MHz): δ (ppm) 8.32 (d, *J* = 2.5 Hz, 1H), 7.82 (dd, *J* = 8.2, 2.5 Hz, 1H), 7.42 (dd, *J* = 8.2, 0.7 Hz, 1H), 3.93 (dd, *J* = 5.6, 2.9 Hz, 1H), 3.87 (dd, *J* = 9.4, 5.6 Hz, 1H), 3.64 (d, *J* = 5.6 Hz, 2H), 3.17 (ddd, *J* = 11.8, 9.4, 7.4 Hz, 1H), 2.28 – 2.13 (m, 3H), 1.52 – 1.38 (m, 1H). **^{13}C NMR** (101 MHz, CD_3OD): δ (ppm) 150.1, 139.9, 125.3, 79.7, 75.3, 64.7, 48.3, 47.4, 32.1. **$[\alpha]^{25}_{D}$** = –57.4 (c = 1.0, CH_3OH).

HRMS (ESI): m/z calcd for $C_{11}H_{15}O_3NCl^+$ $[M + H]^+$ 244.0735 found 244.0735.

IR (ν_{max}/cm^{-1}) 3338, 2926, 2875, 2360, 2341, 1659, 1588, 1567, 1402, 1345, 1225, 1070, 1029, 901, 836, 754, 739, 681.

(1R,2S,3S,5R)-3-(6-fluoropyridin-3-yl)-5-(hydroxymethyl)cyclopentane-1,2-diol (7c):

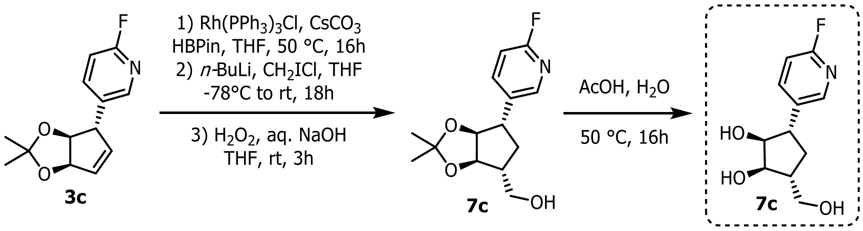

According to modified procedure by Srebnik et. al,[3] [Rh(PPh_3)$_3$Cl] (23.1 mg, 0.025 mmol, 2.5 mol%) and Cs_2CO_3 (325.8 mg, 1.0 mmol, 1 equiv) were added to a flame dried 25 mL round bottom flask, sealed with a rubber septum under an argon atmosphere, dissolved in THF (0.5 mL) and stirred at room temperature. After 5 min, a solution (or suspension) of **3c** (1 mmol, 1.0 equiv) in THF (3 mL) was added via syringe and the flask was rinsed with THF (0.5 mL). Pinacolborane (0.29 mL, 2 mmol, 2.0 equiv) was added dropwise to the reaction mixture at 50 °C and stirred for 16 hours at 50 °C. The mixture was then cooled to room temperature and diluted with Et_2O (5 mL) before passing through a plug of silica. The plug was washed with an additional 10 mL of Et_2O and the solvents were removed in vacuo. The crude product boronic acid pinacol ester **4c** was charged to the next step without further purification.

In a flamed dried flask under nitrogen, *n*-butyllithium (2.5 M in hexanes, 0.75 mL, 1.88 mmol, 2.5 equiv) was added dropwise to a solution of boronic acid pinacol ester **4c** obtained above (0.75 mmol, 1.0 equiv) and iodochloromethane (165 µL, 2.25 mmol, 3 equiv) in THF (3.5 mL) at -78 °C. The reaction mixture was slowly allowed to warm to ambient temperature over 12 h. The mixture was diluted with Et_2O (5 mL) and the organic layer was washed with an aq. sat. solution of NH_4Cl (2 x 3 mL) and dried over Na_2SO_4, filtered, and the solvent was removed in vacuo. The crude product boronic acid pinacol ester **5c** was charged to the next step without further purification.

30%wt. H_2O_2 (1 mL) was added to a solution of boronic acid pinacol ester **5c** obtained above (0.75 mmol, 1.0 equiv) and 2N aq. NaOH (1.5 ml) in THF (4 mL) at 0 °C. The reaction mixture was slowly allowed to warm to ambient temperature. After 3h, the mixture was diluted with Et_2O (7 mL). The organic layer was separated and the aqueous layer was extracted with Et_2O (2 × 5 mL). The combined organic layers were dried over anhydrous Na_2SO_4, filtered, and the solvent was removed in vacuo. Purification by flash chromatography (15% Et_2O/petrol to 30% Et_2O/petrol) afforded compound **6c** as a colourless oil (71% yield over 3 steps) as a single diastereomer (dr >20:1).

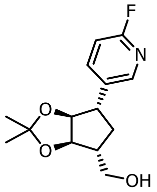

((3aR,4R,6S,6aS)-6-(6-fluoropyridin-3-yl)-2,2-dimethyltetrahydro-4H-cyclopenta [d] [1,3] dioxol-4-yl) methanol (6c): ^{1}**H NMR** ($CDCl_3$, 400 MHz): δ (ppm) 8.09 (dq, *J* = 2.6, 0.8 Hz, 1H), 7.71 (dddd, *J* = 8.4, 7.7, 2.6, 0.8 Hz, 1H), 6.91 – 6.85 (m, 1H), 4.54 (dd, *J* = 7.2, 4.9 Hz, 1H), 4.44 (t, *J* = 7.2 Hz, 1H), 3.81 (dd, *J* = 10.6, 5.4 Hz, 1H), 3.73 (dd, *J* = 10.6, 6.3 Hz, 1H), 3.20 (dt, *J* = 13.1, 6.6 Hz, 1H), 2.39 (tdd, *J* = 11.5, 6.3, 5.1 Hz, 1H), 2.30 (dt, *J* = 12.8, 6.6 Hz, 1H), 1.70 (td, *J* = 12.8, 11.5 Hz, 1H), 1.56 (s, 3H), 1.32 (s, 3H). ^{13}**C NMR** (101 MHz, $CDCl_3$): δ (ppm) 162.7 (d, *J* = 238.0 Hz), 146.0 (d, *J* = 14.2 Hz), 140.1 (d, *J* = 7.7 Hz), 135.3 (d, *J* = 4.7 Hz), 113.77, 109.4 (d, *J* = 37.3 Hz), 86.9, 82.9, 64.0, 47.8, 47.3, 34.6, 27.8, 25.3. ^{19}**F NMR** (376 MHz, $CDCl_3$): δ (ppm) –71.3 (d, *J* = 5.1 Hz). **$[\alpha]^{25}_{D}$** = –37.1 (c = 1.0, $CHCl_3$).

HRMS (ESI): m/z calcd for $C_{14}H_{19}O_3NF^+$ $[M + H]^+$ 268.1343 found 268.1342.

IR (ν_{max}/cm^{-1}) 3386, 2988, 2933, 2876, 2360, 2342, 1598, 1489, 1457, 1407, 1381, 1304, 1253, 1210, 1161, 1068, 1028, 969, 864, 833.

A solution of alcohol **6c** (0.2 mmol) in AcOH (0.5 mL) and H_2O (0.5 mL) was stirred at 50 °C for 16 h. The reaction mixture was concentrated under reduced pressure and purification by flash chromatography (1% MeOH/EtOAc to 10% MeOH/EtOAc) afforded compound **7c** as a colourless oil (88% yield) as a single diastereomer (dr >20:1).

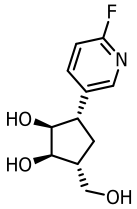

(1R,2S,3S,5R)-3-(6-fluoropyridin-3-yl)-5-(hydroxymethyl)cyclopentane-1,2-diol (7c): ^{1}**H NMR** (CD_3OD, 400 MHz): δ (ppm) 8.13 – 8.12 (m, 1H), 7.92 (td, *J* = 8.4, 2.6 Hz, 1H), 7.02 (dd, *J* = 8.4, 2.6 Hz, 1H), 3.93 (dd, *J* = 5.6, 2.9 Hz, 1H), 3.86 (dd, *J* = 9.4, 5.6 Hz, 1H), 3.63 (d, *J* = 5.6 Hz, 2H), 3.21 – 3.14 (m, 1H), 2.25 – 2.13 (m, 3H), 1.48 – 1.42 (m, 1H). ^{13}**C NMR** (101 MHz, CD_3OD): δ (ppm) 163.8 (d, *J* = 237.3 Hz), 147.4 (d, *J* = 13.4 Hz), 142.2 (d, *J* = 7.9 Hz), 138.1 (d, *J* = 4.3 Hz), 110.3 (d, *J* = 36.6 Hz), 79.8, 75.2, 64.7, 47.2, 32.3, 25.0. ^{19}**F NMR** (376 MHz, CD_3OD) δ –75.1 (d, *J* = 7.5 Hz). **$[\alpha]^{25}_{D}$** = –40.7 (c = 1.0, CH_3OH).

HRMS (ESI): m/z calcd for $C_{11}H_{15}O_3NF^+$ $[M + H]^+$ 228.1030 found 228.1030.

IR (ν_{max}/cm^{-1}) 3349, 2929, 2890, 2361, 2341, 1601, 1488, 1407, 1347, 1254, 1111, 1070, 1029, 968, 902, 834, 808, 749.

(1R,2S,3S,5R)-3-(2-fluoropyridin-4-yl)-5-(hydroxymethyl) cyclopentane-1,2-diol (7e):

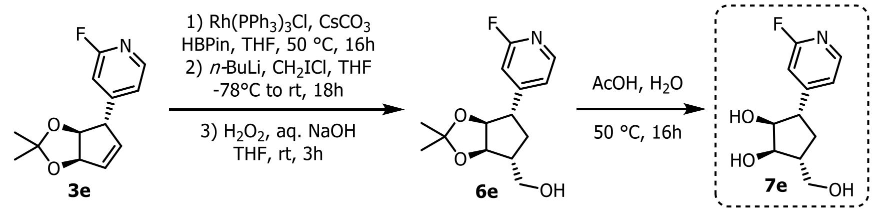

According to modified procedure by Srebnik et. al,[3] [Rh(PPh$_3$)$_3$Cl] (23.1 mg, 0.025 mmol, 2.5 mol%) and Cs_2CO_3 (325.8 mg, 1.0 mmol, 1 equiv) were added to a flame dried 25 mL round bottom flask, sealed with a rubber septum under an argon atmosphere, dissolved in THF (0.5 mL) and stirred at room temperature. After 5 min, a solution (or suspension) of **3e** (1 mmol, 1.0 equiv) in THF (3 mL) was added via syringe and the flask was rinsed with THF (0.5 mL). Pinacolborane (0.29 mL, 2 mmol, 2.0 equiv) was added dropwise to the reaction mixture at 50 °C and stirred for 16 hours at 50 °C. The mixture was then cooled to room temperature and diluted with Et_2O (5 mL) before passing through a plug of silica. The plug was washed with an additional 10 mL of Et_2O and the solvents were removed in vacuo. The crude product **4e** was charged to the next step without further purification.

In a flamed dried flask under nitrogen, *n*-butyllithium (2.5 M in hexanes, 0.75 mL, 1.88 mmol, 2.5 equiv) was added dropwise to a solution of pinacol boronic ester **4e** (0.75 mmol, 1.0 equiv) and iodochloromethane (165 µL, 2.25 mmol, 3 equiv) in THF (3.5 mL) at -78 °C. The reaction mixture was slowly allowed to warm to ambient temperature over 12 h. The mixture was diluted with Et_2O (5 mL) and the organic layer was washed with an aq. sat. solution of NH_4Cl (2 x 3 mL) and dried over Na_2SO_4, filtered, and the solvent was removed in vacuo. The crude product **5e** was charged to the next step without further purification.

30%wt. H_2O_2 (1 mL) was added to a solution of pinacol boronic ester **5e** (0.75 mmol, 1.0 equiv) and 2N aq. NaOH (1.5 ml) in THF (4 mL) at 0 °C. The reaction mixture was slowly allowed to warm to ambient temperature. After 3h, the mixture was diluted with Et_2O (7 mL). The organic layer was separated and the aqueous layer was extracted with Et_2O (2 × 5 mL). The combined organic layers were dried over anhydrous Na_2SO_4, filtered, and the solvent was removed in vacuo. Purification by flash chromatography (15% Et_2O/petrol to 30% Et_2O/petrol) afforded compound **6e** as a colourless oil (49% yield over 3 steps) as a single diastereomer (dr >20:1).

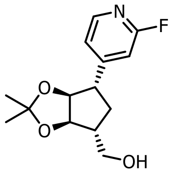

((3aR,4R,6S,6aS)-6-(2-fluoropyridin-4-yl)-2,2-dimethyltetrahydro-4H-cyclopenta [d] [1,3] dioxol-4-yl) methanol (6e): **^{1}H NMR** ($CDCl_3$, 400 MHz): δ (ppm) 8.13 (d, *J* = 5.2 Hz, 1H), 7.11 (dddd, *J* = 5.2, 2.1, 1.5, 0.7 Hz, 1H), 6.85 (tt, *J* = 1.5, 0.7 Hz, 1H), 4.54 (dd, *J* = 7.2, 4.8 Hz, 1H), 4.48 (t, *J* = 7.2 Hz, 1H), 3.82 (dd, *J* = 10.5, 5.4 Hz, 1H), 3.73 (dd, *J* = 10.5, 6.3 Hz, 1H), 3.23 (dt, *J* = 12.8, 6.6 Hz, 1H), 2.41 (tdd, *J* = 11.5, 6.5, 5.0 Hz, 1H), 2.31 (dt, *J* = 12.8, 6.5 Hz, 1H), 1.73 (td, *J* = 12.8, 11.5 Hz, 1H), 1.57 (s, 3H), 1.33 (s, 3H). **^{13}C NMR** (101 MHz, $CDCl_3$): δ (ppm) 164.2 (d, *J* = 238.9 Hz), 157.4 (d, *J* = 7.7 Hz), 147.6 (d, *J* = 15.0 Hz), 120.4 (d, *J* = 3.8 Hz), 113.8, 108.1 (d, *J* = 37.1 Hz), 86.2, 82.9, 63.9, 50.2 (d, *J* = 2.8 Hz), 47.1, 27.7, 25.2. **^{19}F NMR** (376 MHz, $CDCl_3$) δ –75.1 (d, *J* = 7.5 Hz). **$[\alpha]^{25}_D$** = –53.9 (c = 1.0, $CHCl_3$).

HRMS (ESI): m/z calcd for $C_{14}H_{19}O_3NF^+$ $[M + H]^+$ 268.1343 found 268.1343.

IR (ν_{max}/cm^{-1}) 3372, 2988, 2935, 2360, 2341, 1614, 1558, 1507, 1484, 1456, 1416, 1381, 1276, 1256, 1217, 1161, 1068, 1011, 981, 865, 772.

A solution of alcohol **6e** (0.2 mmol) in AcOH (0.5 mL) and H_2O (0.5 mL) was stirred at 50 °C for 16 h. The reaction mixture was concentrated under reduced pressure and purification by flash chromatography (1% MeOH/EtOAc to 10% MeOH/EtOAc) afforded compound **7e** as a colourless oil (76% yield) as a single diastereomer (dr >20:1).

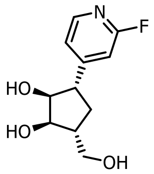

(1R,2S,3S,5R)-3-(2-fluoropyridin-4-yl)-5-(hydroxymethyl)cyclopentane-1,2-diol (7e): **^{1}H NMR** (CD_3OD, 400 MHz): δ (ppm) 8.11 (d, *J* = 5.3 Hz, 1H), 7.31 (dt, *J* = 5.4, 1.8 Hz, 1H), 7.07 (s, 1H), 3.98 – 3.89 (m, 2H), 3.64 (d, *J* = 5.6 Hz, 2H), 3.23 (qd, *J* = 8.1, 5.4 Hz, 1H), 2.32 – 2.12 (m, 2H), 1.57 – 1.43 (m, 1H). **^{13}C NMR** (101 MHz, CD_3OD): δ (ppm) 172.9, 148.1, 122.3, 109.7, 109.3, 79.1, 75.2, 67.0, 45.3, 31.8, 20.8. **^{19}F NMR** (376 MHz, CD_3OD): δ –72.5 (d, *J* = 2.0 Hz). **$[\alpha]^{25}_D$** = –46.6 (c = 1.0, CH_3OH).

HRMS (ESI): m/z calcd for $C_{11}H_{15}O_3NF^+$ $[M + H]^+$ 228.1030 found 228.1030.

IR (ν_{max}/cm^{-1}) 3359, 2937, 2361, 2341, 1734, 1615, 1559, 1540, 1481, 1456, 1417, 1367, 1336, 1320, 1281, 1247, 1209, 1160, 1137, 1097, 1027, 982, 838, 769.

(1R,2S,3R,5R)-3-(furan-2-yl)-5-(hydroxymethyl) cyclopentane-1,2-diol (7h):

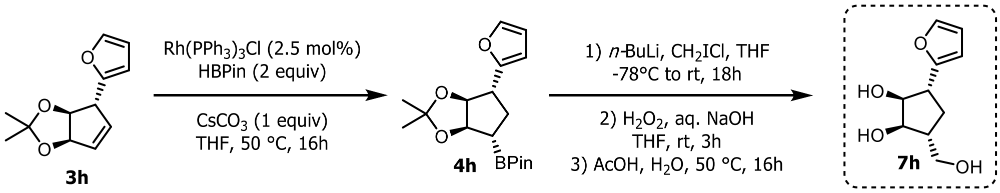

According to modified procedure by Srebnik et. al,[3] [Rh(PPh_3)$_3$Cl] (46.3 mg, 0.05 mmol, 2.5 mol%) and Cs_2CO_3 (651.6 mg, 2.0 mmol, 1 equiv) were added to a flame dried 25 mL round bottom flask, sealed with a rubber septum under an argon atmosphere, dissolved in THF (1 mL) and stirred at room temperature. After 5 min, a solution (or suspension) of **3h** (2 mmol, 1.0 equiv) in THF (5 mL) was added via syringe and the flask was rinsed with THF (1 mL). Pinacolborane (0.58 mL, 4 mmol, 2.0 equiv) was added dropwise to the reaction mixture at 50 °C and stirred for 16 hours at 50 °C. The mixture was then cooled to room temperature and diluted with Et_2O (10 mL) before passing through a plug of celite. The plug was washed with an additional 15 mL of Et_2O and the solvents were removed in vacuo. Purification by flash chromatography (10% Et_2O/petrol to 25% Et_2O/petrol) afforded compound **4h** as a colourless oil (74% yield) as a single diastereomer (dr >20:1).

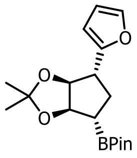

((3aR,4S,6R,6aS)-6-(furan-2-yl)-2,2-dimethyltetrahydro-4H-cyclopenta[d][1,3]dioxol-4-yl) boronic acid pinacol ester (4h): **^{1}H NMR** (400 MHz, $CDCl_3$): δ (ppm) 7.31 (dd, *J* = 1.9, 0.9 Hz, 1H), 6.25 (dd, *J* = 3.2, 1.9 Hz, 1H), 6.06 (dt, *J* = 3.2, 0.9 Hz, 1H), 4.82 (dd, *J* = 6.3, 3.8 Hz, 1H), 4.71 (dd, *J* = 6.3, 3.8 Hz, 1H), 3.29 – 3.21 (m, 1H), 2.37 (ddd, *J* = 12.8, 8.2, 7.0 Hz, 1H), 1.95 (dt, *J* = 12.8, 7.5 Hz, 1H), 1.63 (td, *J* = 8.2, 3.8 Hz, 1H), 1.51 (s, 3H), 1.33 (s, 3H), 1.17 (s, 12H). **^{13}C NMR** (101 MHz, $CDCl_3$) δ 155.9, 141.6, 111.7, 110.1, 105.1, 85.3, 83.6, 83.1, 45.9, 31.6, 27.4, 25.00, 24.97. **$[\alpha]^{25}_D$** = –26.1 (c = 1.0, $CHCl_3$).

HRMS (ESI): m/z calcd for $C_{18}H_{27}O_5BNa^+$ [M + Na]$^+$ 357.1844 found 357.1844.

IR (ν_{max}/cm^{-1}) 2980, 2936, 2361, 1507, 1457, 1379, 1323, 1269, 1210, 1144, 1050, 1030, 973, 904, 855, 733.

In a flamed dried flask under nitrogen, *n*-butyllithium (2.5 M in hexanes, 1.5 mL, 1.88 mmol, 2.5 equiv) was added dropwise to a solution of pinacol boronic ester **4h** (1.5 mmol, 1.0 equiv) and iodochloromethane (330 µL, 4.5 mmol, 3 equiv) in THF (7 mL) at -78 °C. The reaction mixture was slowly allowed to warm to ambient temperature over 12 h. The mixture was diluted with Et_2O (10 mL) and the organic layer was washed with an aq. sat. solution of NH_4Cl (2 x 5 mL) and dried over Na_2SO_4, filtered, and the solvent was removed in vacuo. The crude product **5h** was charged to the next step without further purification.

30%wt. H_2O_2 (2 mL) was added to a solution of pinacol boronic ester **5h** (1.5 mmol, 1.0 equiv) and 2N aq. NaOH (3 ml) in THF (7 mL) at 0 °C. The reaction mixture was slowly allowed to warm to ambient temperature. After 3h, the mixture was diluted with Et_2O (10 mL). The organic layer was separated, and the aqueous layer was extracted with Et_2O (2 × 7 mL). The combined organic layers were dried over anhydrous Na_2SO_4, filtered, and the solvent was removed in vacuo. The crude product **6h** was charged to the next step without further purification.

A solution of alcohol **6h** (0.2 mmol) in AcOH (0.5 mL) and H_2O (0.5 mL) was stirred at 50 °C for 16 h. The reaction mixture was concentrated under reduced pressure and purification by flash chromatography (1% MeOH/EtOAc to 10% MeOH/EtOAc) afforded compound **7h** as a colourless oil (50% yield over 3 steps) as a single diastereomer (dr >20:1).

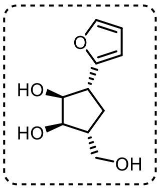

(1R,2S,3R,5R)-3-(furan-2-yl)-5-(hydroxymethyl)cyclopentane-1,2-diol (7h): ^{1}H NMR (CD_3OD, 400 MHz): δ (ppm) 7.38 (dd, *J* = 1.9, 0.8 Hz, 1H), 6.31 (dd, *J* = 3.2, 1.9 Hz, 1H), 6.13 (dd, *J* = 3.2, 0.9 Hz, 1H), 3.97 (dd, *J* = 7.5, 5.1 Hz, 1H), 3.85 (t, *J* = 5.1 Hz, 1H), 3.64 (dd, *J* = 10.8, 5.9 Hz, 1H), 3.56 (dd, *J* = 10.8, 5.9 Hz, 1H), 3.19 (dt, *J* = 10.1, 7.5 Hz, 1H), 2.32 – 2.08 (m, 2H), 1.58 – 1.41 (m, 1H).**^{13}C NMR** (101 MHz, CD_3OD): δ (ppm) 158.1, 142.3, 111.1, 105.8, 78.0, 75.4, 64.9, 47.5, 44.3, 30.4. **$[\alpha]^{25}_{D}$** = –43.1 (c = 1.0, CH_3OH).

HRMS (ESI): m/z calcd for $C_{10}H_{14}O_4Na^+$ $[M + Na]^+$ 221.0784 found 221.0784.

IR (ν_{max}/cm^{-1}) 3366, 2928, 2361, 2341, 1642, 1508, 1412, 1343, 1228, 1147, 1105, 1072, 1031, 1013, 968, 927, 884.

2-((3aS,4S,6R,6aR)-6-(hydroxymethyl)-2,2-dimethyltetrahydro-4H-cyclopenta [d] [1,3] dioxol-4-yl)-(tert-butoxycarbonyl)-1H-pyrrole (6j):

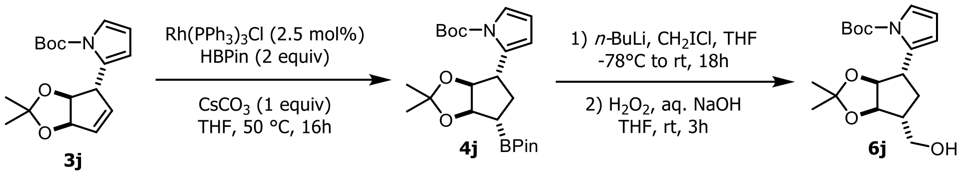

According to modified procedure by Srebnik et. al,[3] [Rh(PPh_3)$_3$Cl] (23.1 mg, 0.025 mmol, 2.5 mol%) and Cs_2CO_3 (325.8 mg, 1.0 mmol, 1 equiv) were added to a flame dried 25 mL round bottom flask, sealed with a rubber septum under an argon atmosphere, dissolved in THF (0.5 mL) and stirred at room temperature. After 5 min, a solution (or suspension) of **3j** (1 mmol, 1.0 equiv) in THF (3 mL) was added via syringe and the flask was rinsed with THF (0.5 mL). Pinacolborane (0.29 mL, 2 mmol, 2.0 equiv) was added dropwise to the reaction mixture at 50 °C and stirred for 16 hours at 50 °C. The mixture was then cooled to room temperature and diluted with Et_2O (5 mL) before passing through a plug of celite. The plug was washed with an additional 10 mL of Et_2O and the solvents were removed in vacuo. Purification by flash chromatography (10% Et_2O/petrol to 25% Et_2O/petrol) afforded compound **4j** as a colourless oil (66% yield) as a single diastereomer (dr >20:1).

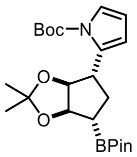

((3aR,4S,6S,6aS)-6-(1-(tert-butoxycarbonyl)-1H-pyrrol-2-yl)-2,2-dimethyl tetrahydro-4H-cyclopenta[d][1,3]dioxol-4-yl)boronic acid pinacol ester (4j): ^{1}H NMR ($CDCl_3$, 400 MHz): δ (ppm) 7.20 (dd, J = 3.4, 1.7 Hz, 1H), 6.03 (t, J = 3.4 Hz, 1H), 5.97 (ddd, J = 3.4, 1.7, 1.0 Hz, 1H), 4.90 (dd, J = 6.1, 3.1 Hz, 1H), 4.74 (dd, J = 6.2, 3.5 Hz, 1H), 3.82 (td, J = 6.2, 3.5 Hz, 1H), 2.52 (ddd, J = 12.4, 8.8, 6.9 Hz, 1H), 1.71 (dt, J = 12.4, 6.1 Hz, 1H), 1.59 (s, 9H), 1.49 (s, 3H), 1.33 (s, 3H), 1.17 (s, 6H), 1.10 (s, 6H). **^{13}C NMR** (101 MHz, $CDCl_3$): δ (ppm) 149.5, 136.2, 121.9, 110.9, 110.3, 109.9, 85.8, 83.7, 83.5, 83.1, 45.4, 33.9, 28.2, 27.4, 25.2, 25.0, 24.8. **$[\alpha]^{25}_D$** = –33.9 (c = 1.0, $CHCl_3$).

HRMS (ESI): m/z calcd for $C_{23}H_{36}O_6NBNa^+$ $[M + Na]^+$ 456.2530 found 456.2524.

IR (ν_{max}/cm^{-1}) 2935, 2360, 2341, 1742, 1474, 1459, 1255, 1212, 1145, 1120, 1065, 1030, 992, 971, 891, 852, 773, 726.

In a flamed dried flask under nitrogen, *n*-butyllithium (2.5 M in hexanes, 0.75 mL, 1.88 mmol, 2.5 equiv) was added dropwise to a solution of pinacol boronic ester **4j** (0.75 mmol, 1.0 equiv) and iodochloromethane (165 µL, 2.25 mmol, 3 equiv) in THF (3.5 mL) at -78 °C. The reaction mixture was slowly allowed to warm to ambient temperature over 12 h. The mixture was diluted with Et_2O (5 mL) and the organic layer was washed with an aq. sat. solution of NH_4Cl (2 x 3 mL) and dried over Na_2SO_4, filtered, and the solvent was removed in vacuo. The crude product **5j** was charged to the next step without further purification.

30%wt. H_2O_2 (1 mL) was added to a solution of pinacol boronic ester **5j** (0.75 mmol, 1.0 equiv) and 2N aq. NaOH (1.5 ml) in THF (4 mL) at 0 °C. The reaction mixture was slowly allowed to warm to ambient temperature. After 3h, the mixture was diluted with Et_2O (7 mL). The organic layer was separated and the aqueous layer was extracted with Et_2O (2 × 5 mL). The combined organic layers were dried over anhydrous Na_2SO_4, filtered, and the solvent was removed in vacuo. Purification by flash chromatography (15% Et_2O/petrol to 30% Et_2O/petrol) afforded compound **6j** as a colourless oil (87% yield over 2 steps) as a single diastereomer (dr >20:1).

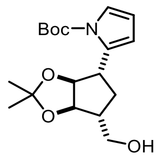

2-((3aS,4S,6R,6aR)-6-(hydroxymethyl)-2,2-dimethyltetrahydro-4H-cyclopenta[d][1,3] dioxol-4-yl)-(tert-butoxycarbonyl)-1H-pyrrole (6j): **^{1}H NMR** ($CDCl_3$, 400 MHz): δ (ppm) 7.21 (dd, *J* = 3.4, 1.8 Hz, 1H), 6.14 (ddd, *J* = 3.4, 1.8, 0.9 Hz, 1H), 6.10 (t, *J* = 3.4 Hz, 1H), 4.78 (t, *J* = 6.5 Hz, 1H), 4.56 (dd, *J* = 6.9, 4.6 Hz, 1H), 3.92 – 3.82 (m, 1H), 3.71 (dd, *J* = 10.6, 5.7 Hz, 1H), 3.64 (dd, *J* = 10.6, 7.2 Hz, 1H), 2.46 (dt, *J* = 12.4, 6.9 Hz, 1H), 2.42 – 2.32 (m, 1H), 1.61 (s, 9H), 1.55 (s, 3H), 1.46 (dt, *J* = 12.1, 10.4 Hz, 1H), 1.36 (s, 3H). **^{13}C NMR** (101 MHz, $CDCl_3$): δ (ppm) 149.3, 136.2, 121.8, 112.7, 110.4, 110.0, 85.0, 83.6, 83.0, 64.7, 47.8, 44.3, 35.6, 28.0, 27.6, 25.3, 24.9.

*Attempts towards acetal deprotection of **6j** led to decomposition.

3-((1S,2S,3R,4R)-2,3-dihydroxy-4-(hydroxymethyl)cyclopentyl)-1H-pyrrole-2,5-dione (6k):

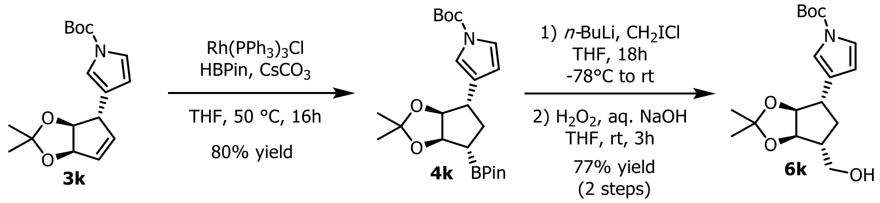

According to modified procedure by Srebnik et. al,[3] [Rh(PPh_3)$_3$Cl] (46.3 mg, 0.05 mmol, 2.5 mol%) and Cs_2CO_3 (651.6 mg, 2.0 mmol, 1 equiv) were added to a flame dried 25 mL round bottom flask, sealed with a rubber septum under an argon atmosphere, dissolved in THF (1 mL) and stirred at room temperature. After 5 min, a solution (or suspension) of **3k** (2 mmol, 1.0 equiv) in THF (5 mL) was added via syringe and the flask was rinsed with THF (1 mL). Pinacolborane (0.58 mL, 4 mmol, 2.0 equiv) was added dropwise to the reaction mixture at 50 °C and stirred for 16 hours at 50 °C. The mixture was then cooled to room temperature and diluted with Et_2O (10 mL) before passing through a plug of celite. The plug was washed with an additional 15 mL of Et_2O and the solvents were removed in vacuo. Purification by flash chromatography (10% Et_2O/petrol to 25% Et_2O/petrol) afforded compound **4k** as a colourless oil (80% yield) as a single diastereomer (dr >20:1).

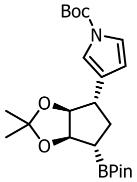

((3aR,4S,6S,6aS)-6-(1-(tert-butoxycarbonyl)-1H-pyrrol-3-yl)-2,2-dimethyl tetrahydro-4H-cyclopenta[d][1,3]dioxol-4-yl)boronic acid pinacol ester (4k): ^{1}H NMR ($CDCl_3$, 400 MHz): δ (ppm) 7.15 (t, J = 2.5 Hz, 1H), 7.01 (d, J = 2.5 Hz, 1H), 6.14 (dd, J = 3.3, 1.7 Hz, 1H), 4.81 (dd, J = 6.4, 3.9 Hz, 1H), 4.57 (dd, J = 6.4, 4.2 Hz, 1H), 3.06 (td, J = 7.0, 4.2 Hz, 1H), 2.44 – 2.25 (m, 1H), 1.77 (dt, J = 12.5, 7.7 Hz, 1H), 1.63 – 1.58 (m, 1H), 1.56 (s, 9H), 1.51 (s, 3H), 1.33 (s, 3H), 1.14 (s, 12H).**^{13}C NMR** (101 MHz, $CDCl_3$): δ (ppm) 149.0, 128.4, 120.5, 116.3, 112.3, 111.5, 86.8, 83.5, 83.4, 83.1, 44.7, 33.4, 28.1, 27.5, 25.1, 24.80, 24.79. **$[\alpha]^{25}_D$** = –13.1 (c = 1.0, $CHCl_3$).

HRMS (ESI): m/z calcd for $C_{23}H_{36}O_6NBNa^+$ $[M + Na]^+$ 456.2530 found 456.2525.

IR (ν_{max}/cm^{-1}) 2981, 2935, 2361, 2341, 1742, 1482, 1458, 1395, 1371, 1347, 1323, 1251, 1211, 1161, 1146, 1068, 1030, 973, 854, 771.

In a flamed dried flask under nitrogen, *n*-butyllithium (2.5 M in hexanes, 1.5 mL, 3.75 mmol, 2.5 equiv) was added dropwise to a solution of pinacol boronic ester **4k** (1.5 mmol, 1.0 equiv) and iodochloromethane (330 µL, 4.5 mmol, 3 equiv) in THF (7 mL) at -78 °C. The reaction mixture was slowly allowed to warm to ambient temperature over 12 h. The mixture was diluted with Et_2O (10 mL) and the organic layer was washed with an aq. sat. solution of NH_4Cl (2 x 5 mL) and dried over Na_2SO_4, filtered, and the solvent was removed in vacuo. The crude product **5k** was charged to the next step without further purification.

30%wt. H_2O_2 (2 mL) was added to a solution of pinacol boronic ester **5k** (1.5 mmol, 1.0 equiv) and 2N aq. NaOH (3 ml) in THF (7 mL) at 0 °C. The reaction mixture was slowly allowed to warm to ambient temperature. After 3h, the mixture was diluted with Et_2O (10 mL). The organic layer was separated and the aqueous layer was extracted with Et_2O (2 × 7 mL). The combined organic layers were dried over anhydrous Na_2SO_4, filtered, and the solvent was removed in vacuo. Purification by flash chromatography (15% Et_2O/petrol to 30% Et_2O/petrol) afforded compound **7o** as a colourless oil (77% yield over 2 steps) as a single diastereomer (dr >20:1).

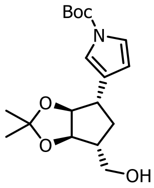

3-((3aS,4S,6R,6aR)-6-(hydroxymethyl)-2,2-dimethyltetrahydro-4H-cyclopenta[d][1,3] dioxol-4-yl)-1-(tert-butoxycarbonyl)-1H-pyrrole (6k): ^{1}H NMR ($CDCl_3$, 400 MHz): δ (ppm) 7.18 (dt, *J* = 3.4, 1.7 Hz, 1H), 7.08 – 7.03 (m, 1H), 6.15 (dd, *J* = 3.4, 1.7 Hz, 1H), 4.50 – 4.42 (m, 2H), 3.72 (dd, *J* = 10.6, 5.8 Hz, 1H), 3.65 (dd, *J* = 10.6, 6.9 Hz, 1H), 3.15 – 3.02 (m, 1H), 2.33 (dddd, *J* = 10.6, 9.6, 8.4, 6.2 Hz, 1H), 2.24 (dt, *J* = 13.2, 6.7 Hz, 1H), 1.61 – 1.58 (m, 1H), 1.57 (s, 9H), 1.54 (s, 3H), 1.32 (s, 3H). **^{13}C NMR** (101 MHz, $CDCl_3$): δ (ppm) 149.0, 128.5, 120.7, 116.1, 113.1, 111.5, 86.9, 83.6, 83.3, 64.7, 47.9, 43.7, 28.1, 27.7, 25.3, 25.0.

*Attempts towards acetal deprotection of **6k** led to decomposition.

(1R,2S,3S,5R)-3-(2,6-difluoropyridin-4-yl)-5-(hydroxymethyl)cyclopentane-1,2-diol (7l):

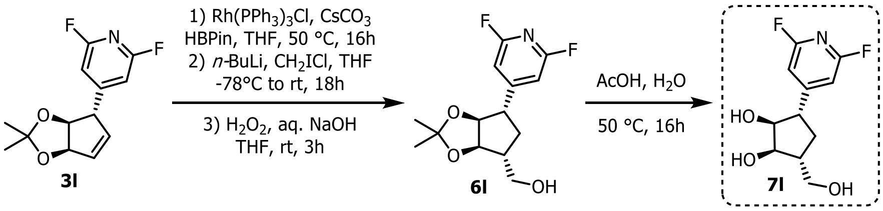

According to modified procedure by Srebnik et. al,[3] [Rh(PPh_3)$_3$Cl] (23.1 mg, 0.025 mmol, 2.5 mol%) and Cs_2CO_3 (325.8 mg, 1.0 mmol, 1 equiv) were added to a flame dried 25 mL round bottom flask, sealed with a rubber septum under an argon atmosphere, dissolved in THF (0.5 mL) and stirred at room temperature. After 5 min, a solution (or suspension) of **3l** (1 mmol, 1.0 equiv) in THF (3 mL) was added via syringe and the flask was rinsed with THF (0.5 mL). Pinacolborane (0.29 mL, 2 mmol, 2.0 equiv) was added dropwise to the reaction mixture at 50 °C and stirred for 16 hours at 50 °C. The mixture was then cooled to room temperature and diluted with Et_2O (5 mL) before passing through a plug of silica. The plug was washed with an additional 10 mL of Et_2O and the solvents were removed in vacuo. The crude product **4l** was charged to the next step without further purification.

In a flamed dried flask under nitrogen, *n*-butyllithium (2.5 M in hexanes, 0.75 mL, 1.88 mmol, 2.5 equiv) was added dropwise to a solution of pinacol boronic ester **4l** (0.75 mmol, 1.0 equiv) and iodochloromethane (165 µL, 2.25 mmol, 3 equiv) in THF (3.5 mL) at -78 °C. The reaction mixture was slowly allowed to warm to ambient temperature over 12 h. The mixture was diluted with Et_2O (5 mL) and the organic layer was washed with an aq. sat. solution of NH_4Cl (2 x 3 mL) and dried over Na_2SO_4, filtered, and the solvent was removed in vacuo. The crude product **5l** was charged to the next step without further purification.

30%wt. H_2O_2 (1 mL) was added to a solution of pinacol boronic ester **5l** (0.75 mmol, 1.0 equiv) and 2N aq. NaOH (1.5 ml) in THF (4 mL) at 0 °C. The reaction mixture was slowly allowed to warm to ambient temperature. After 3h, the mixture was diluted with Et_2O (7 mL). The organic layer was separated and the aqueous layer was extracted with Et_2O (2 × 5 mL). The combined organic layers were dried over anhydrous Na_2SO_4, filtered, and the solvent was removed in vacuo. Purification by flash chromatography (15% Et_2O/petrol to 30% Et_2O/petrol) afforded compound **6l** as a colourless oil (45% yield over 3 steps) as a single diastereomer (dr >20:1).

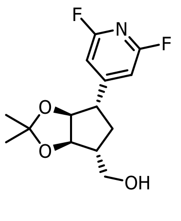

((3aR,4R,6S,6aS)-6-(2,6-difluoropyridin-4-yl)-2,2-dimethyltetrahydro-4H-cyclopenta [d] [1,3] dioxol-4-yl) methanol (6l): ^{1}**H NMR** ($CDCl_3$, 400 MHz): δ (ppm) 6.78 (s, 2H), 4.57 (dd, *J* = 7.2, 4.7 Hz, 1H), 4.48 (t, *J* = 7.2 Hz, 1H), 3.84 (dd, *J* = 10.5, 5.3 Hz, 1H), 3.75 (dd, *J* = 10.5, 6.1 Hz, 1H), 3.27 (dt, *J* = 13.0, 6.7 Hz, 1H), 2.48 – 2.38 (m, 1H), 2.34 (dt, *J* = 13.0, 6.7 Hz, 1H), 1.75 (td, *J* = 12.7, 11.6 Hz, 1H), 1.59 (s, 3H), 1.33 (s, 3H). ^{13}**C NMR** (101 MHz, $CDCl_3$):

δ (ppm) 162.1, 162.0 (d, J = 262.4 Hz), 162.0 (d, J = 230.2 Hz), 114.0, 104.7 (d, J = 39.9 Hz), 104.7 (d, J = 13.2 Hz), 86.0, 82.8, 63.9, 50.4, 46.9, 33.5, 27.7, 25.2. ^{19}**F NMR** (376 MHz, $CDCl_3$) δ –68.9. **$[\alpha]^{25}_D$** = –46.5 (c = 1.0, $CHCl_3$).

A solution of alcohol **6l** (0.2 mmol) in AcOH (0.5 mL) and H_2O (0.5 mL) was stirred at 50 °C for 16 h. The reaction mixture was concentrated under reduced pressure and purification by flash chromatography (1% MeOH/EtOAc to 10% MeOH/EtOAc) afforded compound **7l** as a colourless oil (62% yield) as a single diastereomer (dr >20:1).

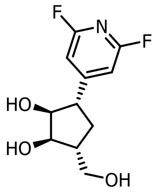

(1R,2S,3S,5R)-3-(2,6-difluoropyridin-4-yl)-5-(hydroxymethyl)cyclopentane-1,2-diol (7l): ^{1}**H NMR** (CD_3OD, 400 MHz): δ (ppm) 6.94 (s, 2H), 3.94 – 3.83 (m, 2H), 3.60 (d, J = 5.6 Hz, 2H), 3.28 – 3.16 (m, 1H), 2.28 – 2.10 (m, 2H), 1.51 – 1.36 (m, 1H). ^{13}**C NMR** (101 MHz, CD_3OD): δ (ppm) 165.9 (t, J = 7.7 Hz), 163.3 (d, J = 244.0 Hz), 163.1 (d, J = 244.2 Hz), 106.0 (d, J = 39.9 Hz), 106.0 (d, J = 13.1 Hz), 79.4, 75.3, 64.6, 50.2 (t, J = 2.6 Hz), 48.2, 31.6. ^{19}**F NMR** (376 MHz, CD_3OD) δ –72.7. **$[\alpha]^{25}_D$** = –30.0 (c = 1.0, CH_3OH).

HRMS (ESI): m/z calcd for $C_{11}H_{14}O_3NF_2^+$ $[M + H]^+$ 246.0936 found 246.0936.

IR (ν_{max}/cm^{-1}) 3375, 2923, 2854, 1628, 1568, 1425, 1376, 1345, 1199, 1110, 1071, 1024, 852, 694.

4. Suzuki-Miyaura Coupling Attempts with Complex Boronic Acids

Synthesis of Boronic Acid/Ester (Failed Substrates)

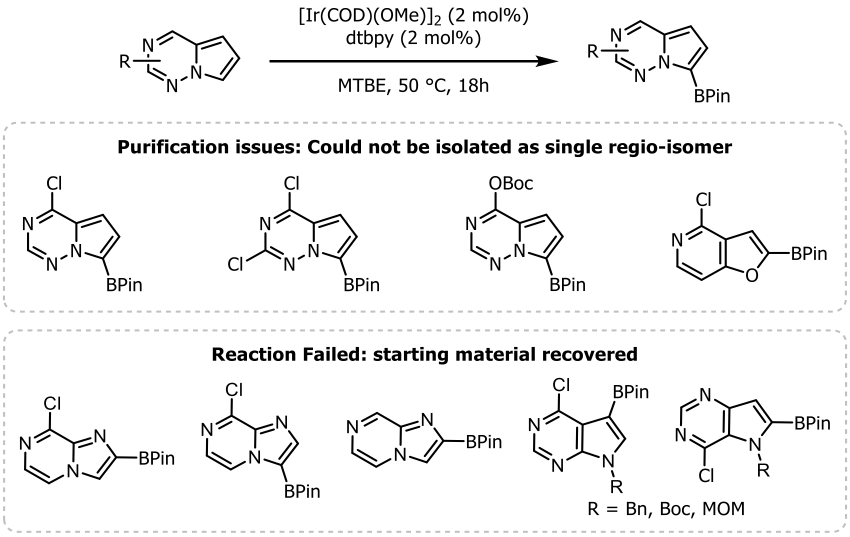

Rhodium-Catalysed SMC Reaction (Failed Substrates)

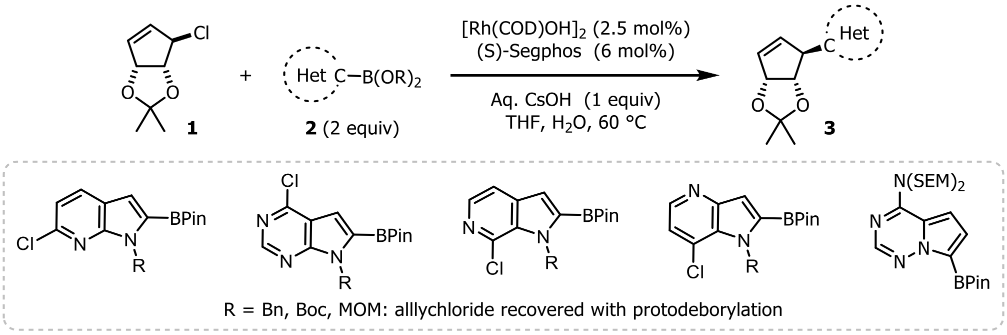

5. Synthesis of Amino Pyrrolo Triazine Derived CC-Ns

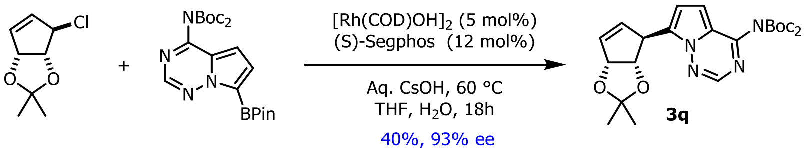

(7-((3aS,4S,6aR)-2,2-dimethyl-3a,6a-dihydro-4H-cyclopenta [d] [1,3] dioxol-4-yl) pyrrolo [2,1-f] [1,2,4] triazin-4-yl)di-tert-butyliminodicarbonate (3q). The corresponding compound was prepared following modified general procedure **A** using (4-(bis(tert-butoxycarbonyl)amino)pyrrolo [2,1-f] [1,2,4] triazin-7-yl) boronic acid pinacol ester with 5 mol% of Rh dimer and 12 mol% of (*S*)-Segphos. The mixture was stirred at 60 °C for 18 hours. Purification by flash chromatography (10% Et_2O/petrol to 25% Et_2O/petrol) afforded compound **3q** as a colourless oil (90% yield, 93% ee) as a single diastereomer (dr >20:1).

^{1}H NMR ($CDCl_3$, 400 MHz): δ (ppm) 8.43 (s, 1H), 6.71 (d, *J* = 4.6 Hz, 1H), 6.61 (d, *J* = 4.6 Hz, 1H), 6.08 (dt, *J* = 5.8, 1.9 Hz, 1H), 5.96 (ddt, *J* = 5.8, 2.4, 0.9 Hz, 1H), 5.31 (dq, *J* = 5.7, 1.4 Hz, 1H), 4.67 – 4.65 (m, 2H), 1.50 (s, 3H), 1.43 (s, 18H), 1.36 (s, 3H). **^{13}C NMR** (101 MHz, $CDCl_3$): δ (ppm) 152.7, 150.1, 146.6, 133.7, 132.1, 131.9, 120.8, 113.2, 111.0, 102.8, 85.5, 84.3, 82.8, 48.9, 27.9, 27.7, 26.1.

HRMS (ESI): m/z calcd for $C_{24}H_{33}O_6N_4^+$ [M + H]$^+$ 473.2395 found 473.2397.

IR (ν_{max}/cm^{-1}) 2984, 2936, 2361, 2341, 1798, 1761, 1600, 1506, 1450, 1418, 1370, 1313, 1272, 1254, 1211, 1159, 1077, 1050, 943, 873, 852, 772, 731.

SFC Conditions: Chiralpak IC; 1500 PSI, 30 °C; flow: 1.5 mL/min; from 1% to 30% MeOH in 5 min; 96.5:3.5 er (major enantiomer t_R = 2.76 min; minor enantiomer t_R = 2.63 min), **93% ee**. **$[\alpha]^{25}_D$** = –38.9 (c = 1.0, $CHCl_3$).

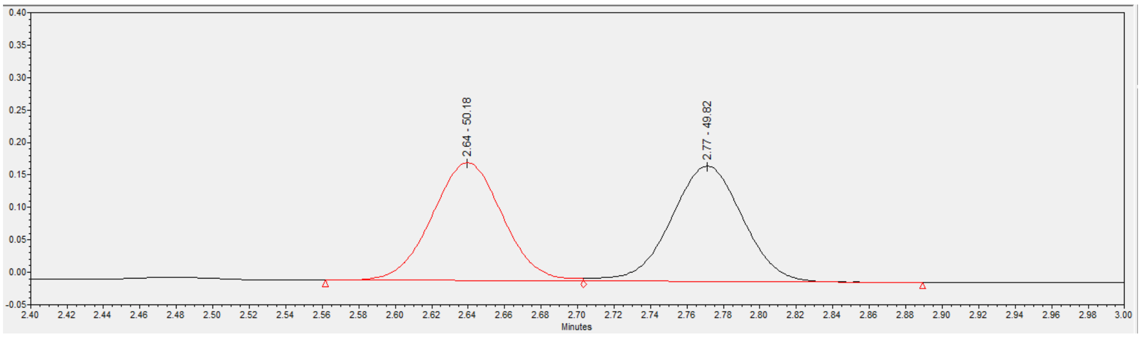

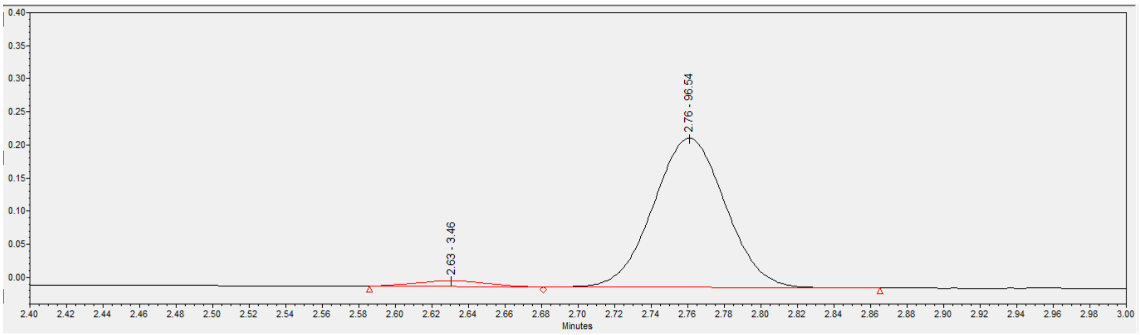

(1S,2R,3S,4S)-4-(4-amino pyrrolo [2,1-f] [1,2,4] triazin-7-yl) cyclo pentane-1,2,3-triol trifluoroacetic acid (9q):

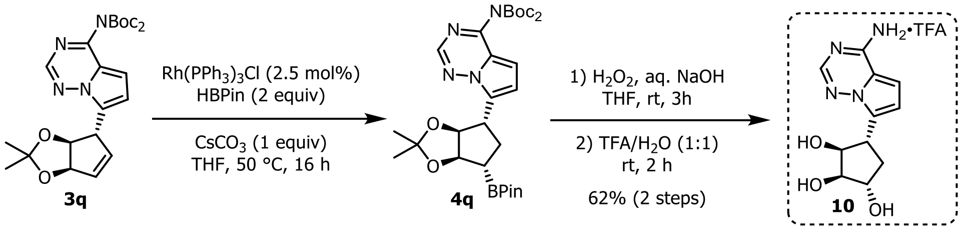

According to modified procedure by Srebnik et. al,[3] [Rh(PPh_3)$_3$Cl] (46.3 mg, 0.05 mmol, 5 mol%) and Cs_2CO_3 (325.8 mg, 1.0 mmol, 1 equiv) were added to a flame dried 25 mL round bottom flask, sealed with a rubber septum under an argon atmosphere, dissolved in THF (1 mL) and stirred at room temperature. After 5 min, a solution (or suspension) of **3q** (1 mmol, 1.0 equiv) in THF (2 mL) was added via syringe and the flask was rinsed with THF (1 mL). Pinacolborane (0.29 mL, 2 mmol, 2.0 equiv) was added dropwise to the reaction mixture at 50 °C and stirred for 16 hours at 50 °C. The mixture was then cooled to room temperature and diluted with Et_2O (10 mL) before passing through a plug of silica. The plug was washed with an additional 15 mL of Et_2O and the solvents were removed in vacuo. The crude product **4q** was charged to the next step without further purification.

30%wt. H_2O_2 (1 mL) was added to a solution of pinacol boronic ester **4q** (1 mmol, 1.0 equiv) and 2N aq. NaOH (1.5 ml) in THF (4 mL) at 0 °C. The reaction mixture was slowly allowed to warm to ambient temperature. After 3h, the mixture was diluted with EtOAc (5 mL). The organic layer was separated and the aqueous layer was extracted with EtOAc (2 × 5 mL). The combined organic layers were dried over anhydrous Na_2SO_4, filtered, and the solvent was removed in vacuo. The crude alcohol was charged to the next step without further purification.

A solution of alcohol obtained above (0.2 mmol) in TFA (0.5 mL) and H_2O (0.5 mL) was stirred at room temperature. After 2h, the reaction mixture was concentrated under reduced pressure. Purification by flash chromatography (3% MeOH/EtOAc to 15% MeOH/EtOAc) afforded compound **10** as an off-white solid (43% yield over 3 steps) as a single diastereomer (dr >20:1).

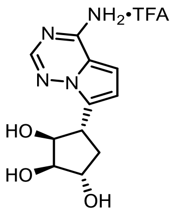

(1S,2R,3S,4S)-4-(4-aminopyrrolo[2,1-f][1,2,4]triazin-7-yl)cyclopentane-1,2,3-triol TFA Salt (10): **^{1}H NMR** (CD_3OD, 400 MHz): δ (ppm) 7.75 (s, 1H), 6.86 (d, *J* = 4.5 Hz, 1H), 6.58 (d, *J* = 4.5 Hz, 1H), 4.38 (dd, *J* = 7.6, 5.3 Hz, 1H), 4.13 (ddd, *J* = 7.1, 5.5, 3.6 Hz, 1H), 3.96 (dd, *J* = 5.3, 3.6 Hz, 1H), 3.69 (q, *J* = 8.8 Hz, 1H), 2.64 (ddd, *J* = 13.9, 9.2, 7.1 Hz, 1H), 1.71 (ddd, *J* = 13.9, 9.2, 5.5 Hz, 1H). **^{13}C NMR** (101 MHz, CD_3OD): δ (ppm) 147.6, 134.6, 115.4, 109.8, 103.1, 79.6, 77.2, 76.7, 49.8, 40.8, 36.5. **^{19}F NMR** (376 MHz, CD_3OD) δ –77.1. **$[\alpha]^{25}_D$** = –6.2 (c = 1.0, CH_3OH).

HRMS (ESI): m/z calcd for $C_{11}H_{15}O_3N_4^+$ [M – TFA + H]$^+$ 251.1139 found 251.1139.

IR (ν_{max}/cm^{-1}) 3349, 2361, 2341, 1679, 1525, 1447, 1141, 1045, 847, 802, 726, 669.

6. Synthesis of Benzo-Imidazole Derived CC-Ns

(1R,2S,3S,5R)-3-(1H-benzo[d]imidazol-2-yl)-5-(hydroxymethyl)cyclopentane-1,2-diol (13):

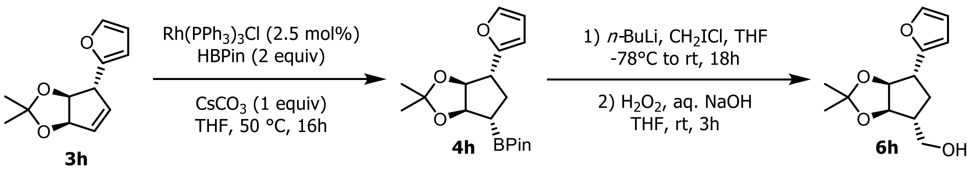

The hydroboration-homologation-oxidation sequence were performed starting with 4 mmol scale using the above procedure (**3h** to **6h**) and the crude alcohol (**6h**) was used directly for the next step.

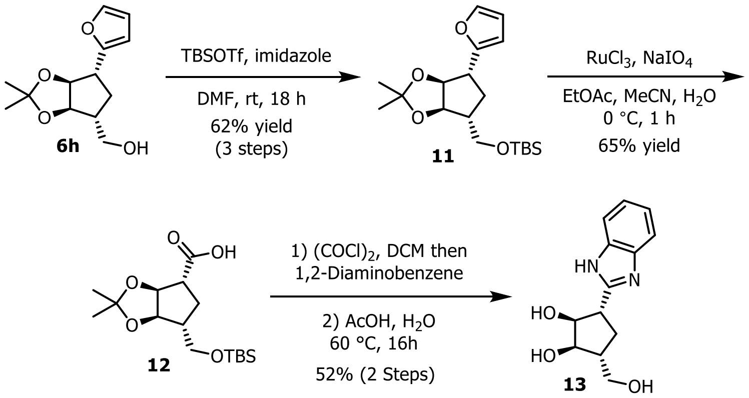

TBSOTf (525 µL, 2.25 mmol, 1.5 equiv) was added dropwise to a solution of alcohol **6h** (1.5 mmol, 1.0 equiv), imidiazole (153 mg, 2.25 mmol, 1.5 equiv) and DMAP (18.3 mg, 0.15 mmol, 0.1 equiv) in DCM (6 mL) and stirred at ambient temperature over 4 h. The reaction mixture was diluted with DCM (7 mL) and the organic layer was washed with an aq. sat. solution of NH_4Cl (2 x 7 mL) and dried over Na_2SO_4, filtered, and the solvent was removed in vacuo. Purification by flash chromatography (5% Et_2O/petrol to 15% Et_2O/petrol) afforded compound **11** as a colourless oil (347 mg, 62% yield over 3 steps) as a single diastereomer (dr >20:1).

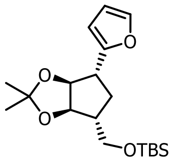

(((3aR,4R,6R,6aS)-6-(furan-2-yl)-2,2-dimethyltetrahydro-4H-cyclopenta[d][1,3]dioxol-4-yl)methoxy)tert-butyl-dimethylsilane (11): **^{1}H NMR** (400 MHz, $CDCl_3$): δ (ppm) 7.33 (dd, J = 1.8, 0.9 Hz, 1H), 6.33 – 6.23 (m, 1H), 6.07 (dd, J = 3.2, 0.9 Hz, 1H), 4.59 (t, J = 6.4 Hz, 1H),

4.46 (dd, J = 6.9, 4.5 Hz, 1H), 3.64 (qd, J = 10.1, 5.4 Hz, 3H), 3.26 (dt, J = 11.8, 6.4 Hz, 1H), 2.38 – 2.07 (m, 3H), 1.83 (ddd, J = 13.0, 11.3, 9.9 Hz, 1H), 1.54 (s, 3H), 1.32 (s, 3H), 0.89 (s, 9H), 0.04 (s, 6H). **^{13}C NMR** (101 MHz, $CDCl_3$) δ 156.3, 141.6, 112.7, 110.2, 104.7, 85.0, 82.4, 63.8, 47.5, 44.9, 32.9, 27.8, 26.0, 25.2, 18.4, -5.26, -5.28. **$[\alpha]^{25}_D$** = –18.2 (c = 1.0, $CHCl_3$).

HRMS (ESI): m/z calcd for $C_{19}H_{32}O_4SiNa^+$ $[M + Na]^+$ 375.1962 found 375.1964.

IR (ν_{max}/cm^{-1}) 2987, 2954, 2931, 2896, 2858, 2360, 2342, 1541, 1507, 1472, 1381, 1372, 1255, 1210, 1162, 1149, 1097, 1066, 1007, 940, 919, 867, 837, 813, 780, 730.

A round bottom flask containing $RuCl_3$ hydrate (26.2 mg, 0.1 mmol, 10 mol%) and $NaIO_4$ (3.85 g, 18 mmol, 18 equiv) in EtOAc:H_2O:MeCN (8 mL:10 mL:1 mL), a solution of furan **S-1** (1 mmol, 1.0 equiv) in EtOAc (2 ml) was added to dropwise over 30 min then mixture was stirred vigorously at room temperature. After 30 min, reaction was quenched with H_2O (15 mL) followed by EtOAc (15 mL) and the organic layer was washed with an aq. sat. solution of NaCl (2 x 10 mL) and dried over Na_2SO_4, filtered, and the solvent was removed in vacuo. Purification by flash chromatography (100% CH_2Cl_2 to 5% MeOH/CH_2Cl_2) afforded acid **12** as a brown colored oil (182 mg, 65% yield) as a single diastereomer (dr >20:1).

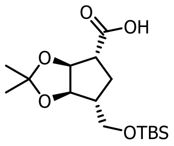

(3aS,4R,6R,6aR)-6-(((tert-butyldimethylsilyl)oxy)methyl)-2,2-dimethyltetrahydro-4H-cyclopenta[d][1,3]dioxole-4-carboxylic acid (12): ^{1}H NMR (400 MHz, $CDCl_3$): δ (ppm) 4.81 (dd, J = 6.6, 5.0 Hz, 1H), 4.45 (dd, J = 6.6, 3.3 Hz, 1H), 3.62 (d, J = 5.0 Hz, 2H), 2.94 (ddd, J = 9.5, 7.6, 5.0 Hz, 1H), 2.37 – 2.20 (m, 2H), 1.95 – 1.77 (m, 1H), 1.50(s, 3H), 1.32 (s, 3H), 0.88 (s, 9H), 0.04 (d, J = 1.8 Hz, 6H). **^{13}C NMR** (101 MHz, $CDCl_3$) δ (ppm) 179.8, 112.5, 83.0, 82.6, 63.7, 50.5, 47.5, 31.0, 27.6, 26.0, 25.1, 18.5, -5.32, -5.34. **$[\alpha]^{25}_D$** = –9.6 (c = 1.0, $CHCl_3$).

HRMS (ESI): m/z calcd for $C_{16}H_{30}O_5SiNa^+$ $[M + Na]^+$ 353.1755 found 353.1754.

IR (ν_{max}/cm^{-1}) 3103, 2989, 2953, 2932, 2858, 2360, 2342, 1735, 1708, 1559, 1472, 1421, 1381, 1256, 1211, 1162, 1099, 1063, 1005, 939, 909, 838, 813, 782, 758.

To a solution of acid **12** (66 mg, 0.2 mmol, 1.0 equiv) and Dimethylformamide (5 µL, cat.) in DCM (2 mL), oxalyl chloride (35 µL, 0.4 mmol, 2.0 equiv) was added and stirred at 50 °C. After 30 min, the reaction mixture was concentrated under vacuum to remove excess oxalyl

chloride. The resulting yellow solid was redissolved in DCM (1 mL) and the solution was added to the flask containing a solution of o-phenylenediamine (33 mg, 0.3 mmol, 1.5 equiv) in DCM (1 mL) at 0 °C then the reaction mixture was allowed to warmed to room temperature. After 3 hours the mixture was diluted with DCM (3 mL) and H_2O (5 mL). The organic layer was extracted, washed with aq. $NaHCO_3$ (2 x 5 mL) followed by 1M HCl (2 x 5 mL) and dried over Na_2SO_4, filtered, and the solvent was removed in vacuo. The crude amide was charged to the next step.

A solution of amide obtained above (0.2 mmol) in AcOH (0.5 mL) and H_2O (0.5 mL) was stirred at 60 °C for 16 h. The reaction mixture was concentrated under reduced pressure and purification by flash chromatography (5% MeOH/EtOAc to 30% MeOH/EtOAc) afforded compound **13** as a light-yellow oil (77% yield over 3 steps) as a single diastereomer (dr >20:1).

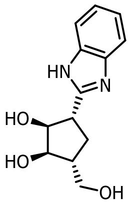

(1R,2S,3S,5R)-3-(1H-benzo[d]imidazol-2-yl)-5-(hydroxymethyl)cyclopentane-1,2-diol (13): ^{1}**H NMR** (500 MHz, CD_3OD): δ (ppm) 7.53 (dd, *J* = 6.1, 3.2 Hz, 2H), 7.22 (dd, *J* = 6.1, 3.2 Hz, 2H), 4.22 (dd, *J* = 8.4, 5.3 Hz, 1H), 4.00 (dd, *J* = 5.3, 3.8 Hz, 1H), 3.47 (dt, *J* = 10.8, 8.4 Hz, 1H), 2.36 (dt, *J* = 13.0, 8.4 Hz, 1H), 2.30 – 2.19 (m, 1H), 1.72 (ddd, *J* = 13.0, 10.8, 8.4 Hz, 1H). ^{13}**C NMR** (101 MHz, CD_3OD) δ (ppm) 157.8, 138.9, 123.6, 115.3, 78.6, 75.4, 64.8, 48.2, 45.0, 30.6. **$[\alpha]^{25}_{D}$** = –16.8 (c = 1.0, CH_3OH).

HRMS (APCI): m/z calcd for $C_{13}H_{17}N_2O_3^+$ [M + H]$^+$ 249.1234 found 249.1234.

IR (ν_{max}/cm^{-1}) 3401, 2919, 2360, 2342, 1682, 1541, 1437, 1204, 1139, 842, 803, 724, 699.

7. Synthesis of Carbocyclic Showdomycin 16

3-((1S,2S,3R,4R)-2,3-dihydroxy-4-(hydroxymethyl) cyclopentyl)-1H-pyrrole-2,5-dione (16):

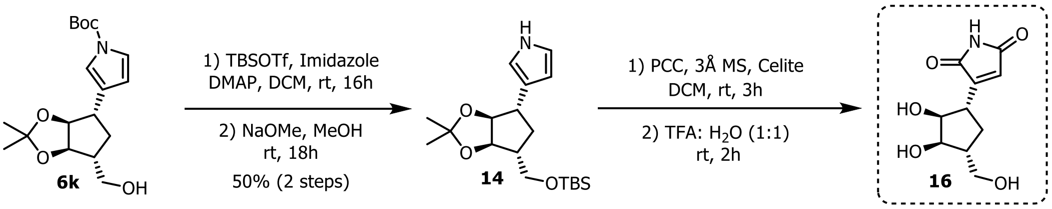

TBSOTf (350 µL, 1.5 mmol, 1.5 equiv) was added dropwise to a solution of alcohol **7o** (1 mmol, 1.0 equiv), imidiazole (102 mg, 1.5 mmol, 1.5 equiv) and DMAP (12 mg, 0.1 mmol, 0.1 equiv) in DCM (4 mL) and stirred at ambient temperature over 12 h. The reaction mixture was diluted with DCM (5 mL) and the organic layer was washed with an aq. sat. solution of NH_4Cl (2 x 5 mL) and dried over Na_2SO_4, filtered, and the solvent was removed in vacuo. The crude TBS alcohol was charged to the next step without further purification.

NaOMe (25 wt.% in MeOH, 2.3 mL, 10 mmol, 10 equiv) was added dropwise to a solution of TBS alcohol obtained above (1 mmol, 1.0 equiv) in MeOH (2 mL) and stirred at ambient temperature. After 16 h, the mixture was diluted with Et_2O (15 mL) and water (10 mL). The organic layer was separated, and the aqueous layer was extracted with Et_2O (2 × 7 mL). The combined organic layers were dried over anhydrous Na_2SO_4, filtered, and the solvent was removed in vacuo. Purification by flash chromatography (15% Et_2O/petrol to 35% Et_2O/petrol) afforded compound **14** as a colourless oil (65% yield over 2 steps) as a single diastereomer (dr >20:1).

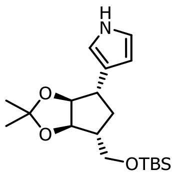

3-((3aS,4S,6R,6aR)-6-(((tert-butyldimethylsilyl)oxy)methyl)-2,2-dimethyl tetrahydro-4H-cyclopenta[d][1,3]dioxol-4-yl)-1H-pyrrole (14): ^{1}**H NMR** ($CDCl_3$, 400 MHz): δ (ppm) 6.74 (td, J = 2.7, 1.9 Hz, 1H), 6.65 (dtd, J = 2.7, 1.7, 0.7 Hz, 1H), 6.15 (td, J = 2.7, 1.7 Hz, 1H), 4.47 – 4.42 (m, 2H), 3.68 (qd, J = 10.0, 5.2 Hz, 2H), 3.21 – 3.07 (m, 1H), 2.33 – 2.17 (m, 2H), 1.72 (td, J = 12.0, 10.3 Hz, 1H), 1.54 (s, 3H), 1.32 (s, 3H), 0.90 (s, 9H), 0.05 (d, J = 1.3 Hz, 6H). ^{13}**C NMR** (101 MHz, $CDCl_3$): δ (ppm) 125.6, 118.2, 114.5, 112.5, 107.4, 87.8, 82.5, 64.1, 47.6, 43.8, 35.5, 27.9, 26.1, 25.4, 18.5, -5.2.

PCC (323 mg, 1.5 mmol, 3 equiv.) was added potion wise (~3 portions) to a suspension of pyrrole **14** (0.5 mmol, 1 equiv), Celite (300 mg) and 3Å MS (300 mg) in DCM (5 mL) and the mixture was stirred vigorously at room temperature. After 3h, the reaction mixture was passed through a plug of celite, washed with DCM (7.5 mL) and filtrate was concentrated under reduced pressure. Purification by flash chromatography (10% Hexane/Et_2O to 30% Hexane/Et_2O) afforded compound **15** with coloured impurities which was charged to next step.

A solution of **15** (0.2 mmol) in TFA (0.5 mL) and H_2O (0.5 mL) was stirred at room temperature. After 2h, the reaction mixture was concentrated under reduced pressure. Purification by flash chromatography (1% MeOH/EtOAc to 10% MeOH/EtOAc) afforded compound **16** as off-white solid (53% yield over 2 steps) as a single diastereomer (dr >20:1).

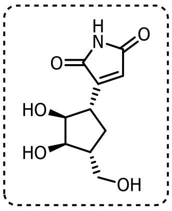

3-((1S,2S,3R,4R)-2,3-dihydroxy-4-(hydroxymethyl)cyclopentyl)-1H-pyrrole-2,5-dione (16): ^{1}**H NMR** (CD_3OD, 400 MHz): δ (ppm) 6.45 (d, *J* = 1.3 Hz, 1H), 4.04 (dd, *J* = 8.2, 5.3 Hz, 1H), 3.92 (dd, *J* = 5.3, 3.8 Hz, 1H), 3.66 – 3.52 (m, 2H), 3.17 – 3.01 (m, 1H), 2.32 – 2.13 (m, 2H), 1.49 – 1.33 (m, 1H). ^{13}**C NMR** (101 MHz, CD_3OD): δ (ppm) 174.1, 173.6, 153.0, 128.2, 77.7, 75.3, 64.6, 48.0, 42.2, 30.7. $[\alpha]^{25}_{D}$ = −53.9 (c = 1.0, CH_3OH).

HRMS (ESI): m/z calcd for $C_{10}H_{13}O_5NNa^+$ $[M + Na]^+$ 250.0686 found 250.0686.

IR (ν_{max}/cm^{-1}) 3365, 2921, 2848, 2360, 2342, 1718, 1653, 1457, 1203, 1113, 1021, 688.

8. Supplementary References

(1) Goetzke, F. W., Mortimore, M. & Fletcher, S. P. Enantio- and Diastereoselective Suzuki–Miyaura Coupling with Racemic Bicycles. *Angew. Chemie - Int. Ed.* **58**, 12128–12132 (2019).

(2) Kučera, R., Goetzke, F. W. & Fletcher, S. P. An Asymmetric Suzuki-Miyaura Approach to Prostaglandins: Synthesis of Tafluprost. *Org. Lett.* **22**, 2991–2994 (2020).

(3) Pereira, S. & Srebnik, M. Transition metal-catalyzed hydroboration of and CCl4 addition to alkenes. *J. Am. Chem. Soc.* **118**, 909–910 (1996).

(4) Maier, L. *et al.* Diastereoselective Flexible Synthesis of Carbocyclic C-Nucleosides. *J. Org. Chem.* **82**, 3382–3402 (2017).
