# Supplementary material for: Catalytic asymmetric synthesis of carbocyclic C-nucleosides
Source: Commun Chem. 2022 Nov 19;5:154. doi: 10.1038/s42004-022-00773-6 (PMC9676730; doi:10.1038/s42004-022-00773-6)
Supplement: Supplementary file 2 — Description of Additional Supplementary Files [file 42004_2022_773_MOESM2_ESM.pdf]

Description of Additional Supplementary Files

File name: Supplementary Data 1

Description: Supplementary Information containing NMR Spectra.
