# Supplementary material for: Catalytic asymmetric synthesis of carbocyclic C-nucleosides
Source: Commun Chem. 2022 Nov 19;5:154. doi: 10.1038/s42004-022-00773-6 (PMC9676730; doi:10.1038/s42004-022-00773-6)
Supplement: Supplementary file 3 — Supplementary data 1 [file 42004_2022_773_MOESM3_ESM.pdf]

Catalytic Asymmetric Synthesis of Carbocyclic C-Nucleosides

Sourabh Mishra, Florian C. T. Modicom, Conor L. Dean, Stephen P. Fletcher*

Department of Chemistry, Chemistry Research Laboratory, University of Oxford, Oxford OX1 3TA, UK

Supporting Information – NMR Spectra

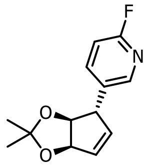

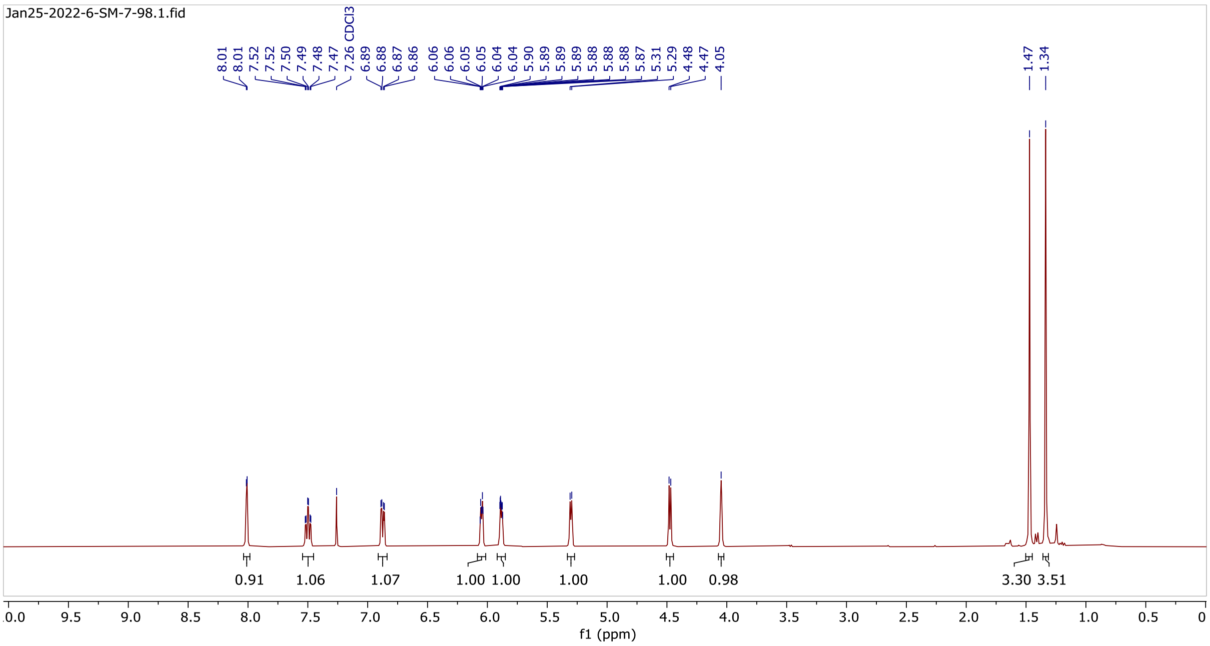

^{1}H NMR spectra of **3c**

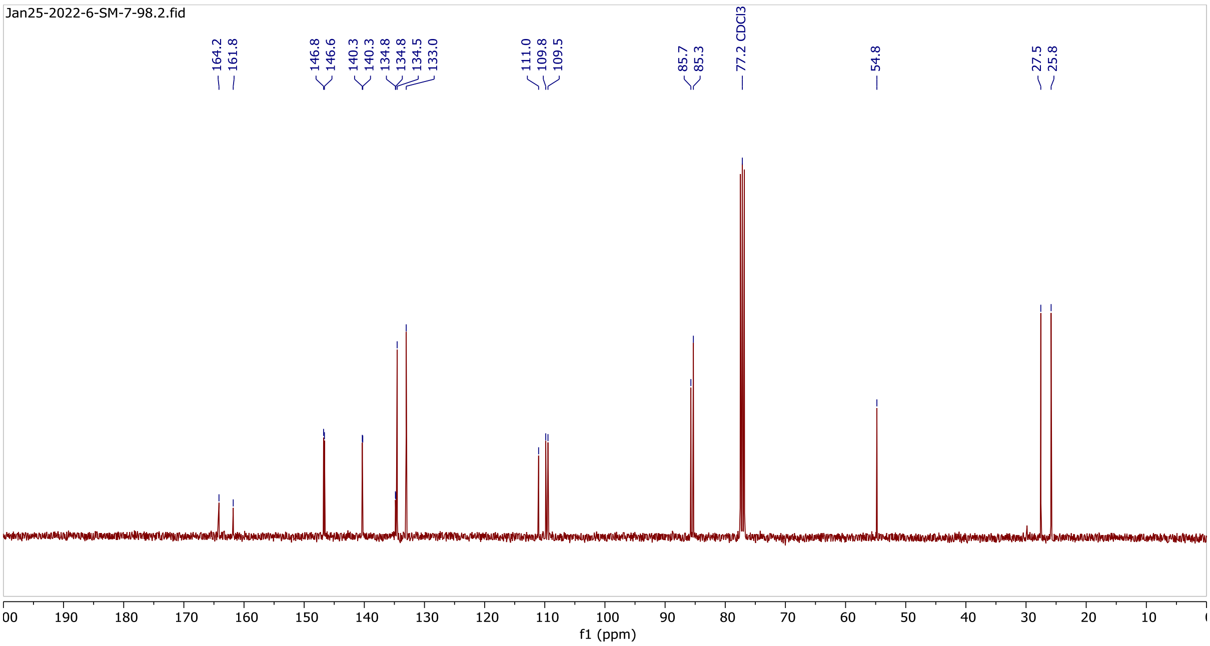

^{13}C NMR spectra of **3c**

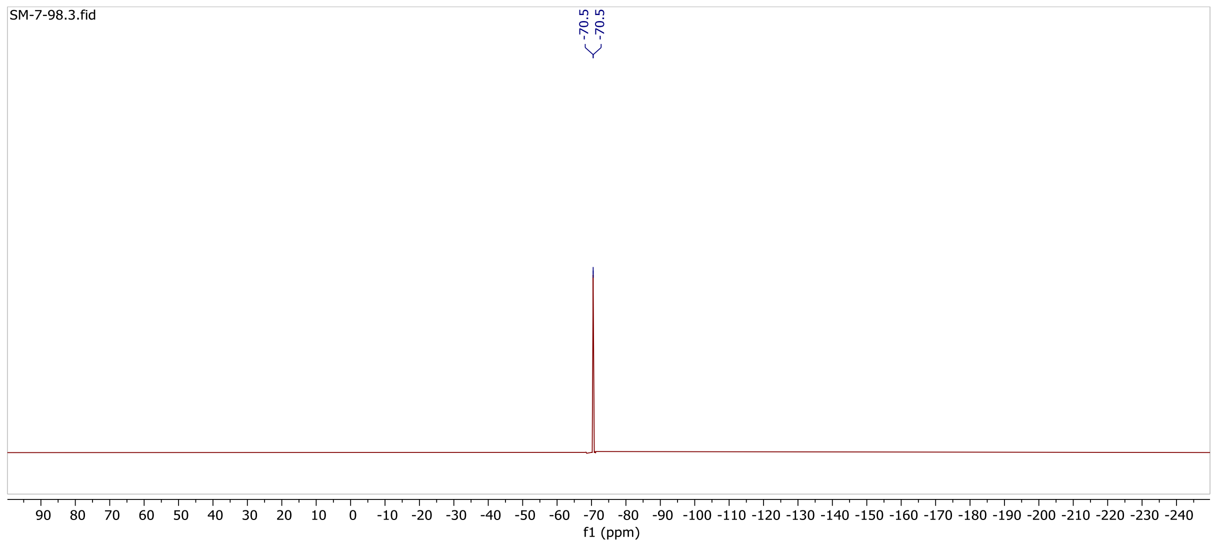

^{19}F NMR spectra of **3c**

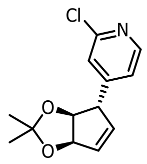

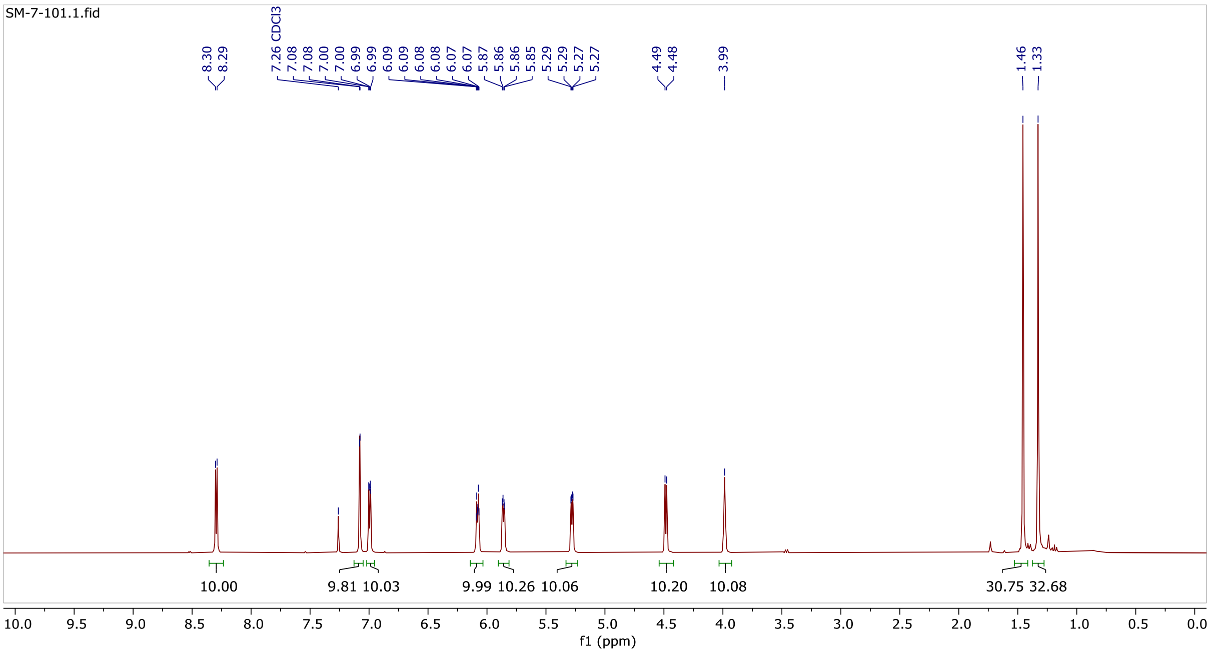

^{1}H NMR spectra of **3d**

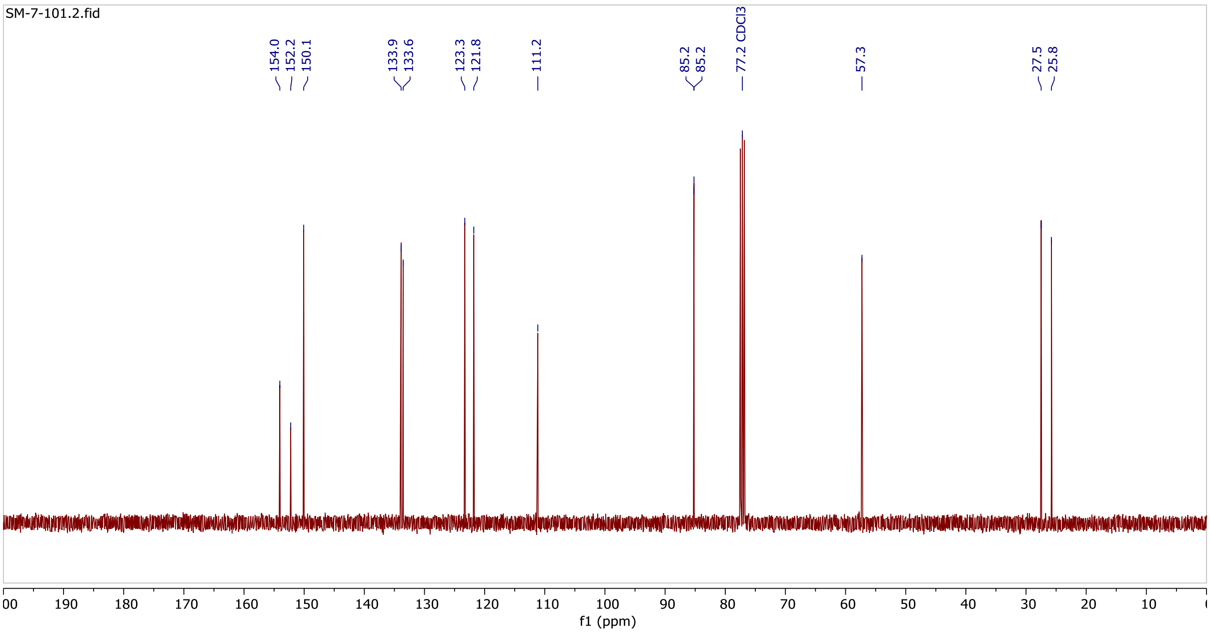

^{13}C NMR spectra of **3d**

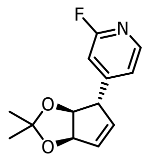

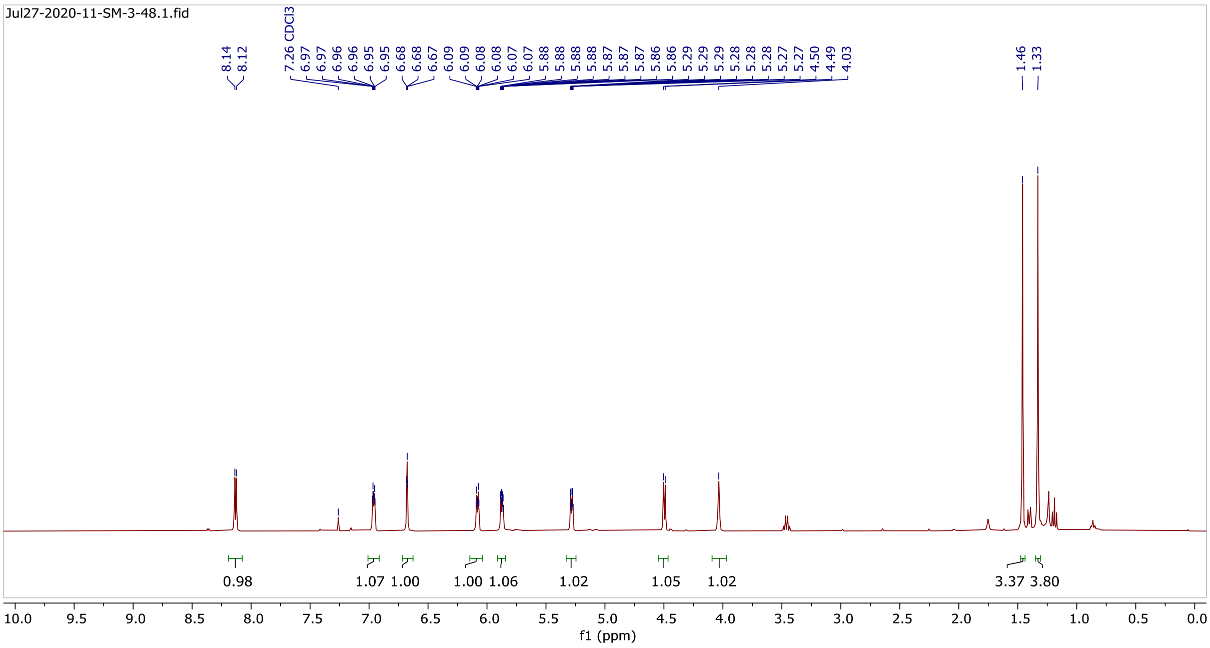

^{1}H NMR spectra of **3e**

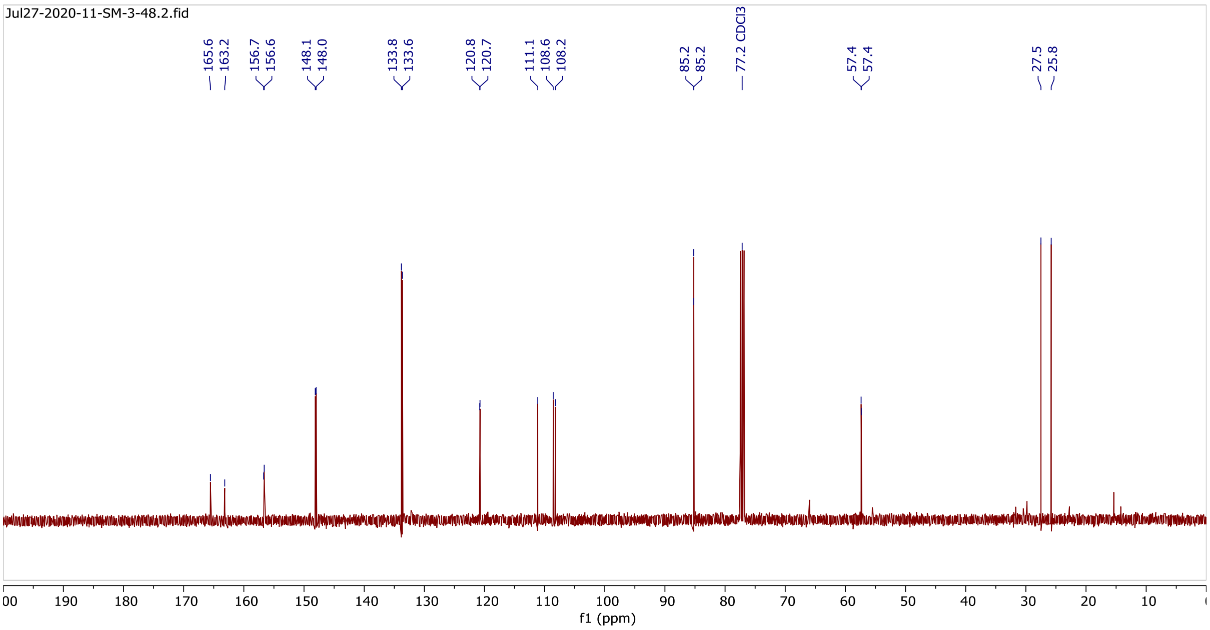

^{13}C NMR spectra of **3e**

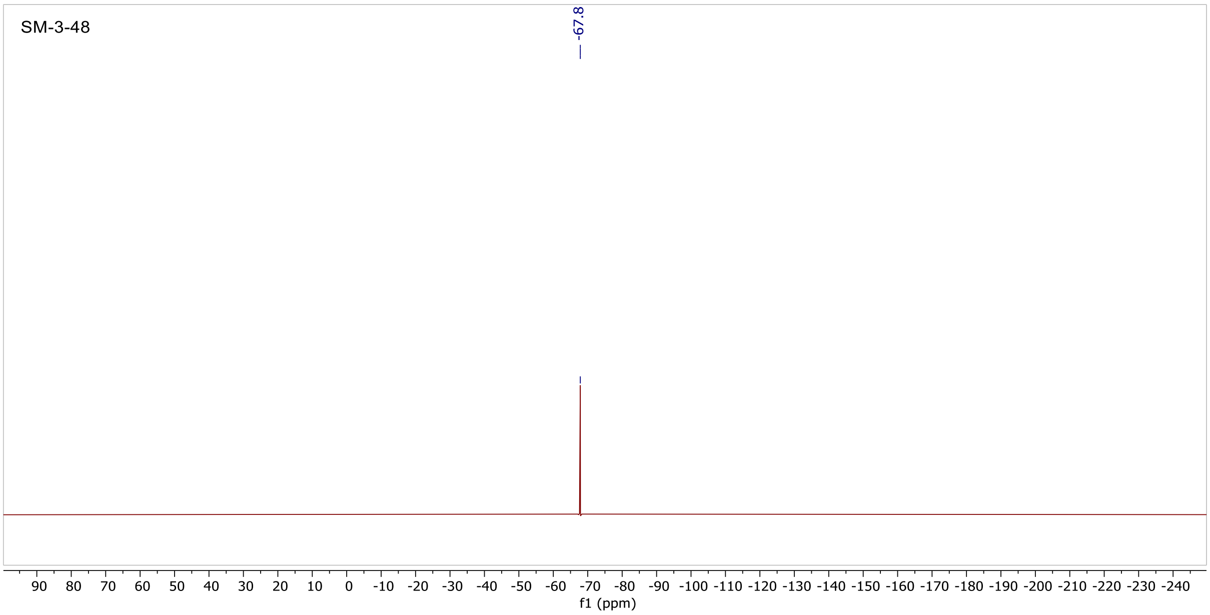

^{19}F NMR spectra of **3e**

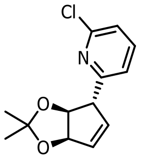

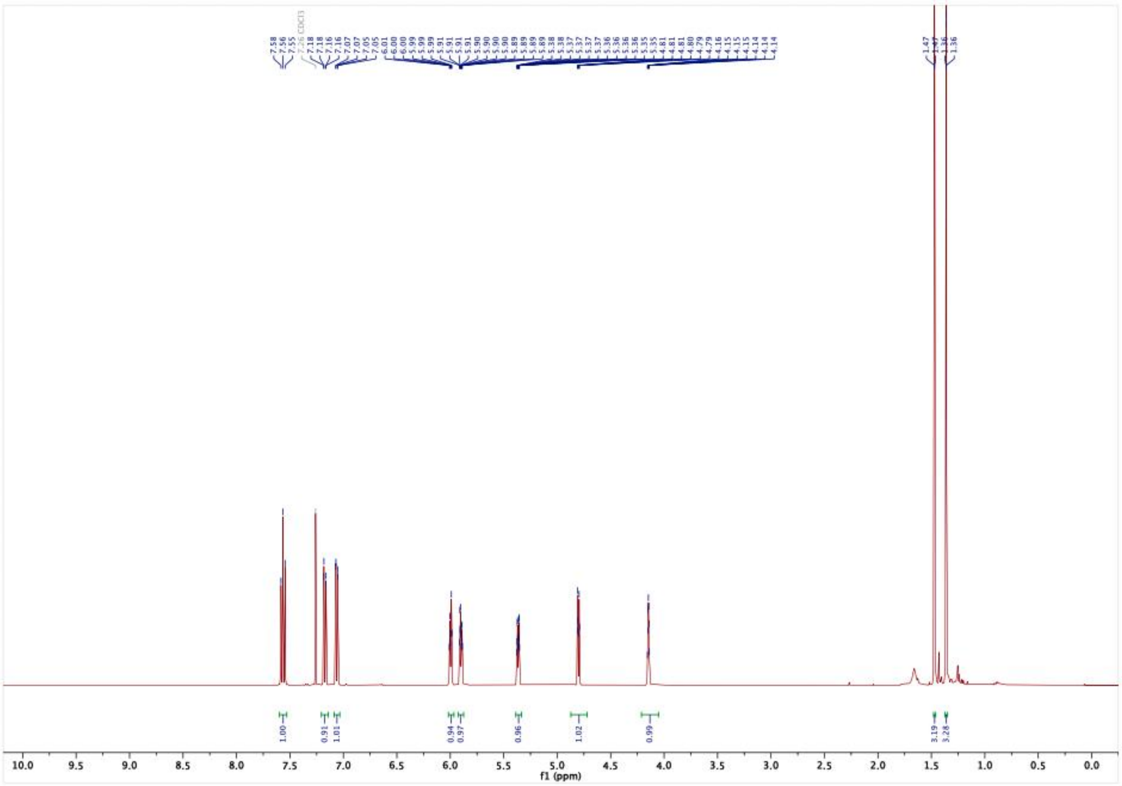

^{1}H NMR spectra of **3f**

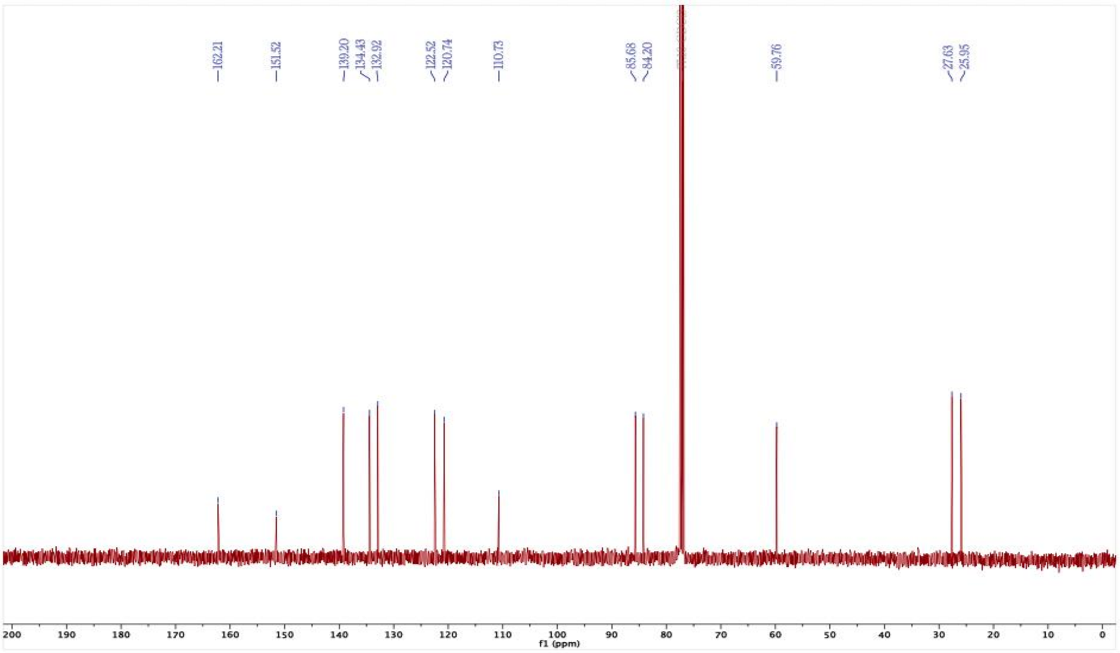

^{13}C NMR spectra of **3f**

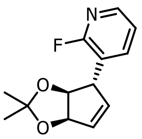

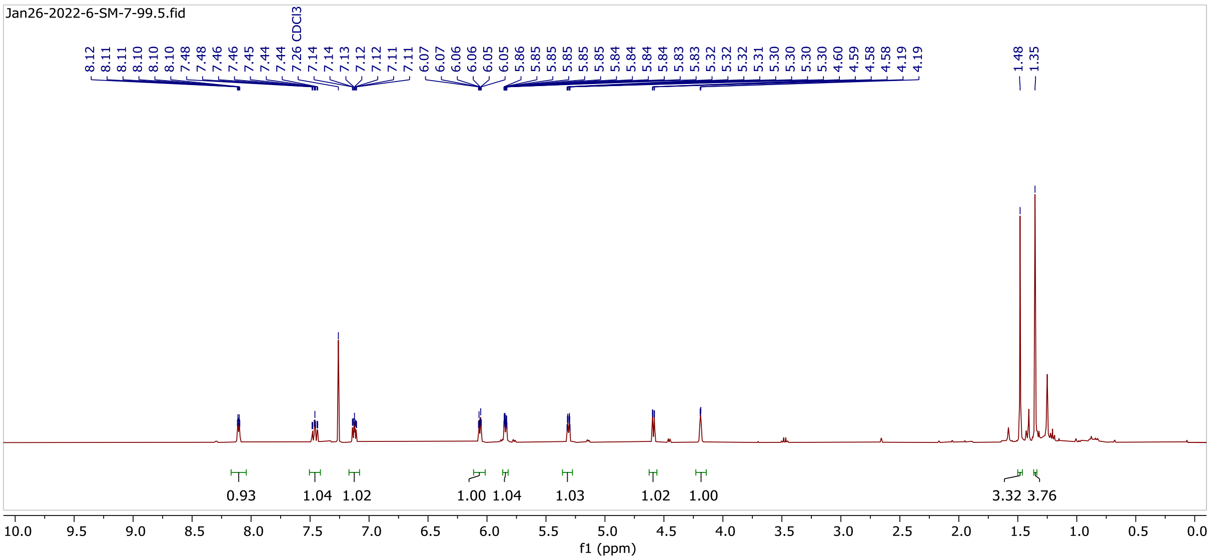

^{1}H NMR spectra of **3g**

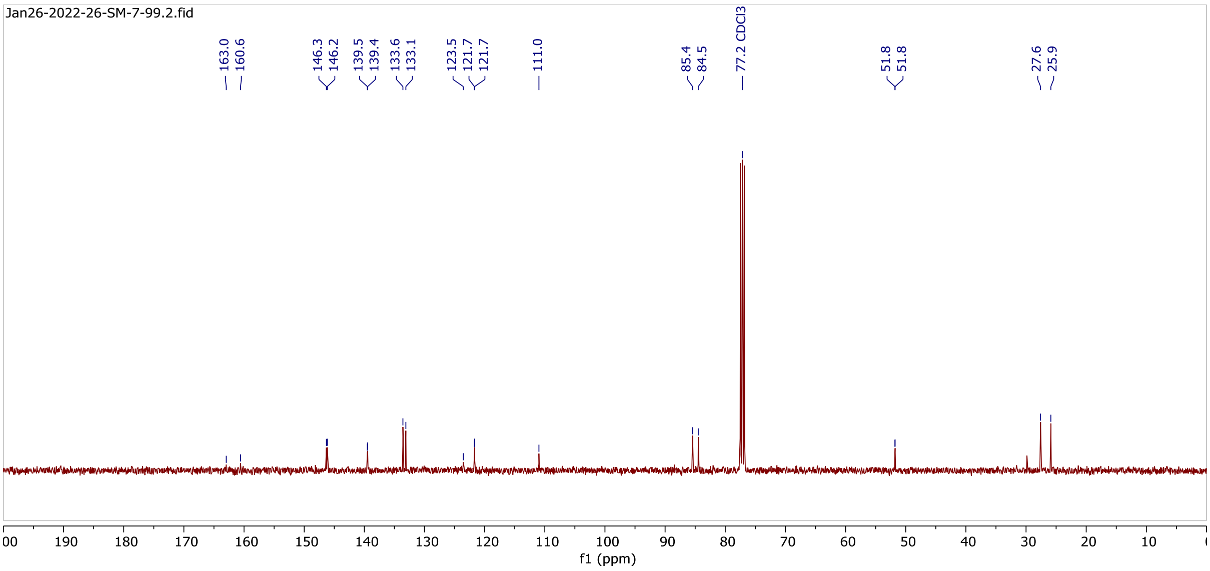

^{13}C NMR spectra of **3g**

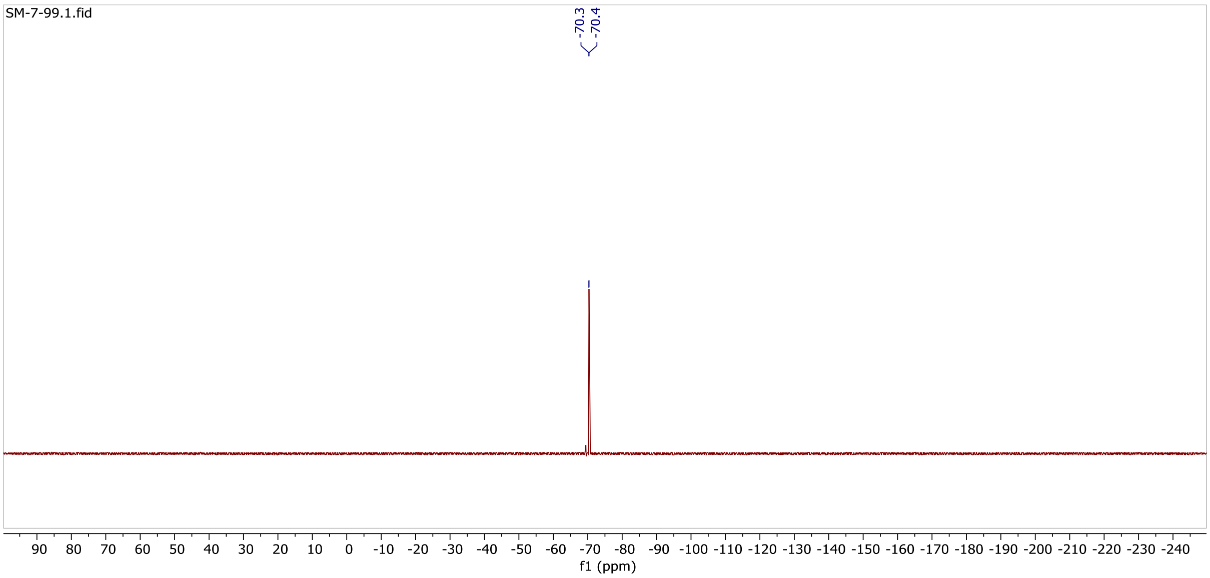

^{19}F NMR spectra of **3g**

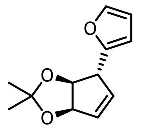

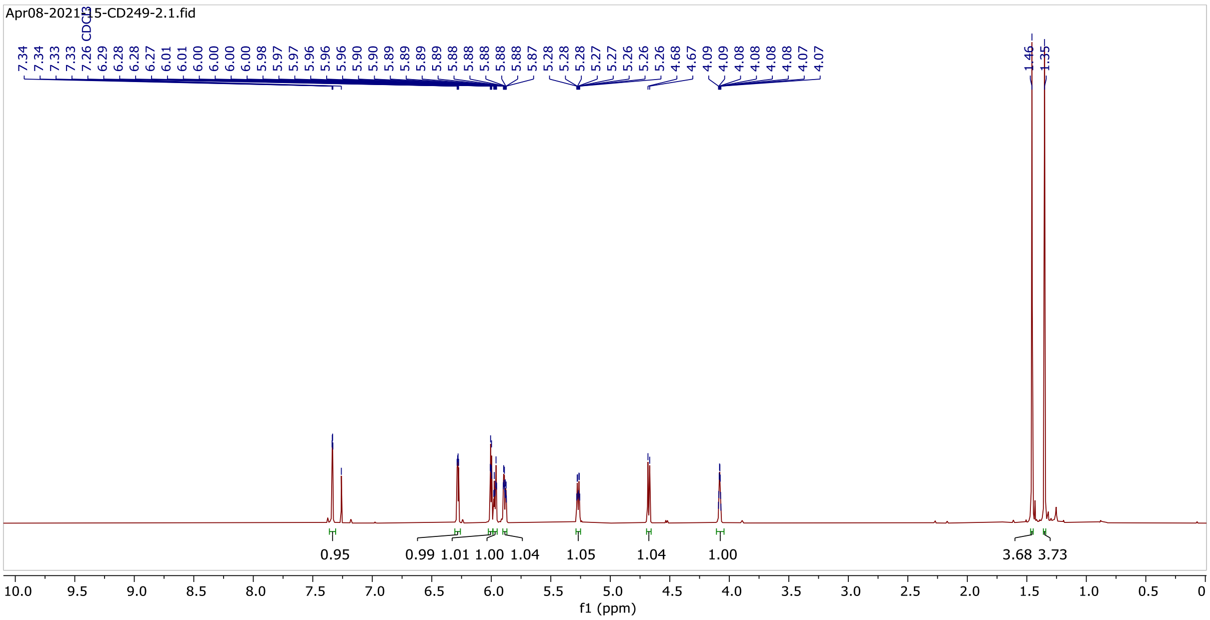

^{1}H NMR spectra of **3h**

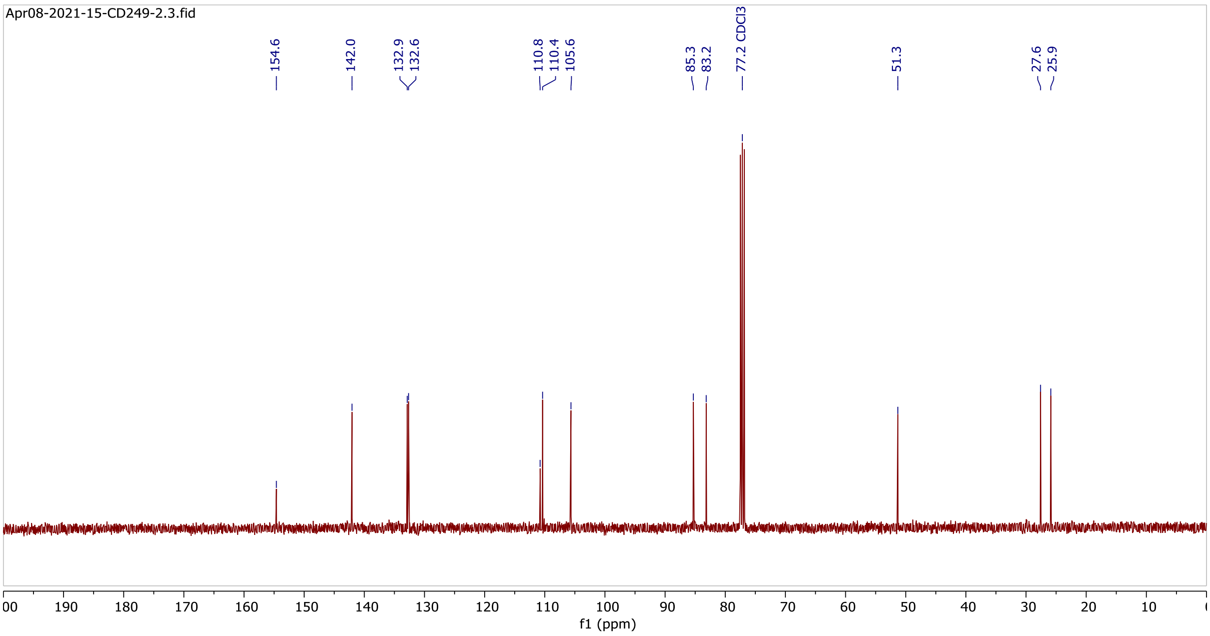

^{13}C NMR spectra of **3h**

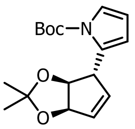

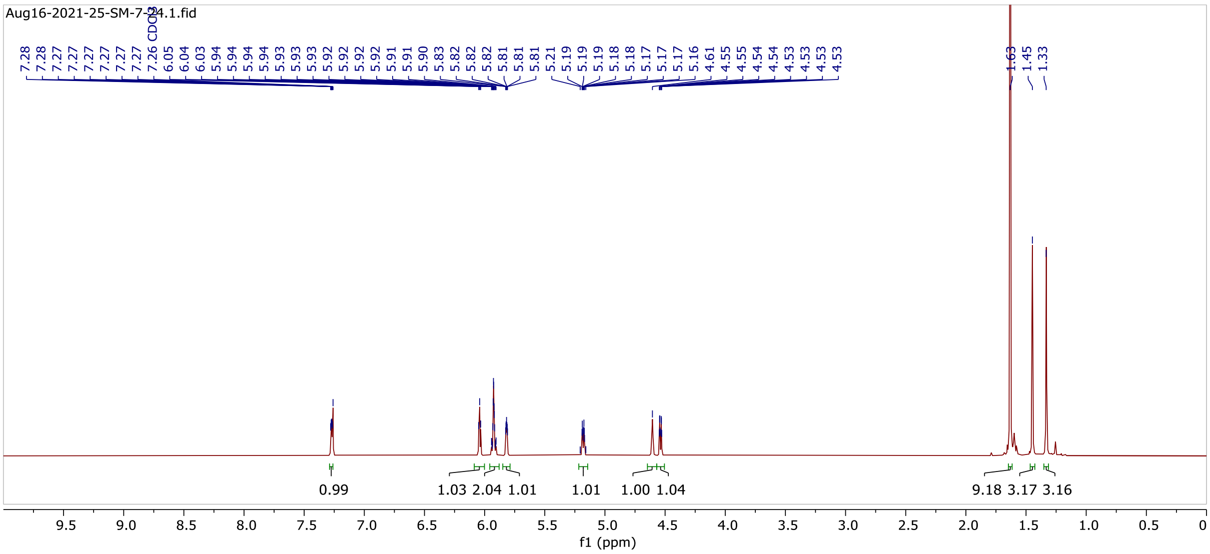

^{1}H NMR spectra of **3j**

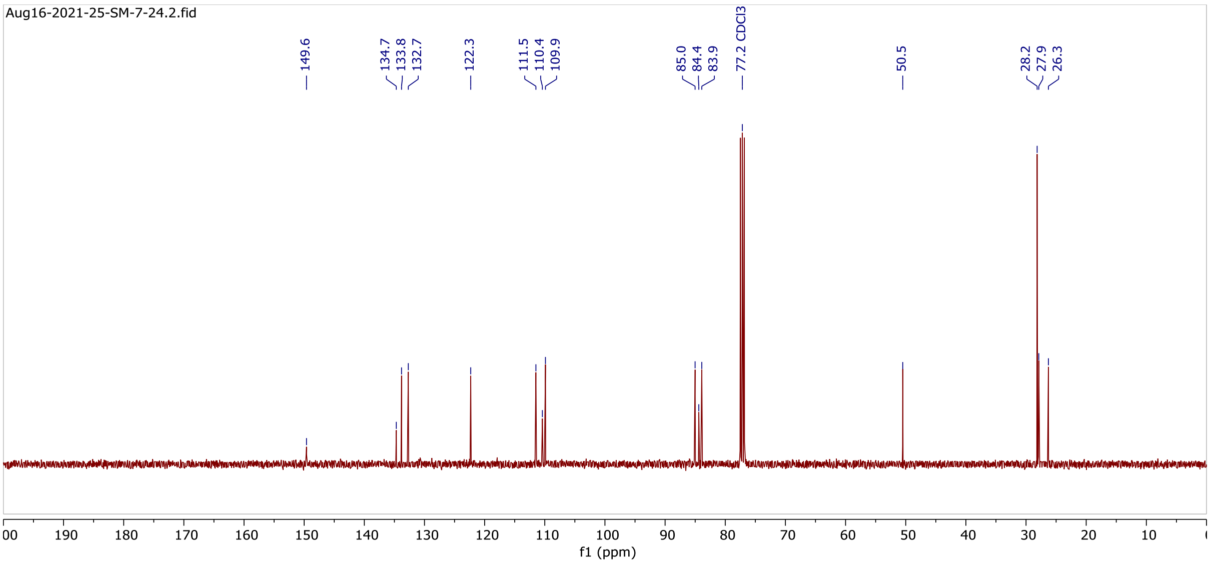

^{13}C NMR spectra of **3j**

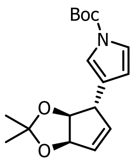

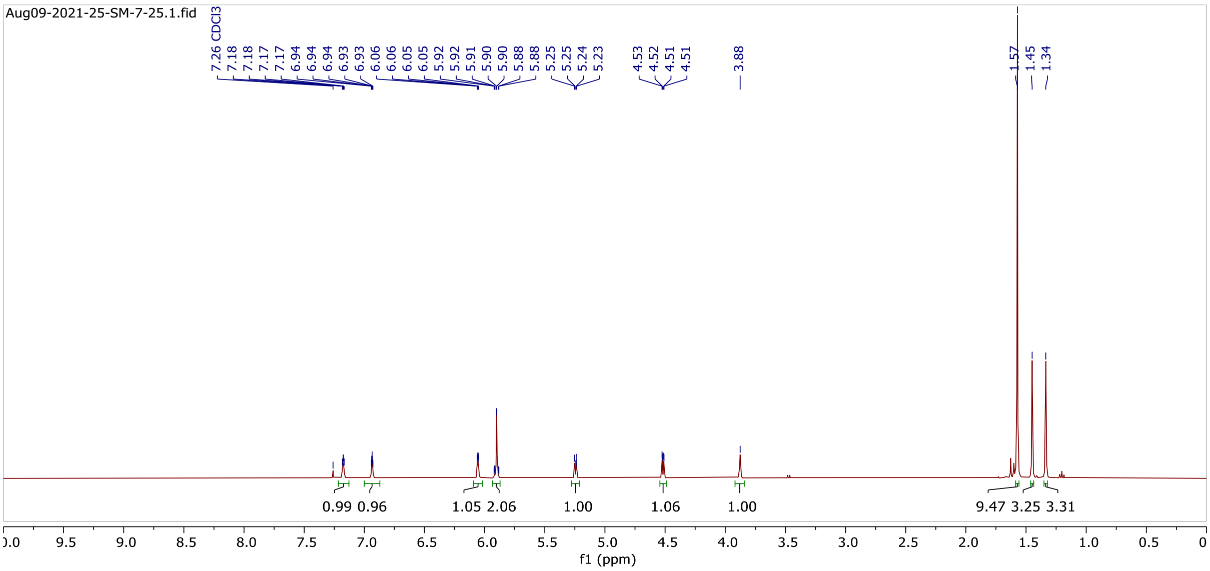

^{1}H NMR spectra of **3k**

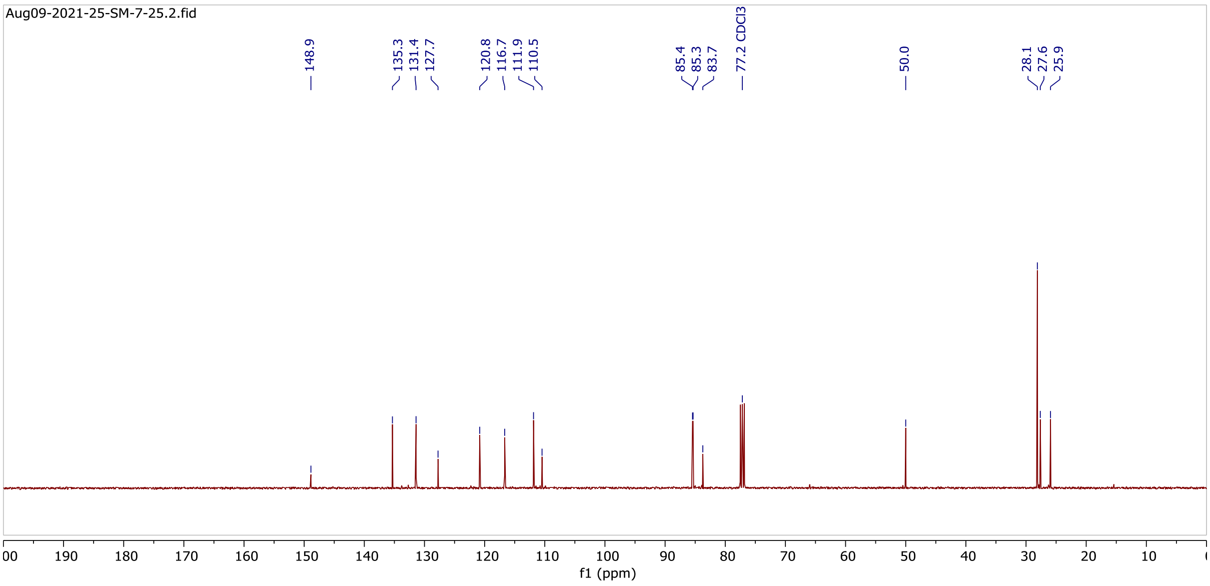

^{13}C NMR spectra of **3k**

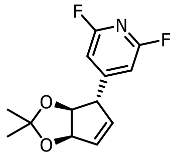

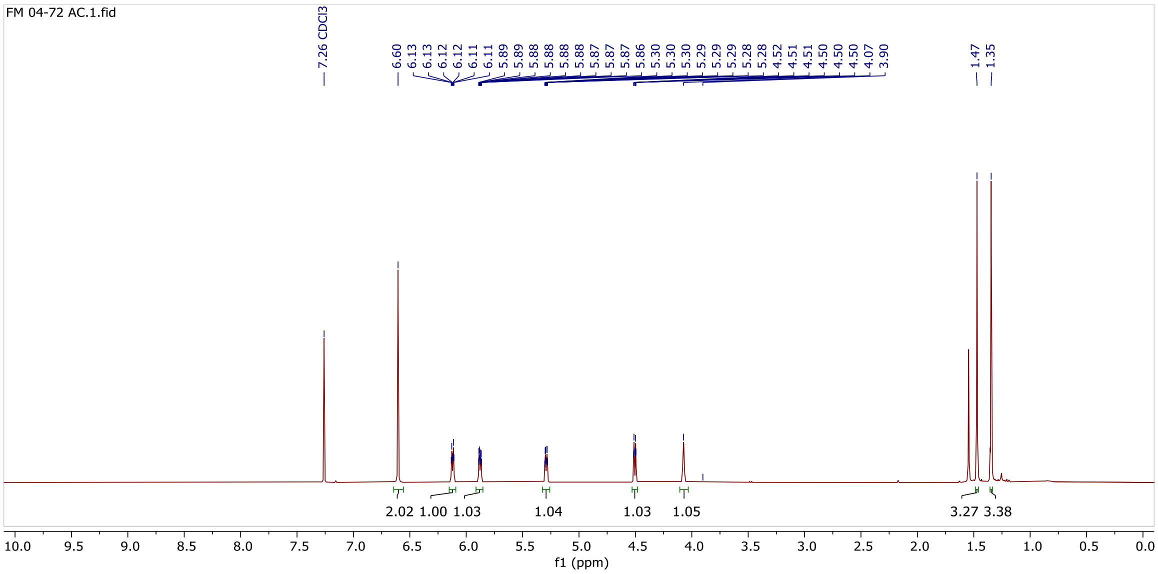

^{1}H NMR spectra of **3l**

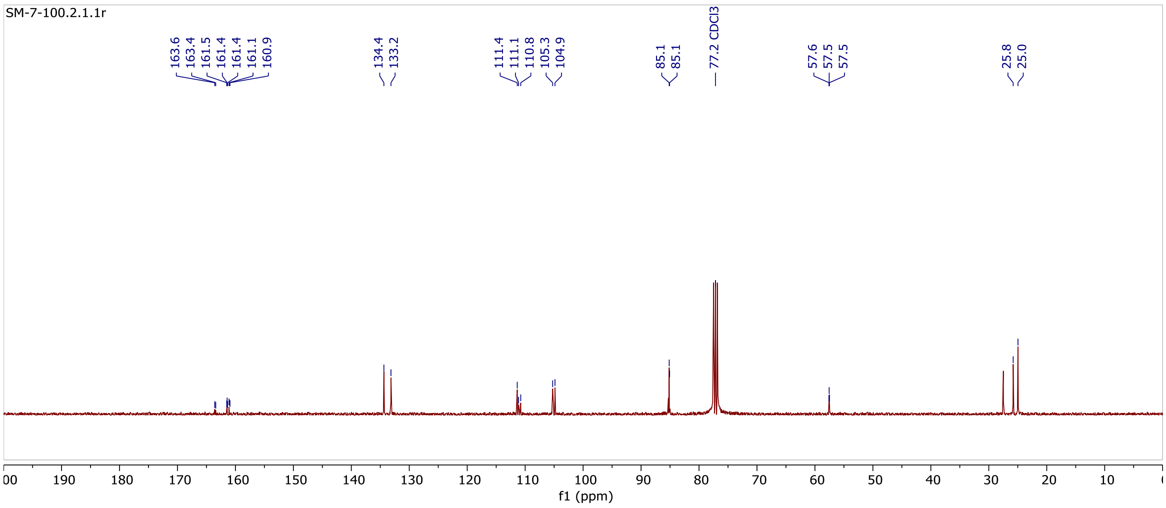

^{13}C NMR spectra of **3l**

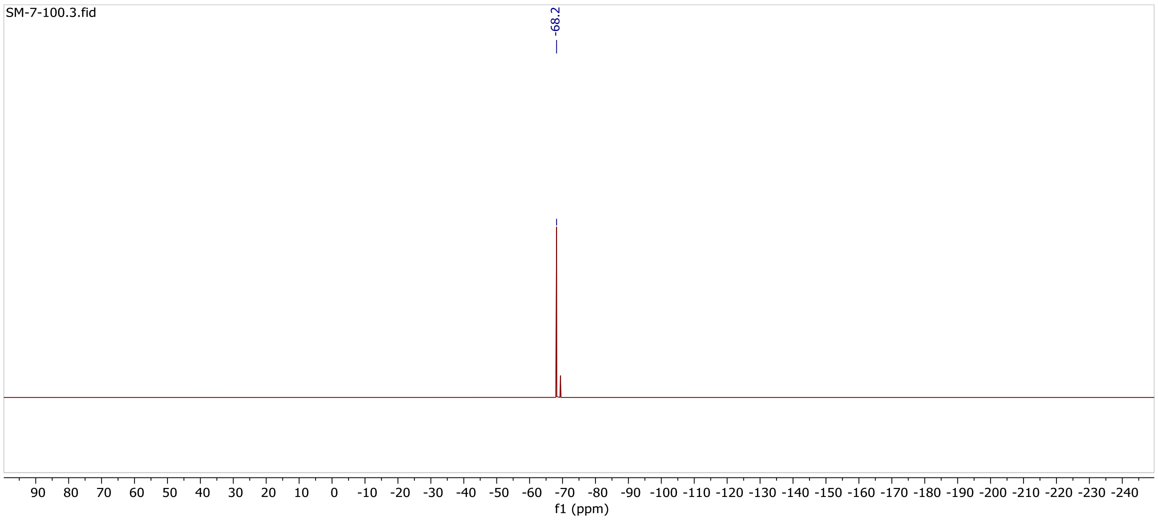

^{19}F NMR spectra of **3l**

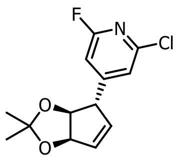

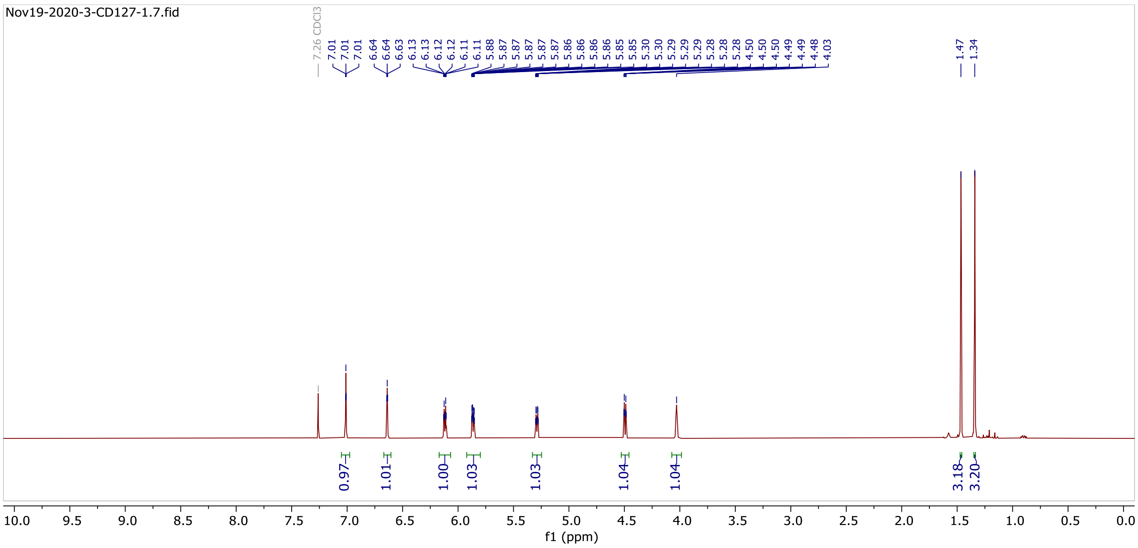

^{1}H NMR spectra of **3m**

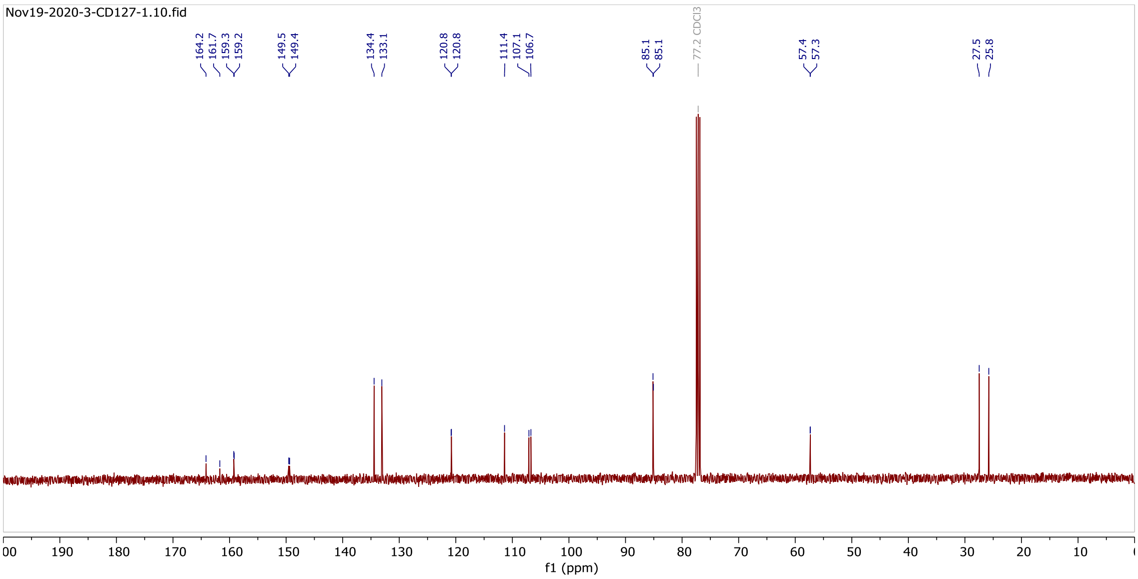

^{13}C NMR spectra of **3m**

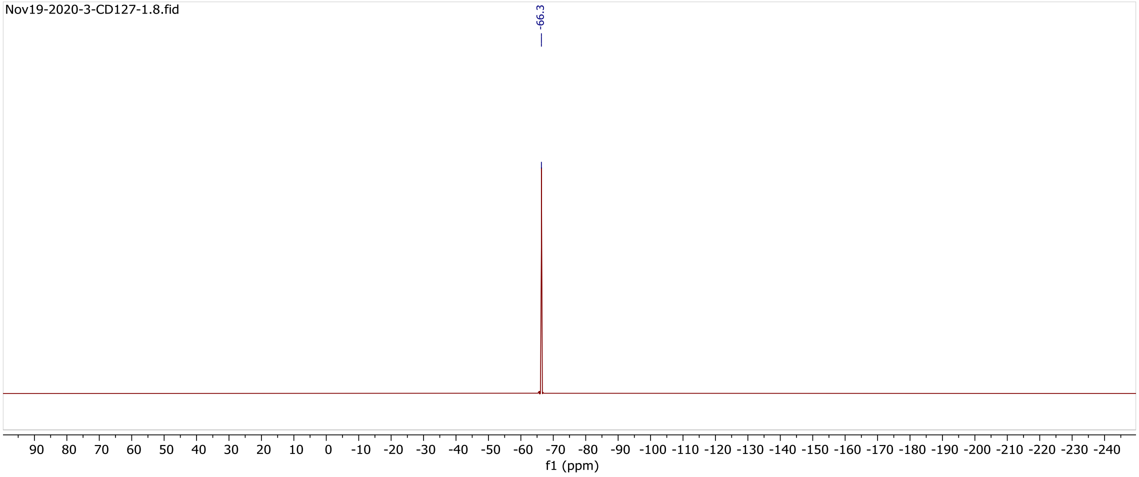

^{19}F NMR spectra of **3m**

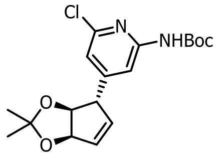

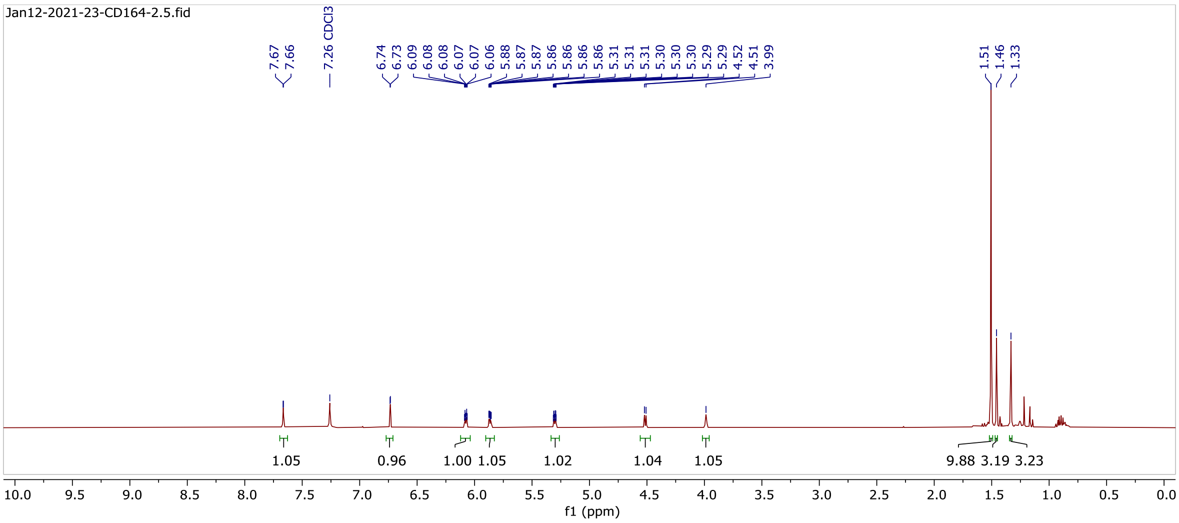

^{1}H NMR spectra of **3n**

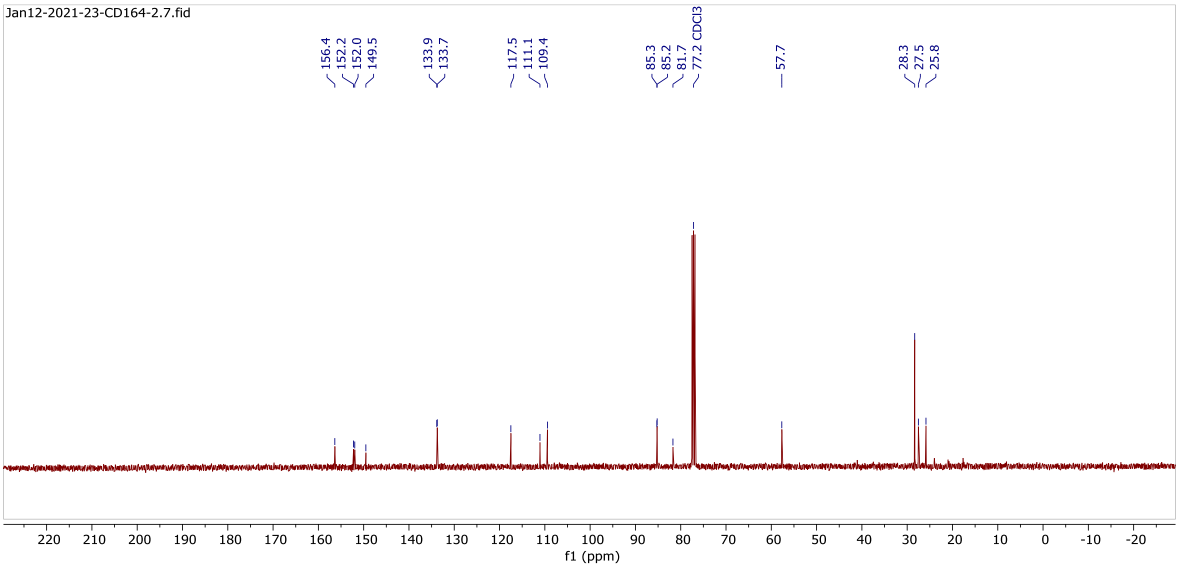

^{13}C NMR spectra of **3n**

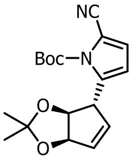

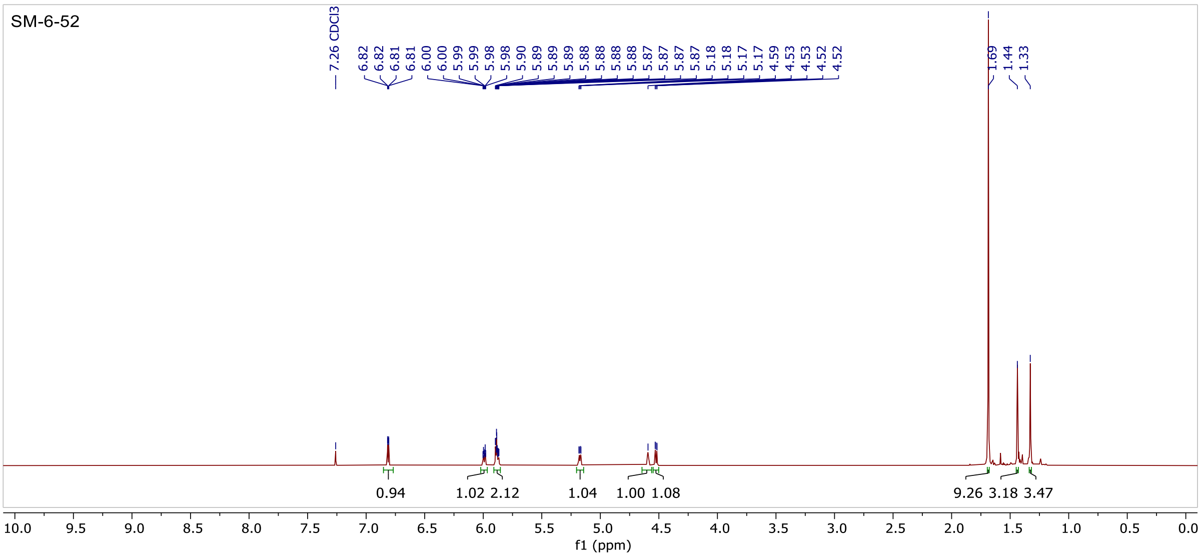

^{1}H NMR spectra of **3o**

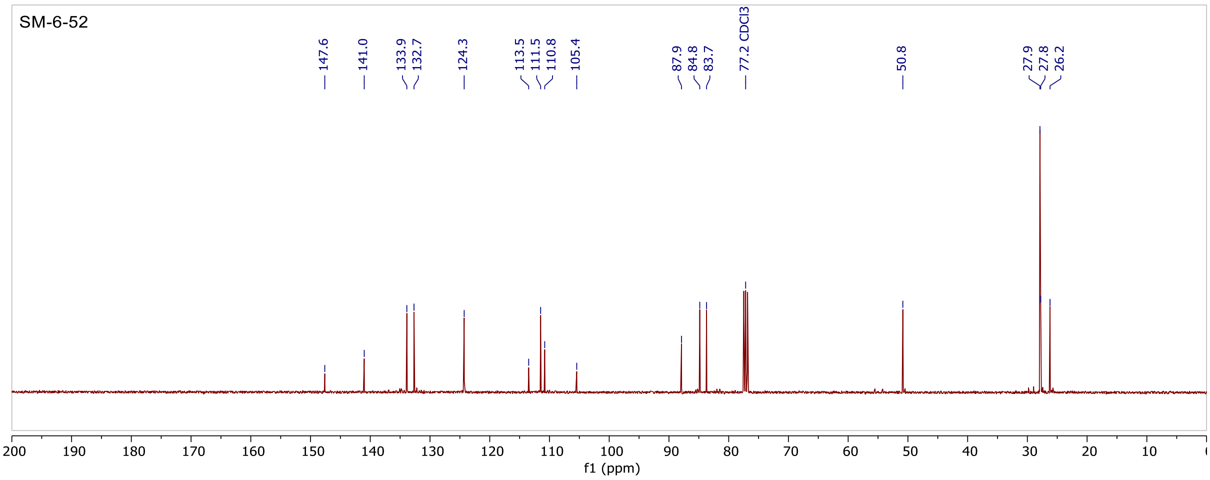

^{13}C NMR spectra of **3o**

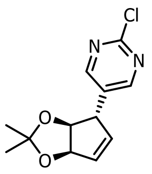

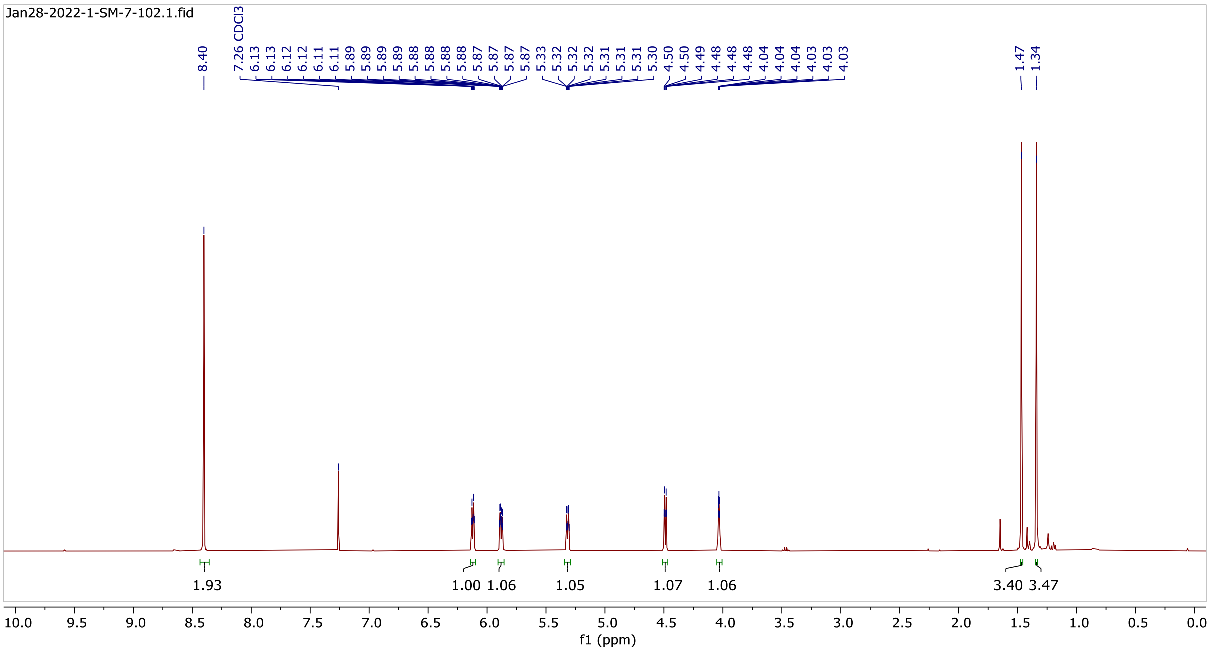

^{1}H NMR spectra of **3p**

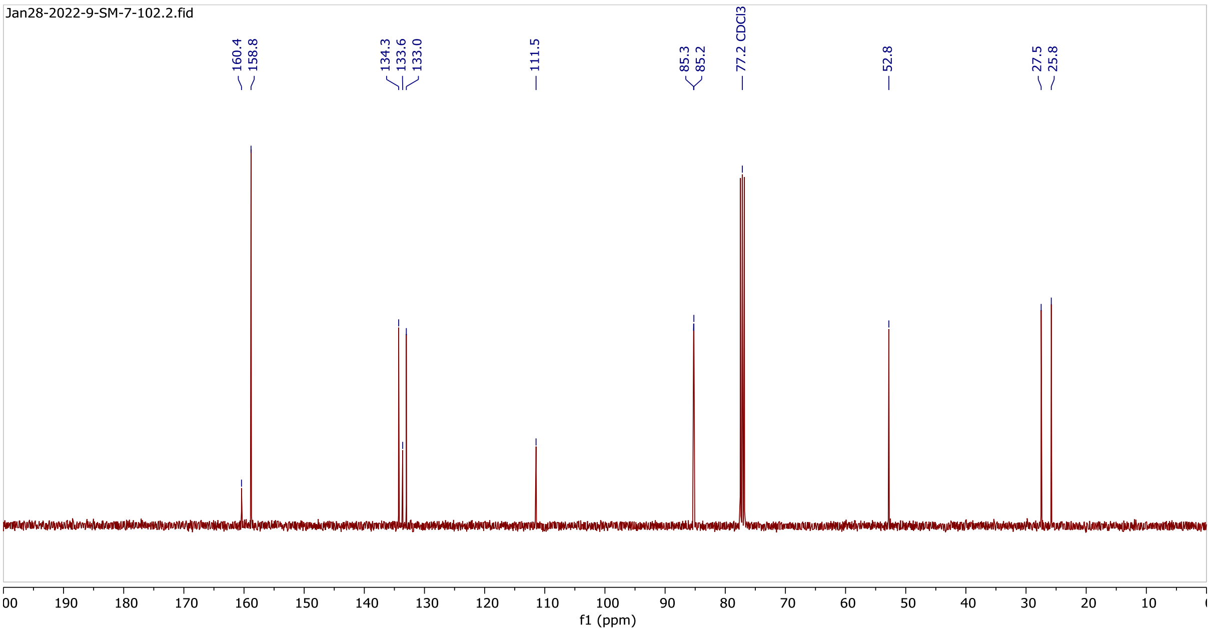

^{13}C NMR spectra of **3p**

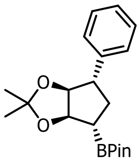

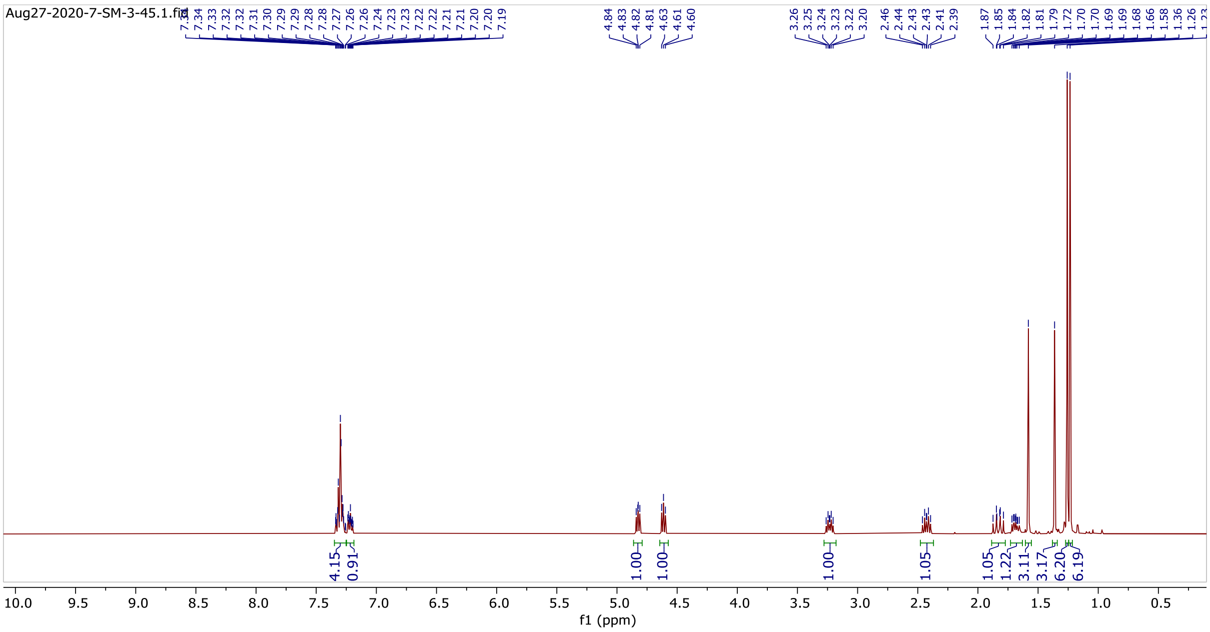

^{1}H NMR spectra of **4a**

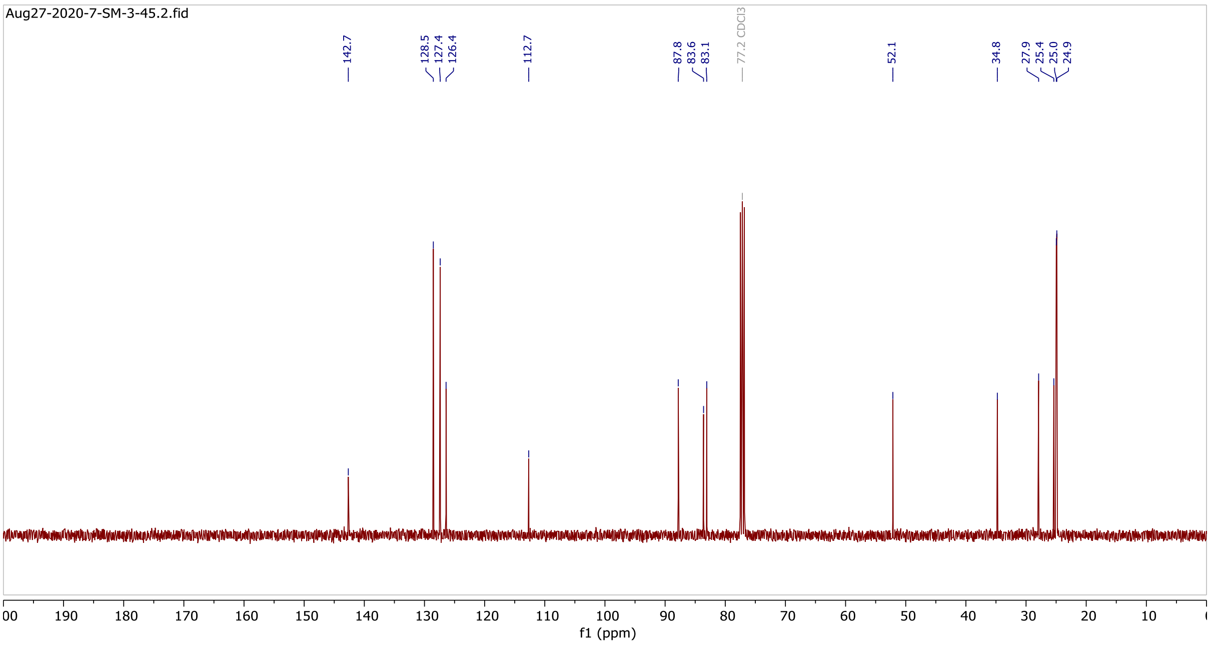

^{13}C NMR spectra of **4a**

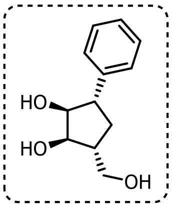

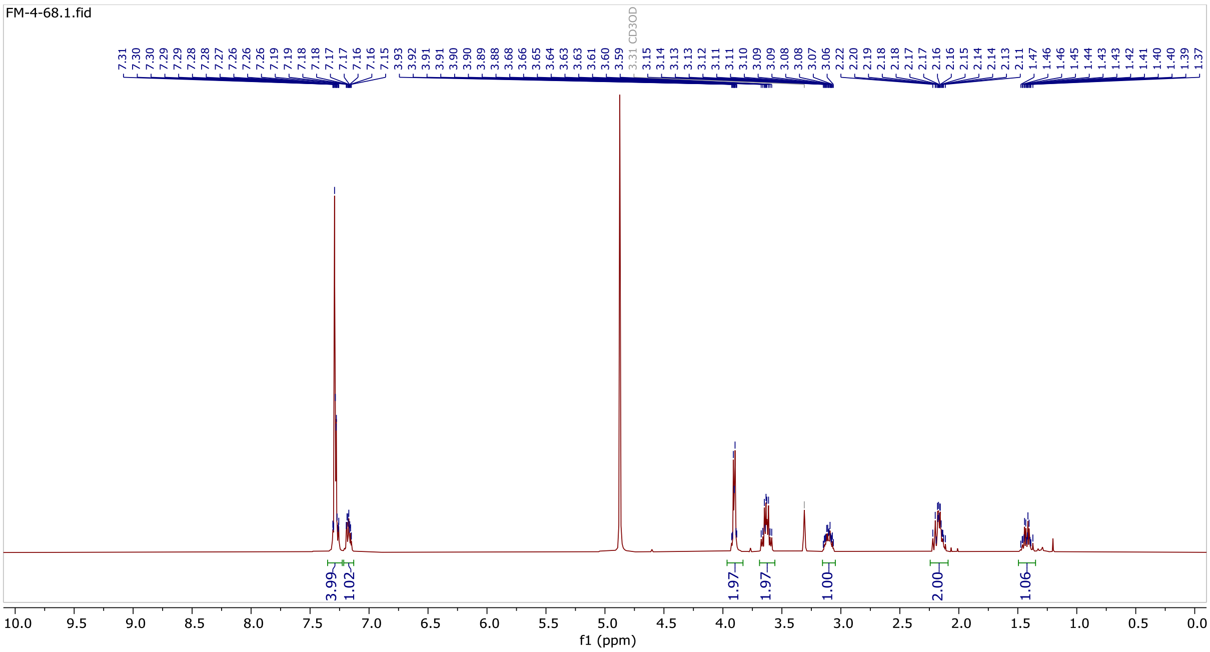

^{1}H NMR spectra of **7a**

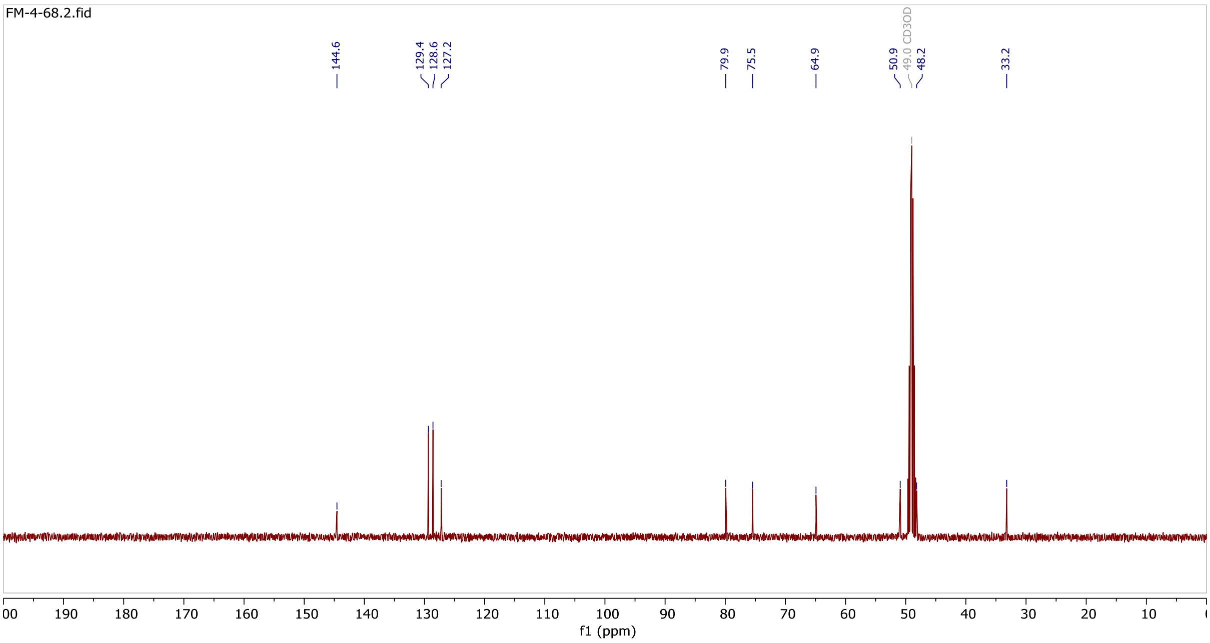

^{13}C NMR spectra of **7a**

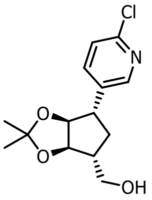

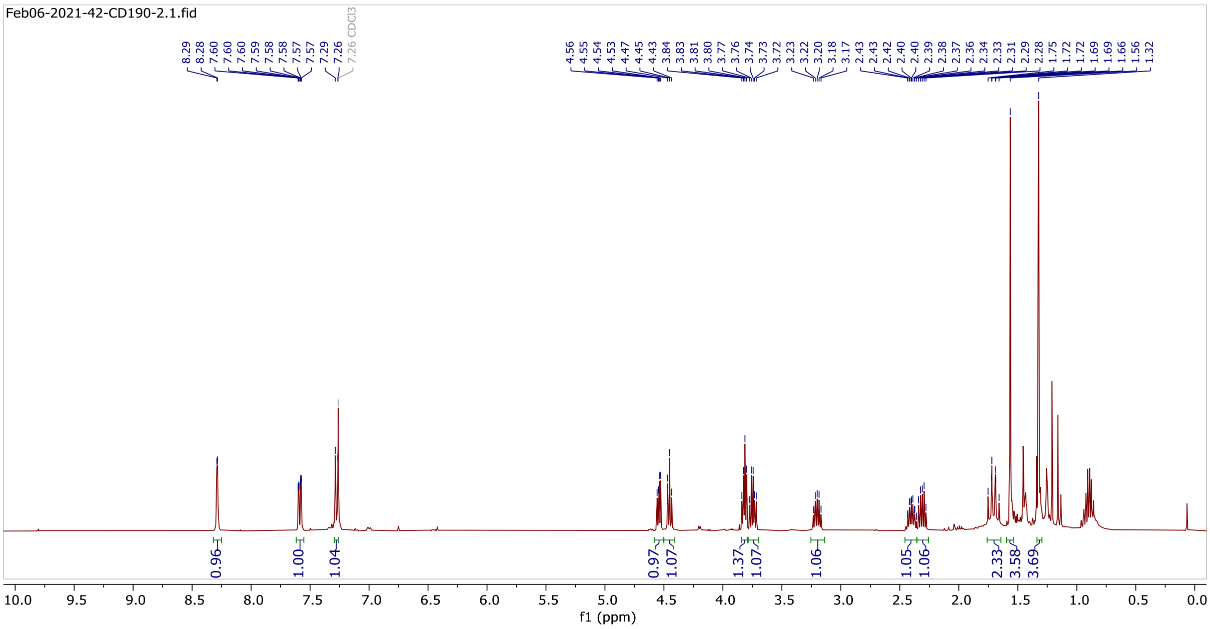

^{1}H NMR spectra of **6b**

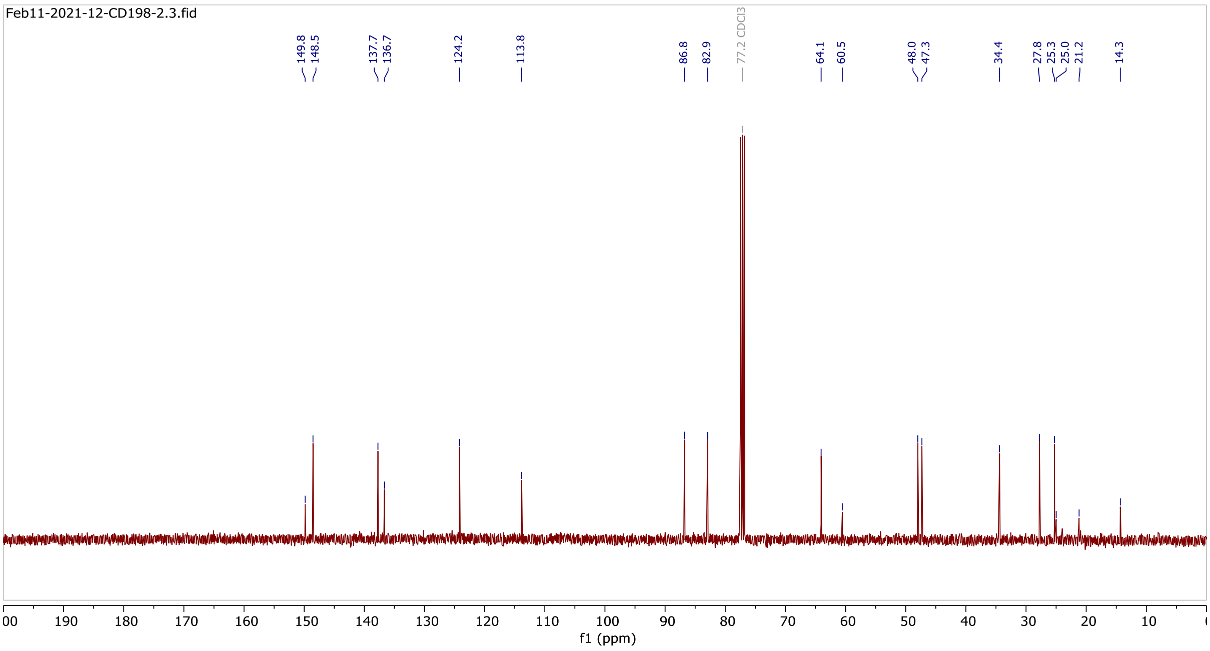

^{13}C NMR spectra of **6b**

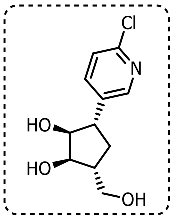

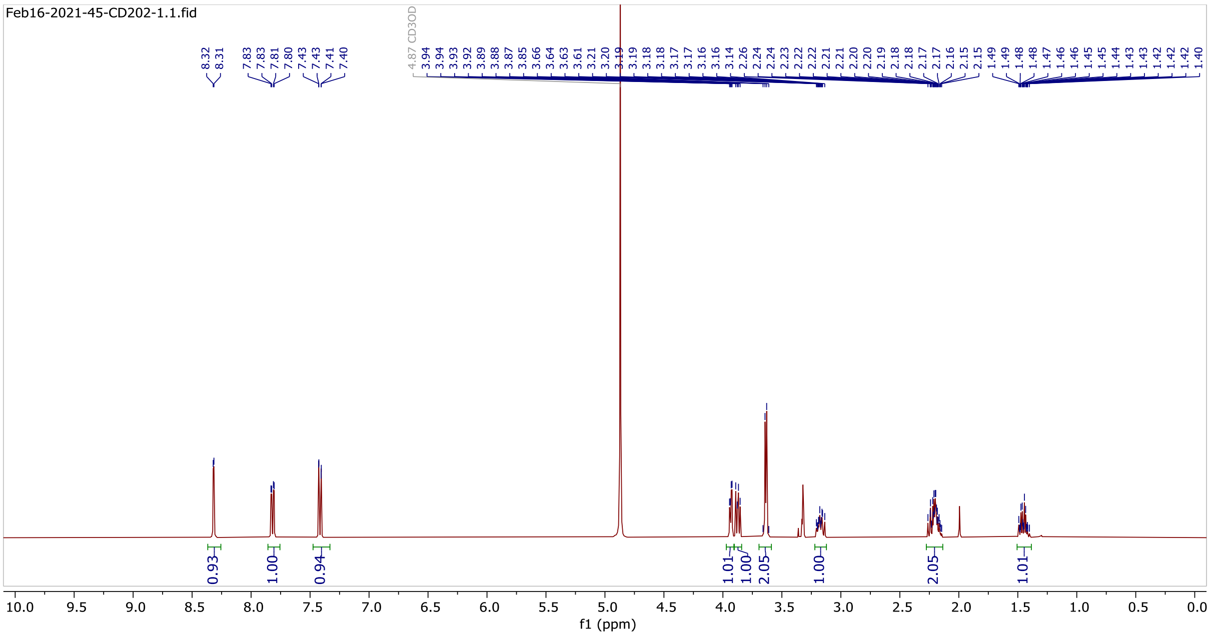

^{1}H NMR spectra of **7b**

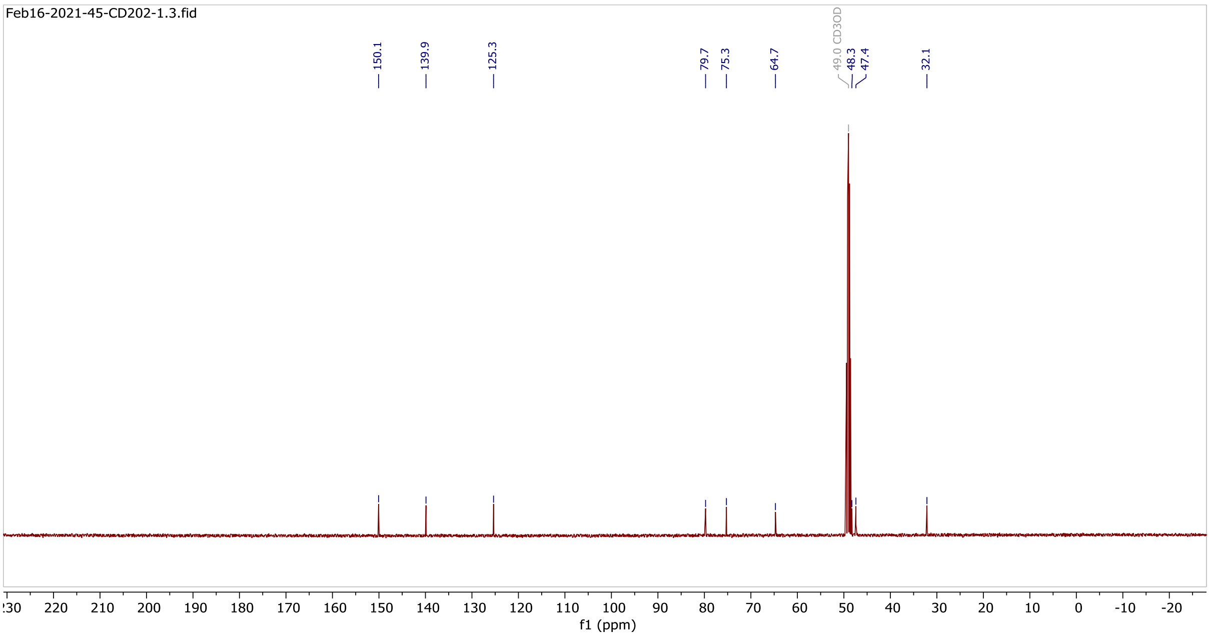

^{13}C NMR spectra of **7b**

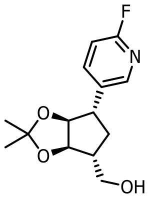

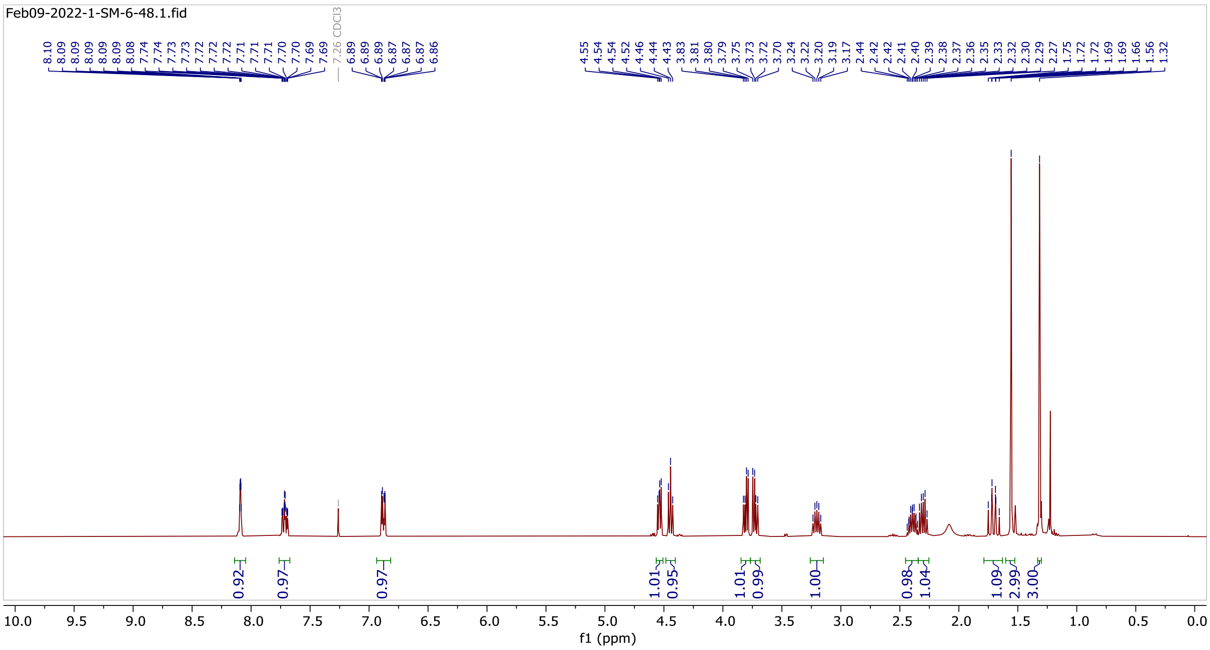

^{1}H NMR spectra of **6c**

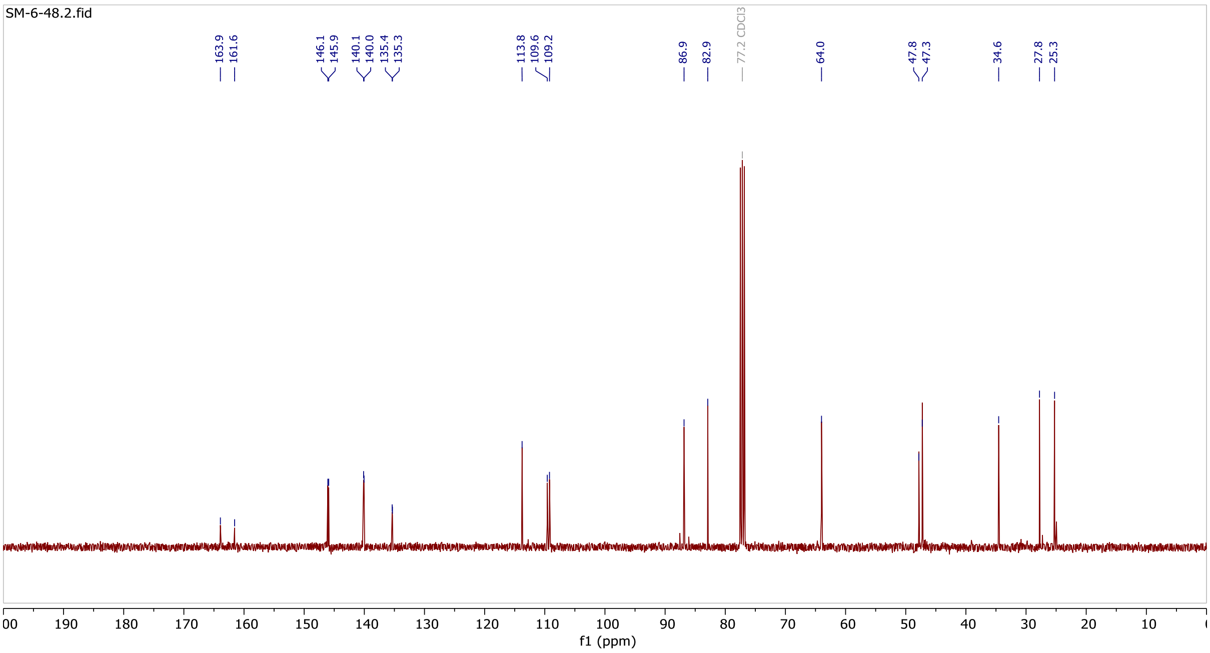

^{13}C NMR spectra of **6c**

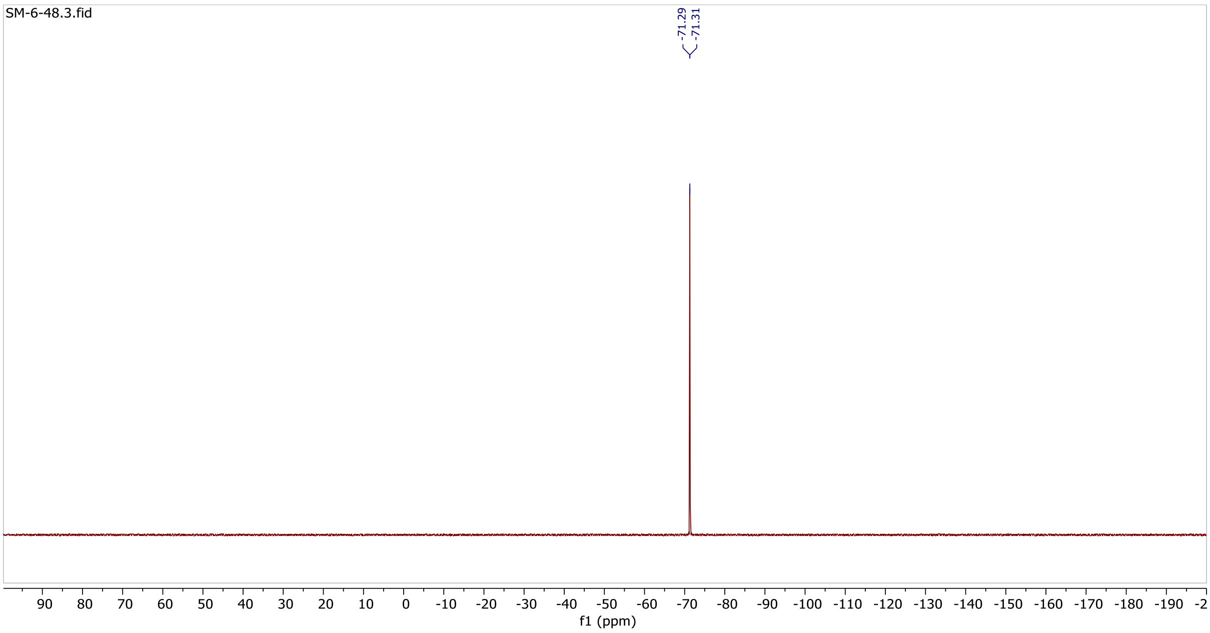

^{19}F NMR spectra of **6c**

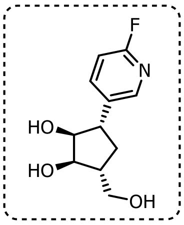

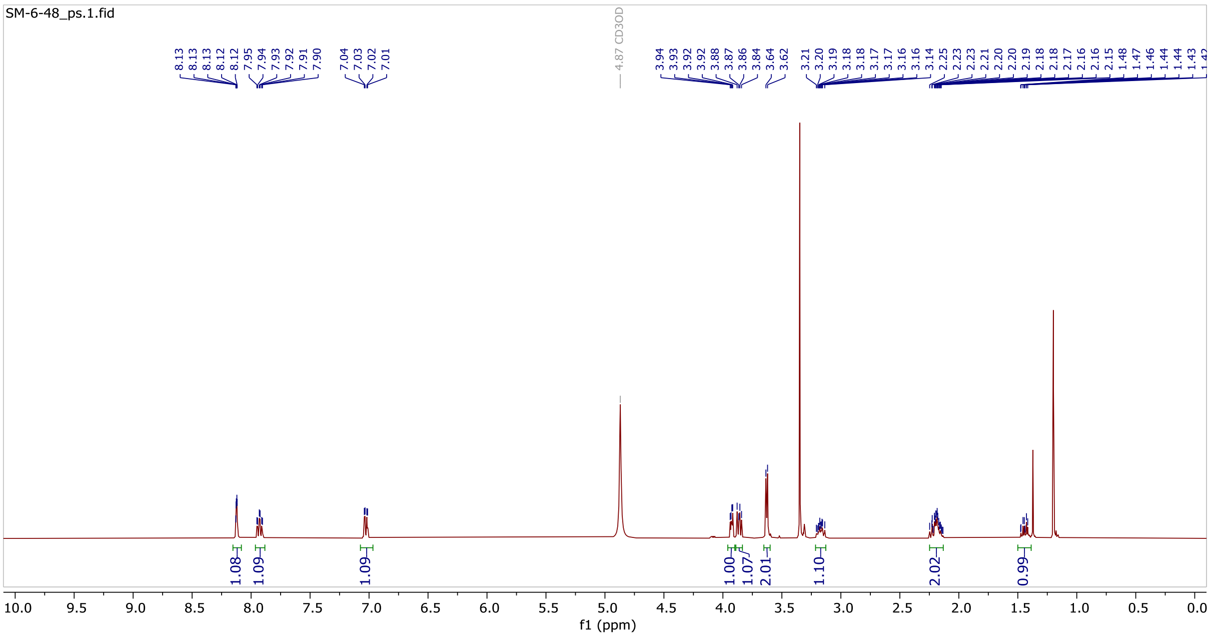

^{1}H NMR spectra of **7c**

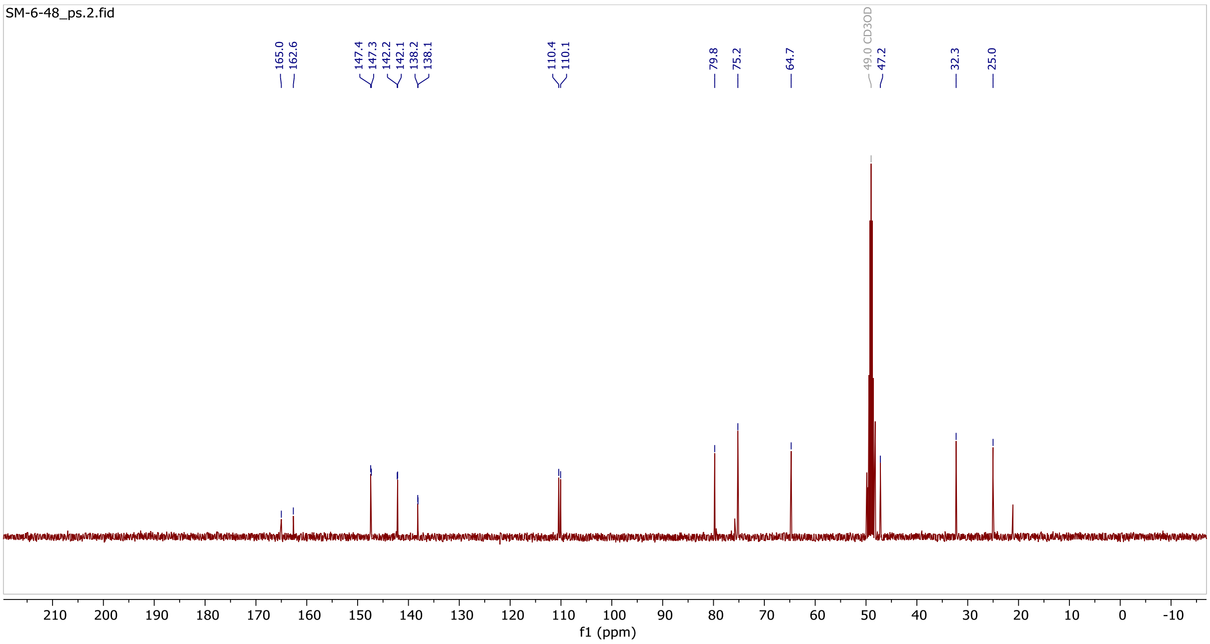

^{13}C NMR spectra of **7c**

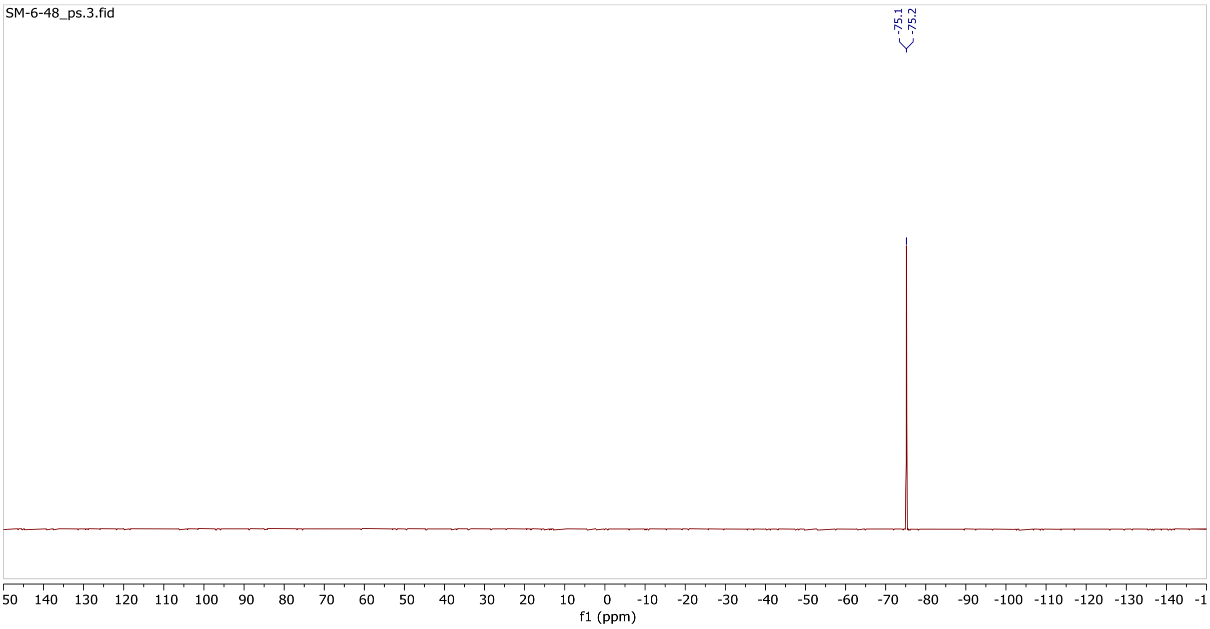

^{19}F NMR spectra of **7c**

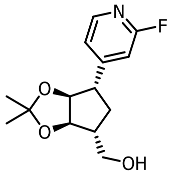

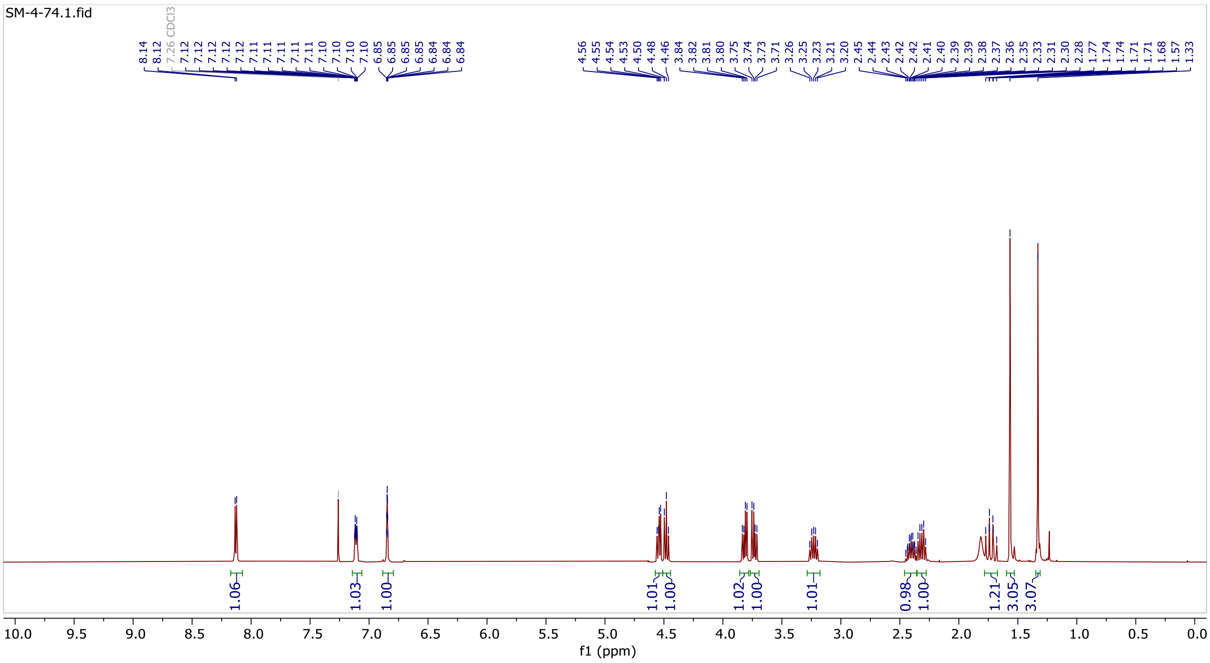

^{1}H NMR spectra of **6e**

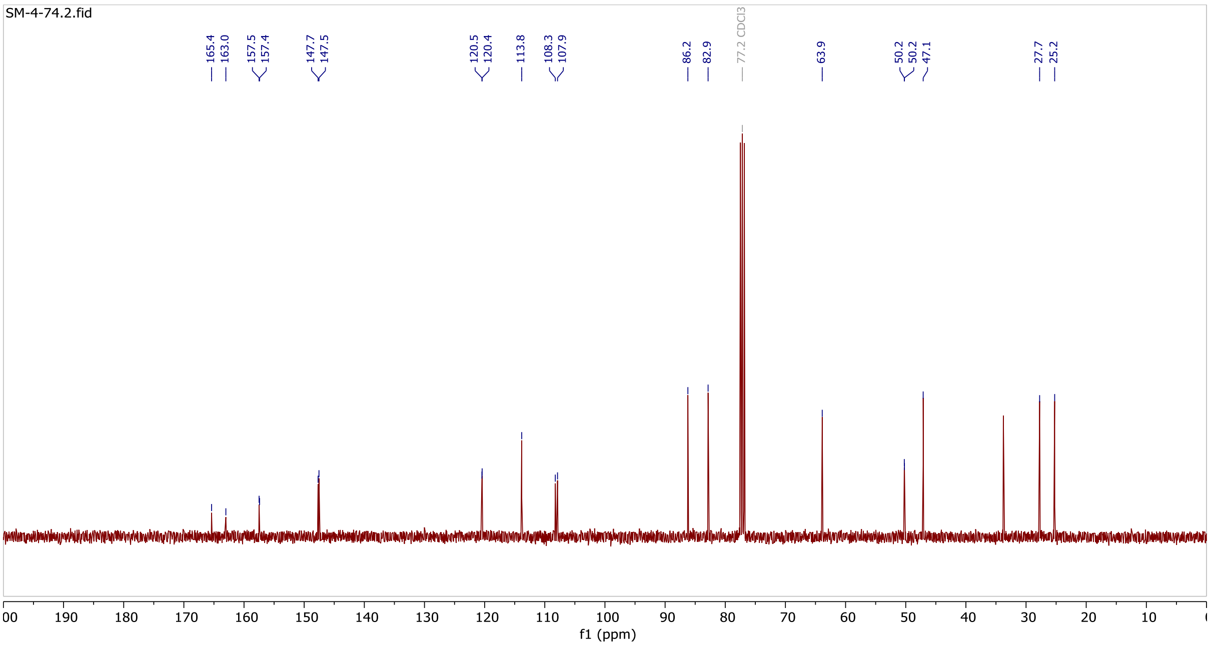

^{13}C NMR spectra of **6e**

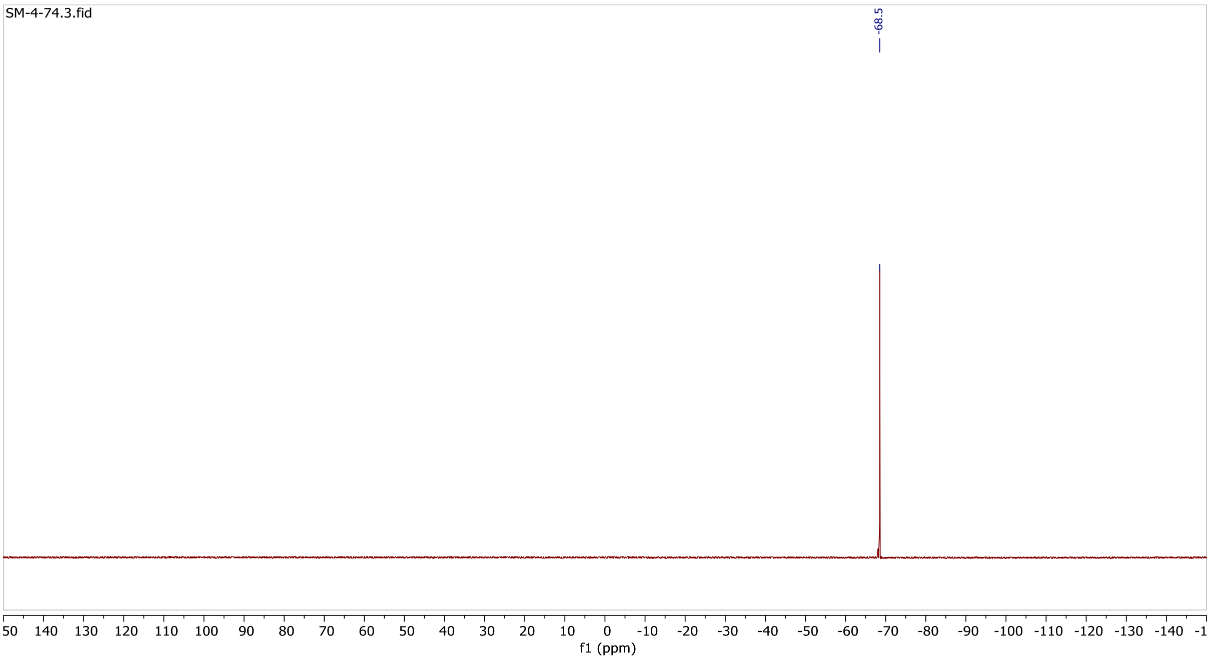

^{19}F NMR spectra of **6e**

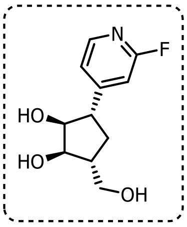

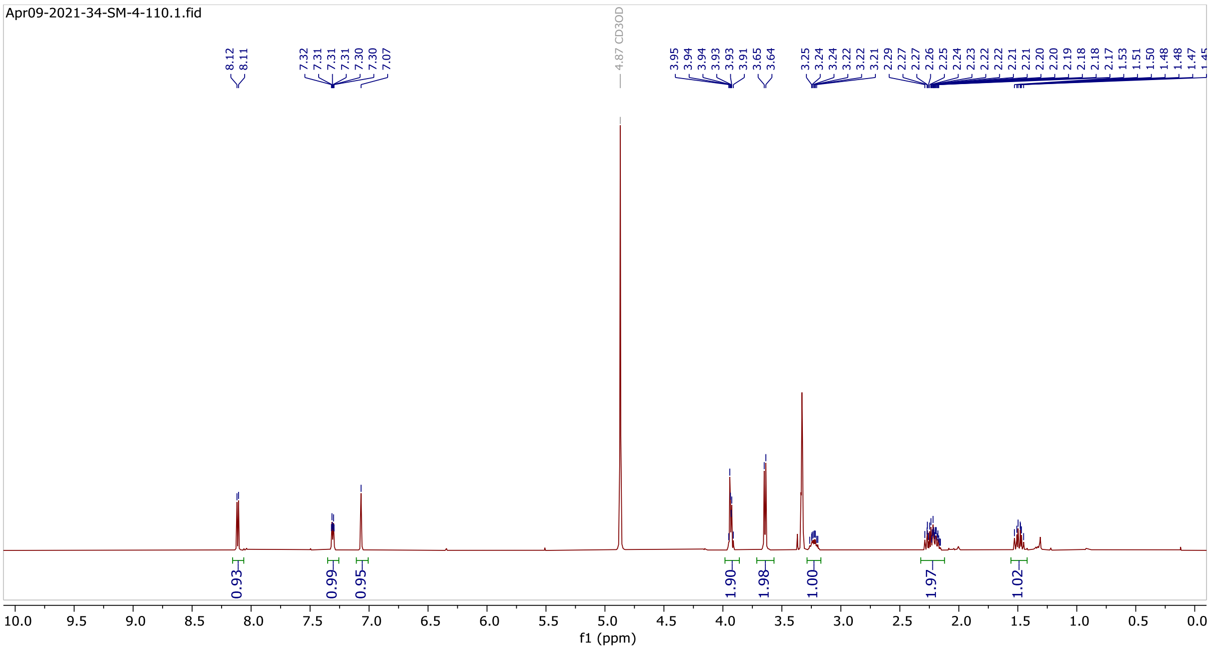

^{1}H NMR spectra of **7e**

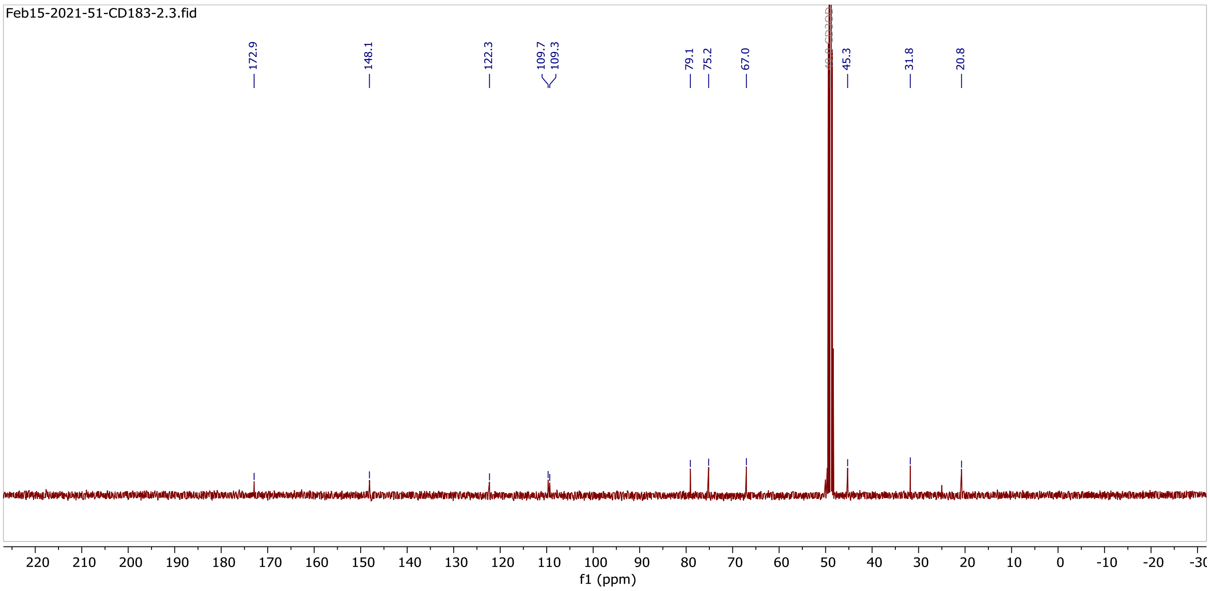

^{13}C NMR spectra of **7e**

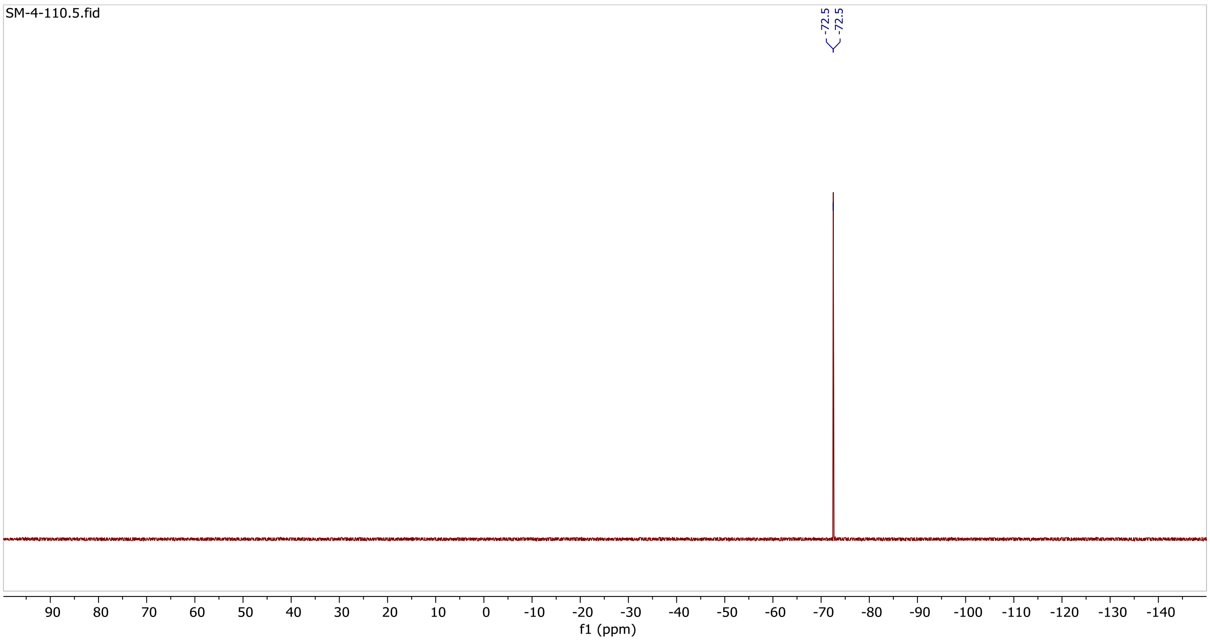

^{19}F NMR spectra of **7e**

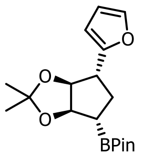

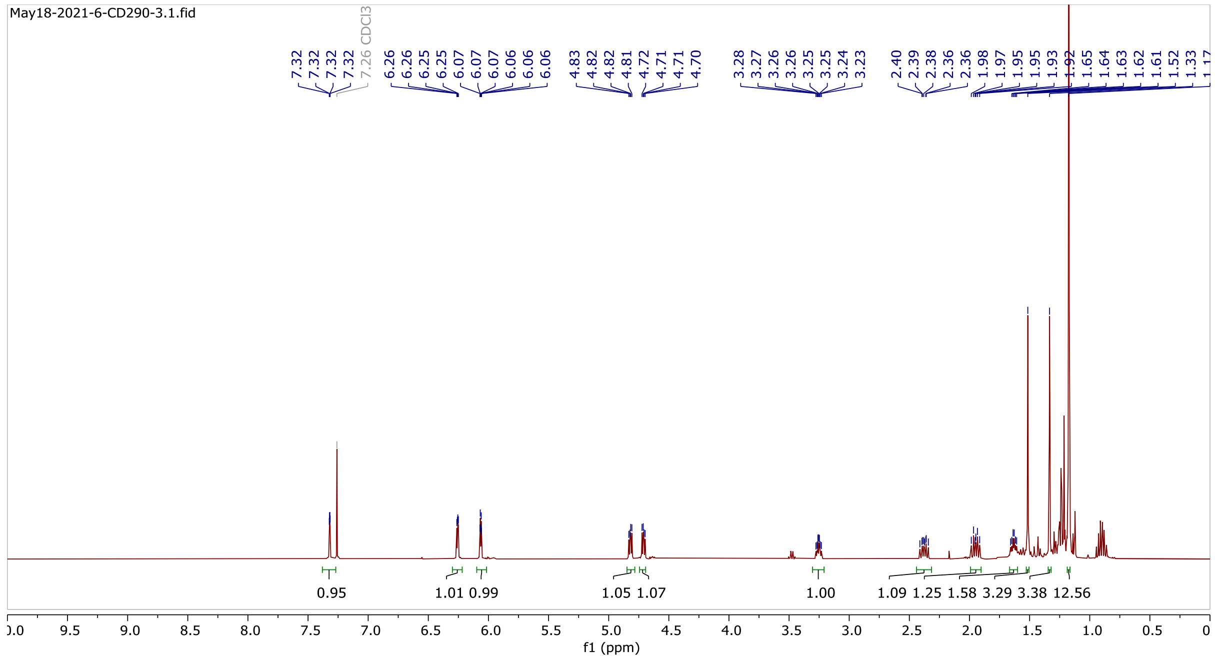

^{1}H NMR spectra of **4h**

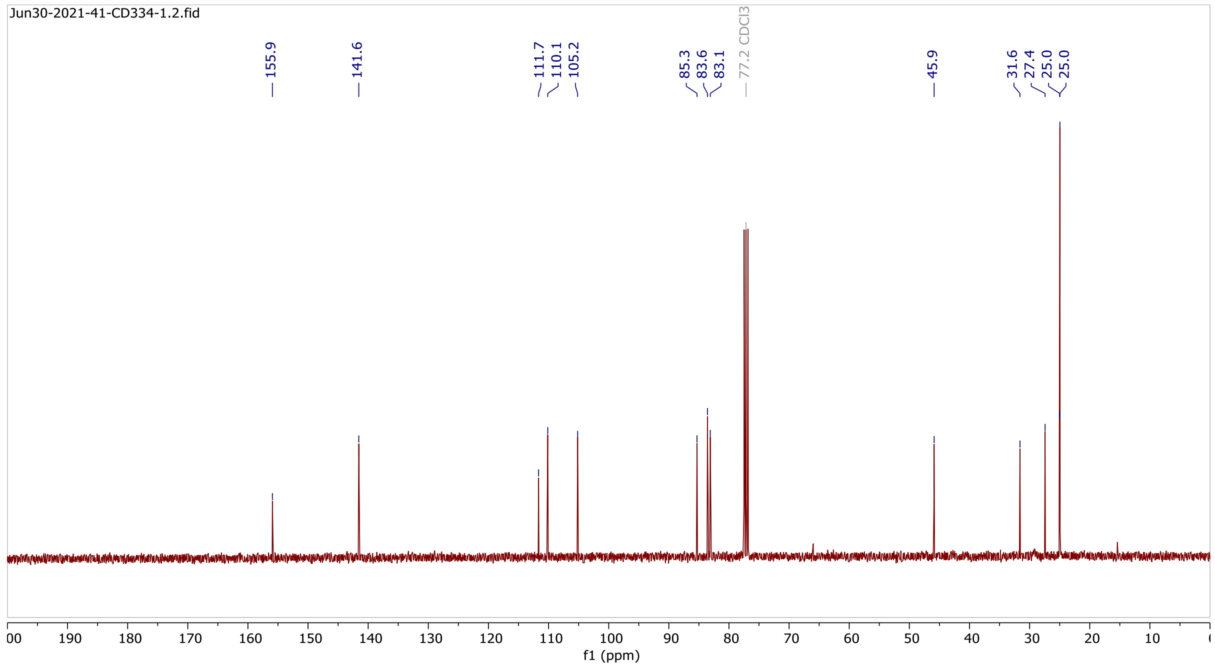

^{13}C NMR spectra of **4h**

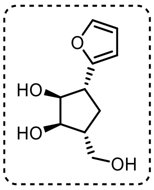

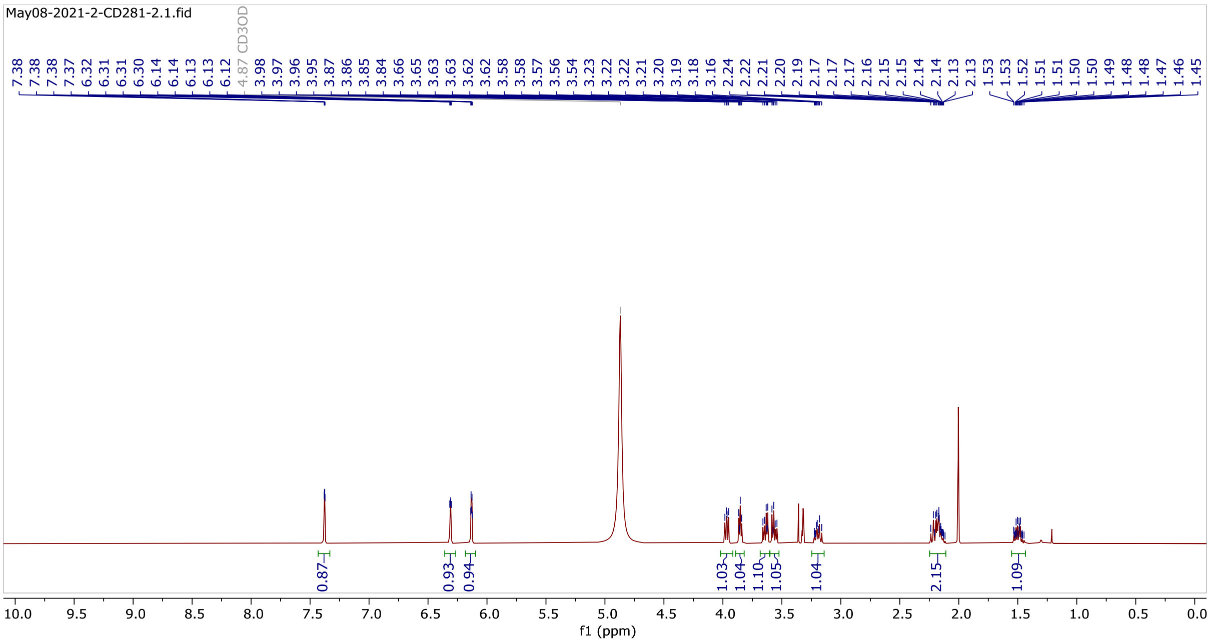

^{1}H NMR spectra of **7h**

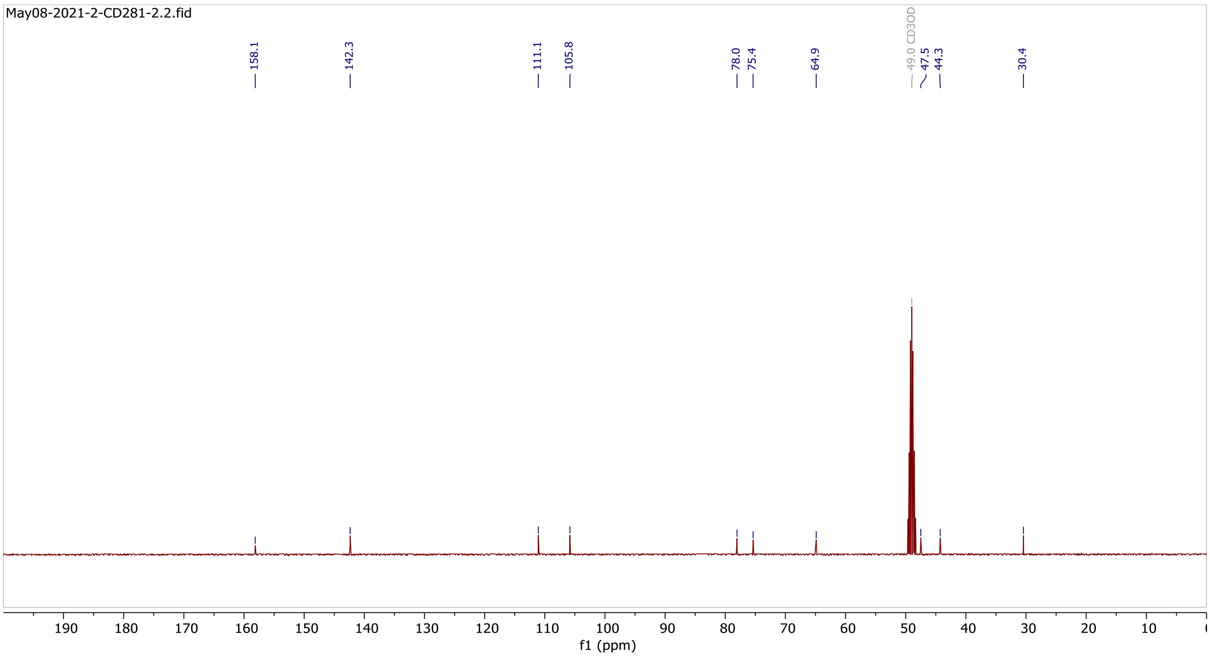

^{13}C NMR spectra of **7h**

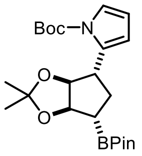

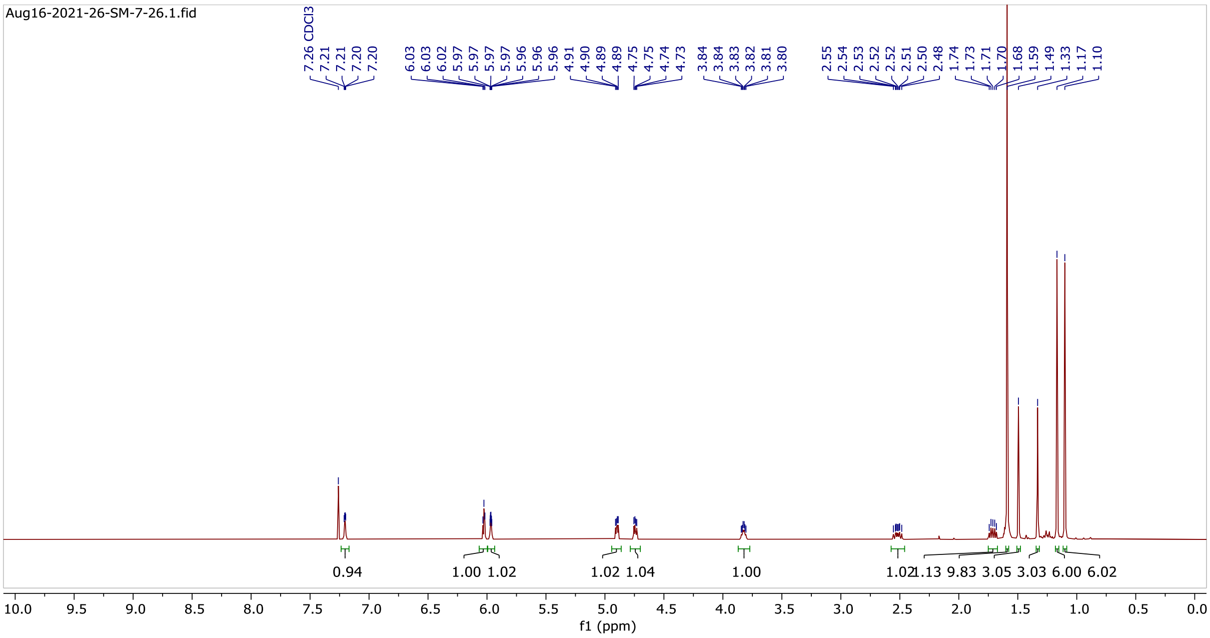

^{1}H NMR spectra of **4j**

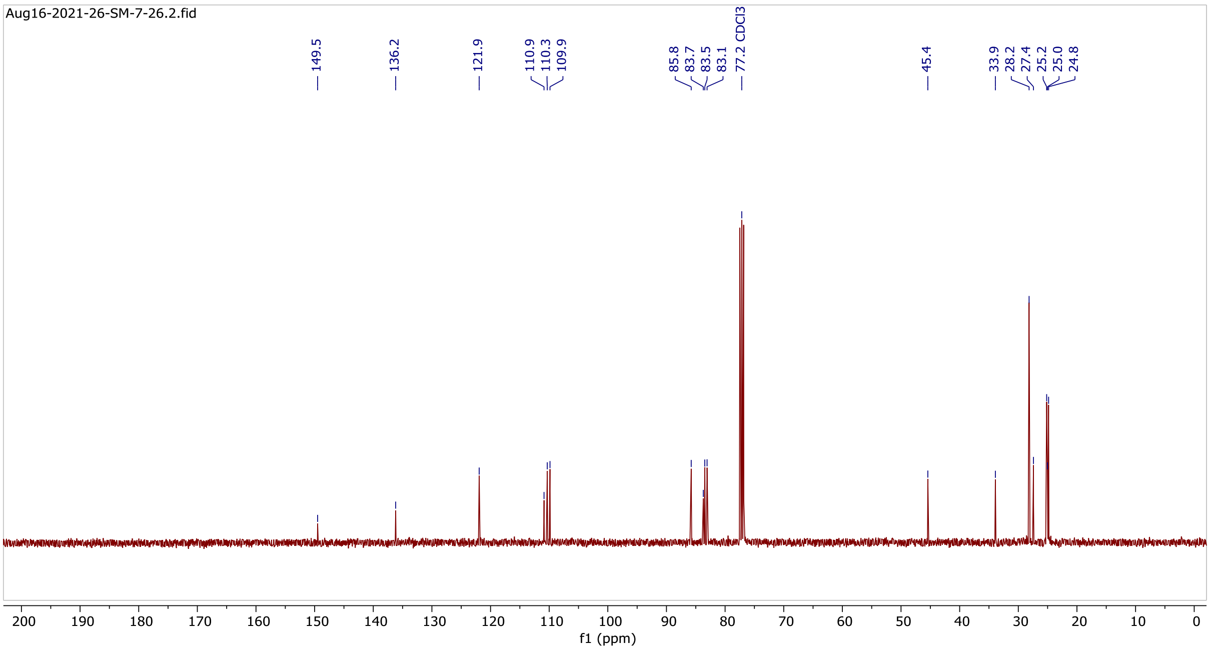

^{13}C NMR spectra of **4j**

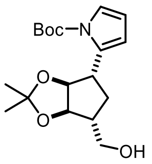

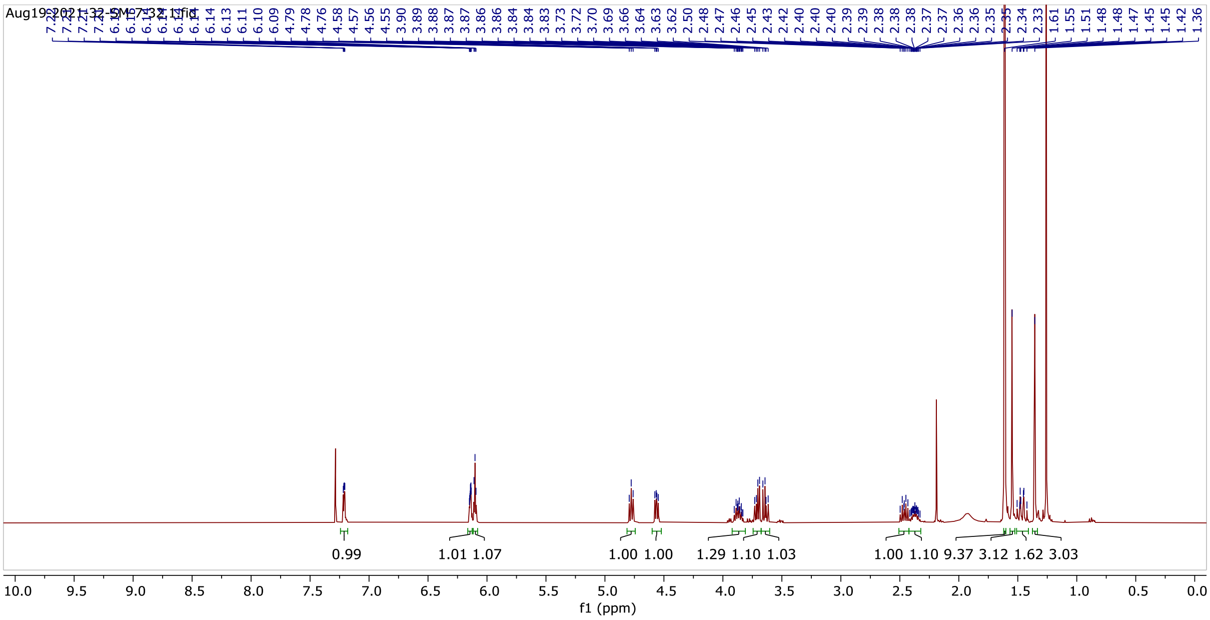

^{1}H NMR spectra of **6j**

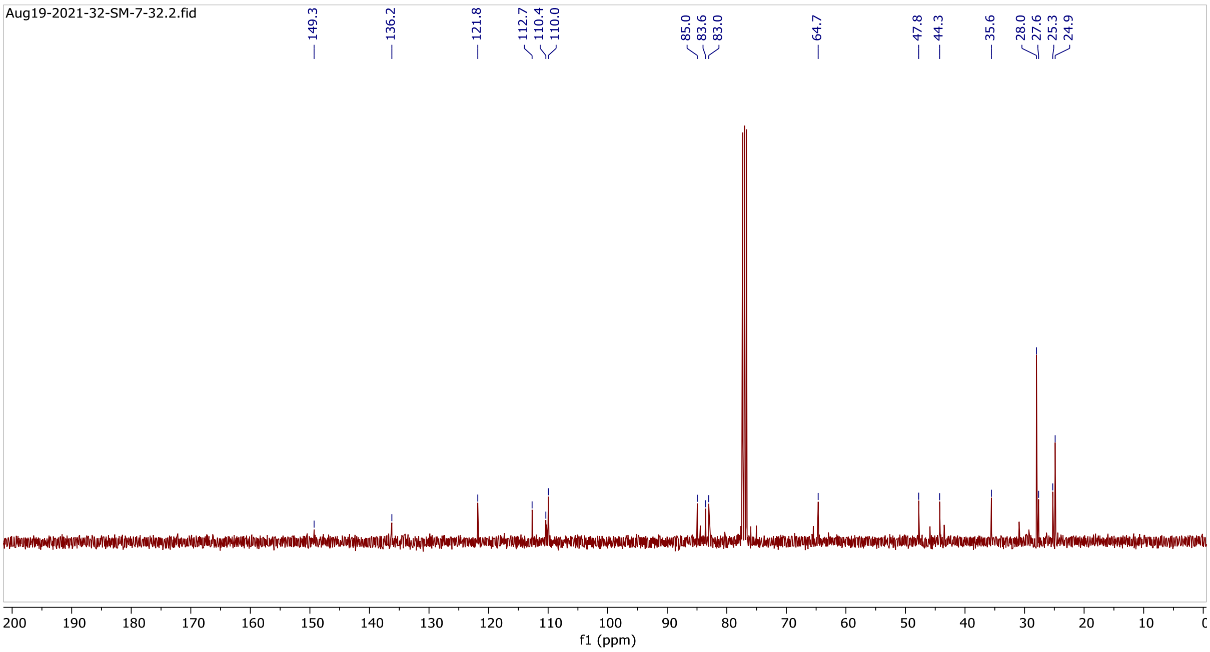

^{13}C NMR spectra of **6j**

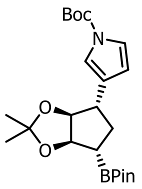

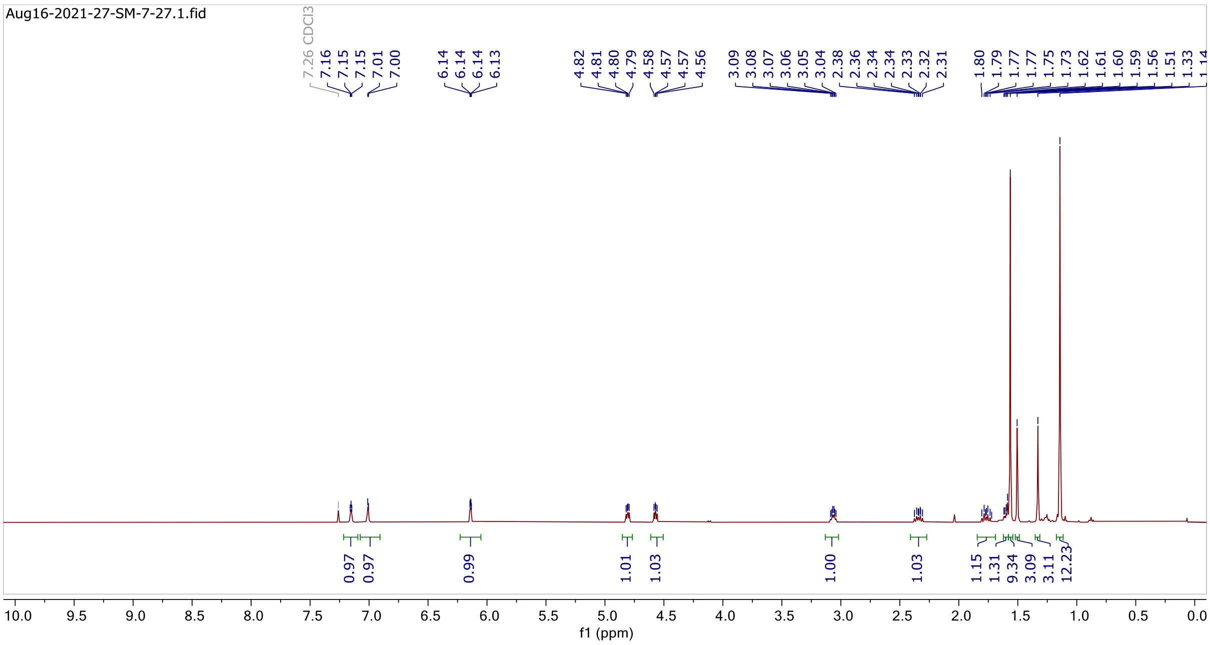

^{1}H NMR spectra of **4k**

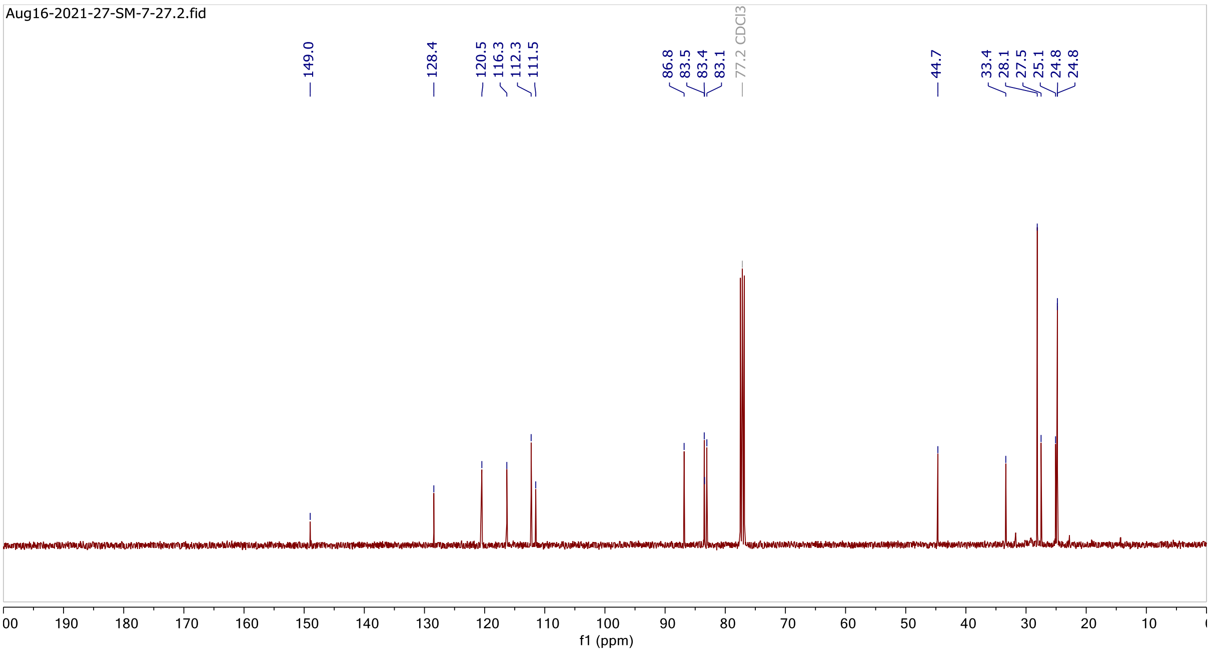

^{13}C NMR spectra of **4k**

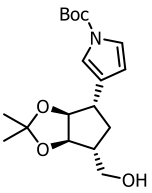

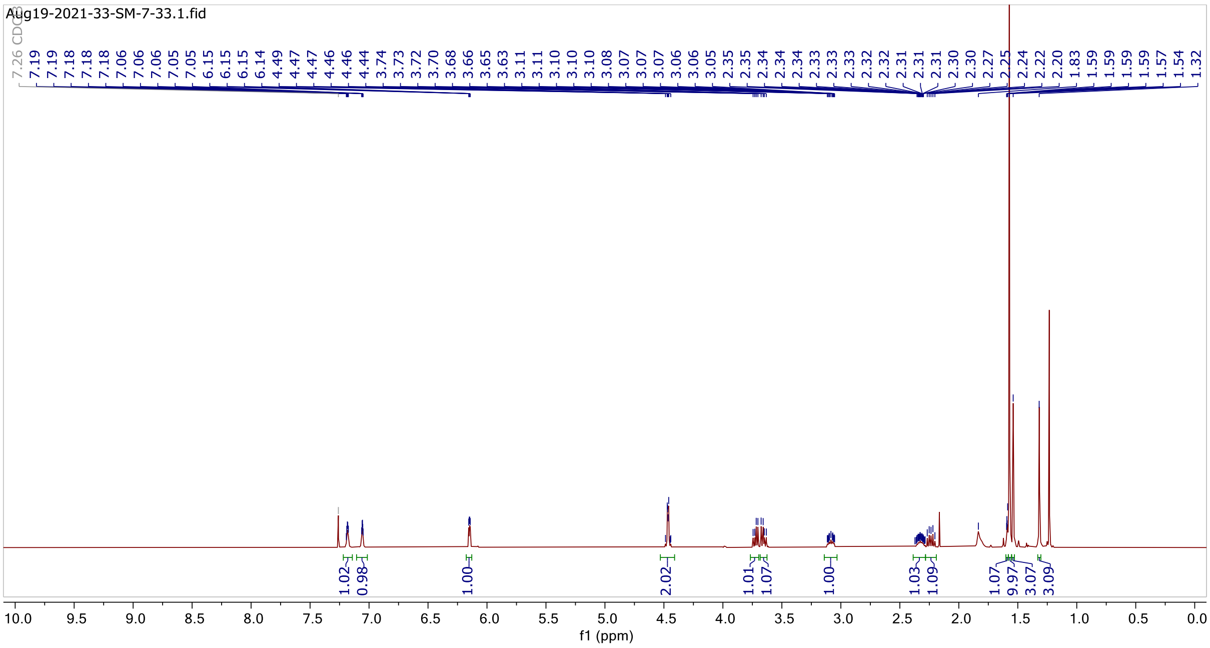

^{1}H NMR spectra of **6k**

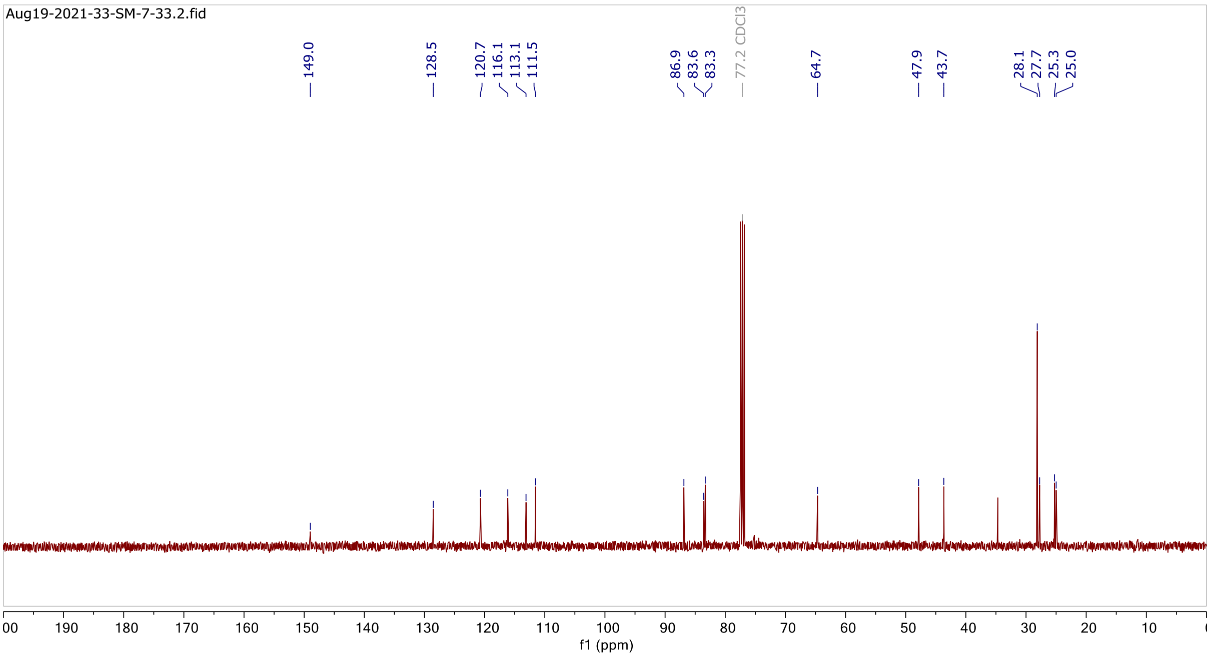

^{13}C NMR spectra of **6k**

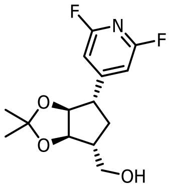

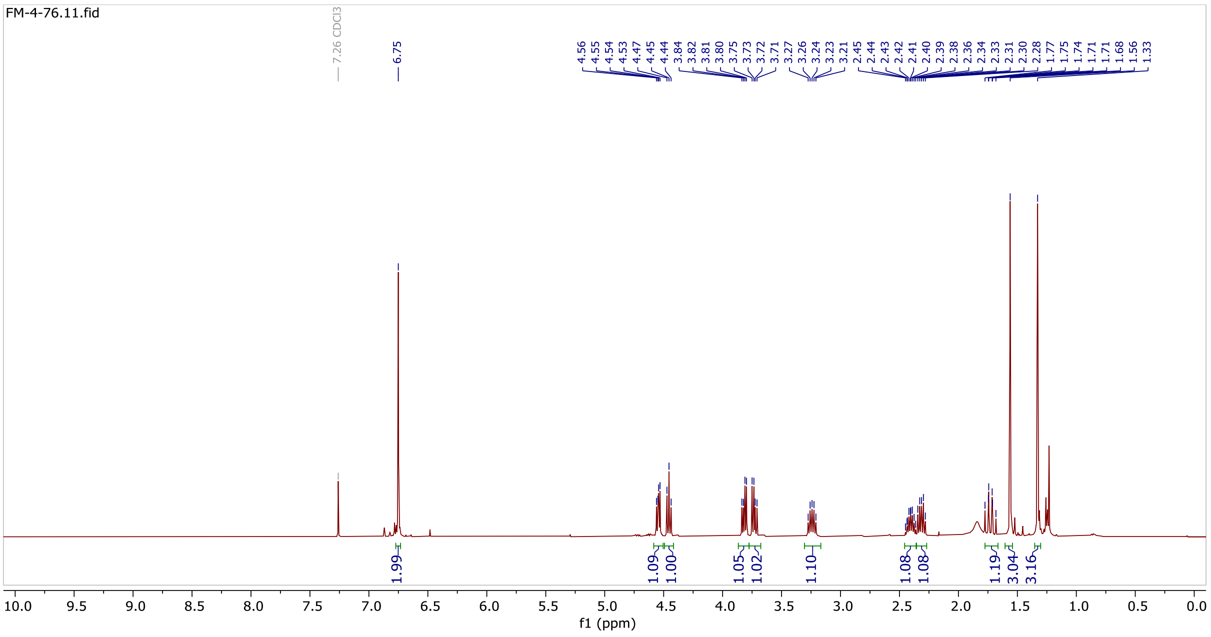

^{1}H NMR spectra of **6l**

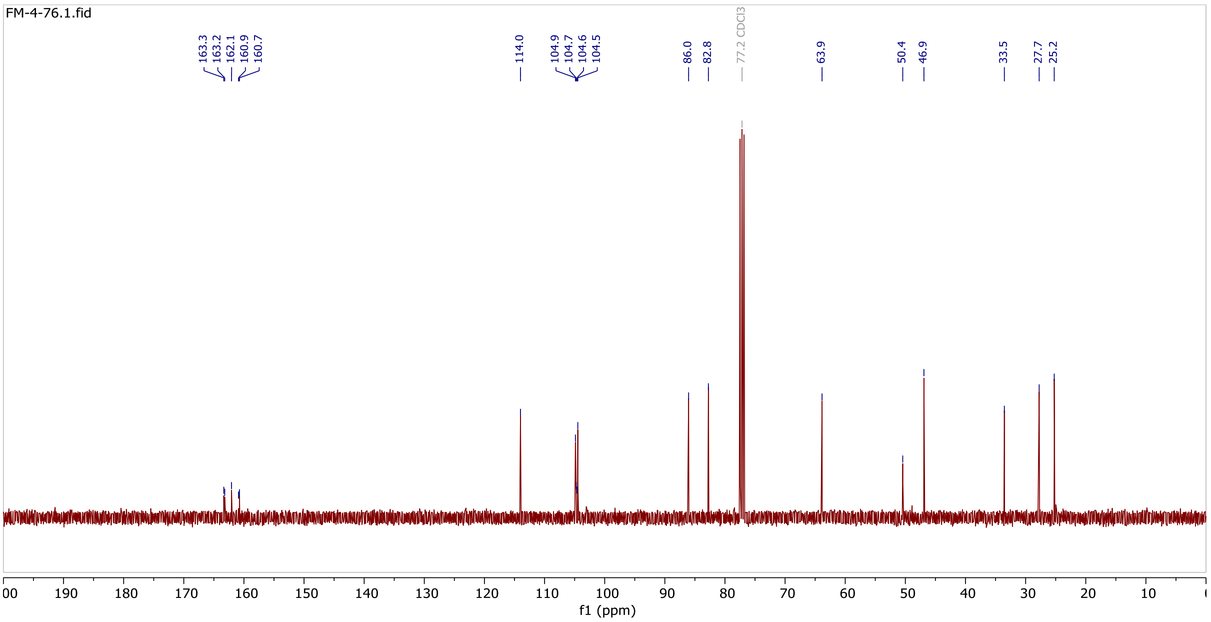

^{13}C NMR spectra of **6l**

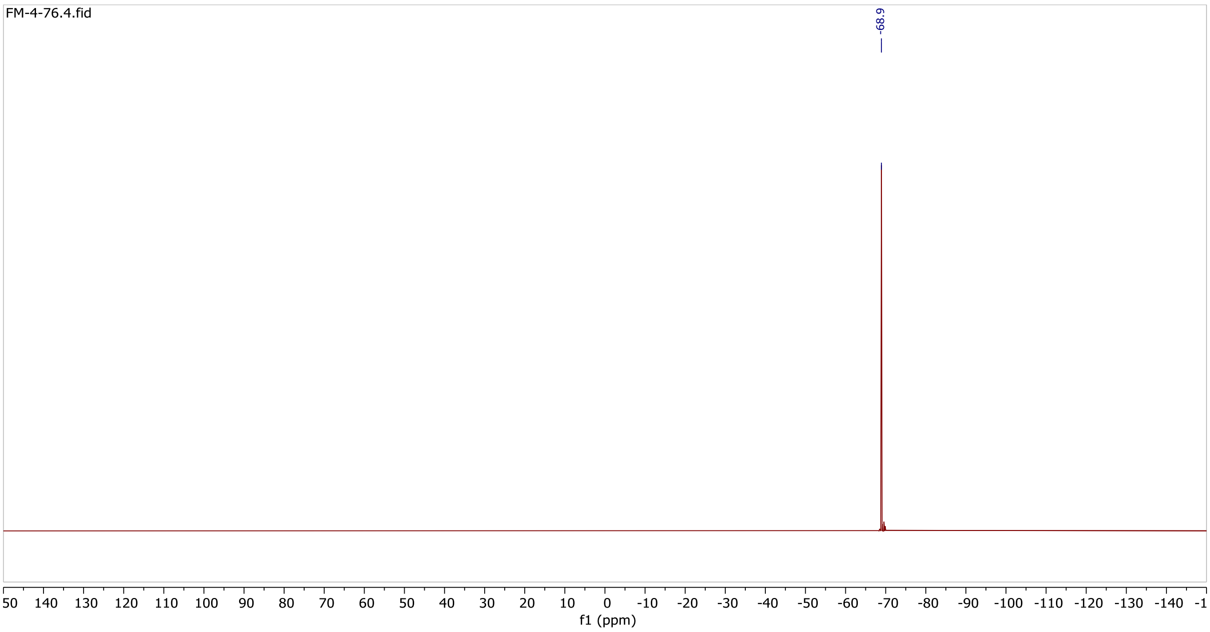

^{19}F NMR spectra of **6l**

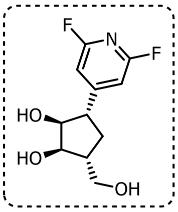

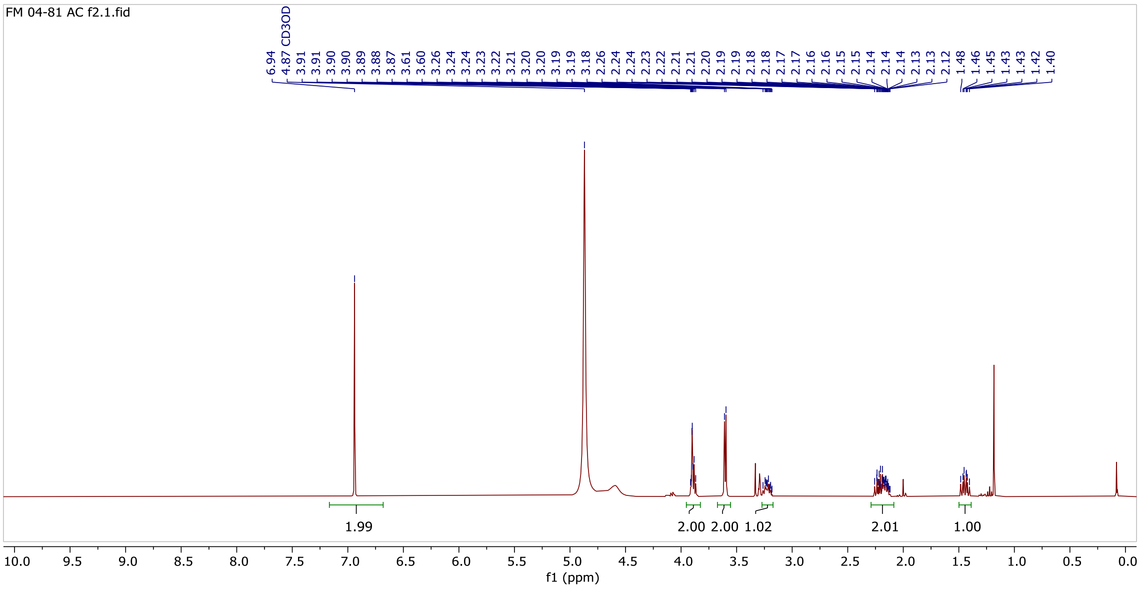

^1H NMR spectra of **7l**

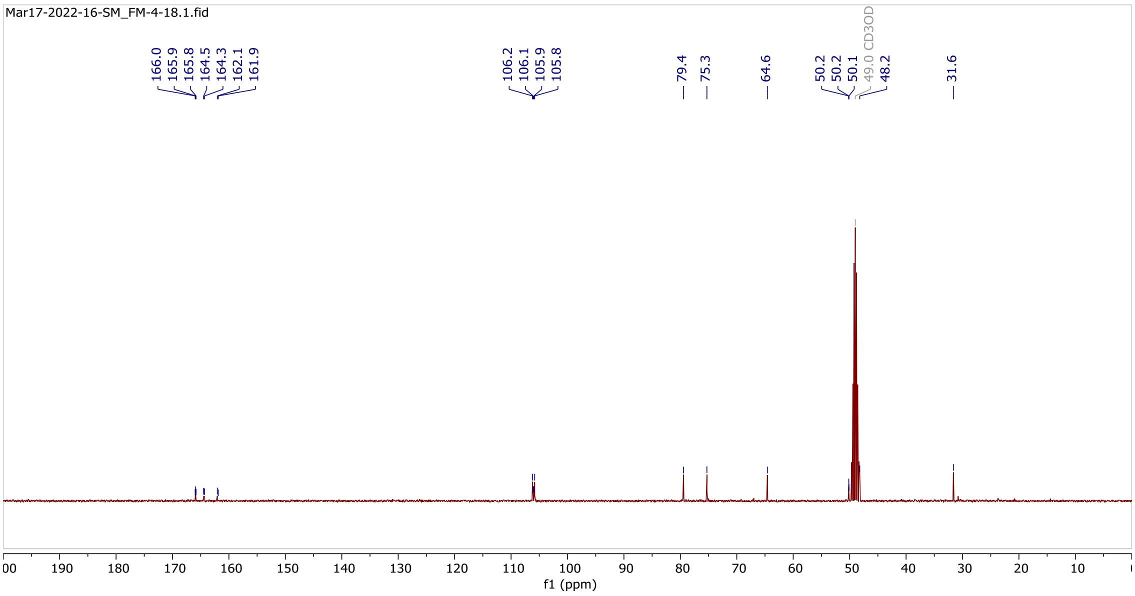

^{13}C NMR spectra of **7l**

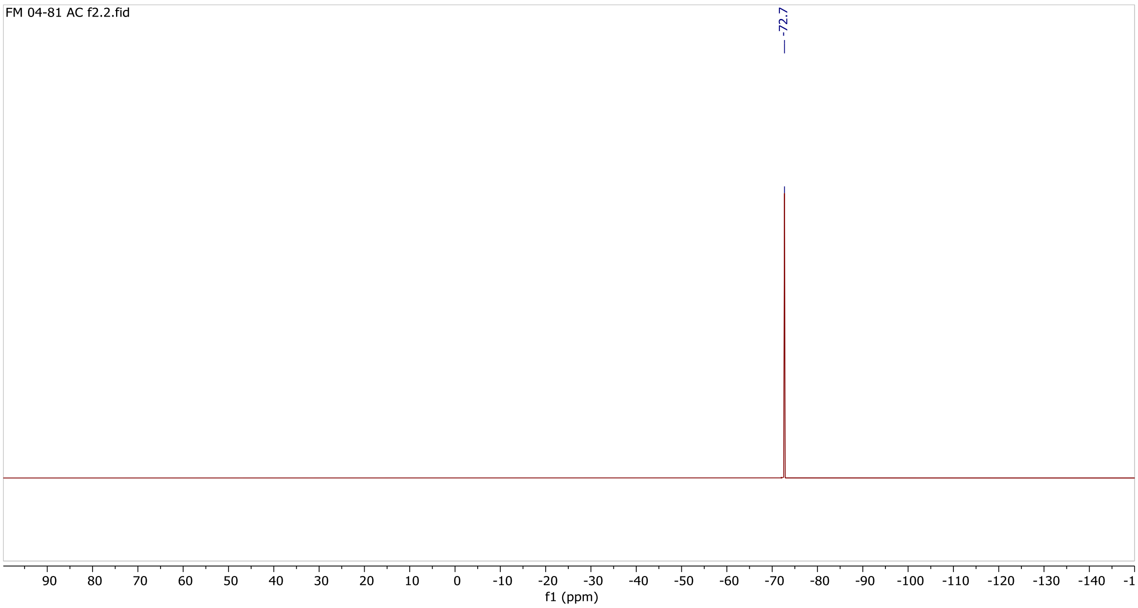

^{19}F NMR spectra of **7l**

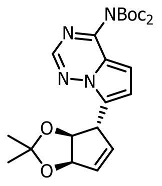

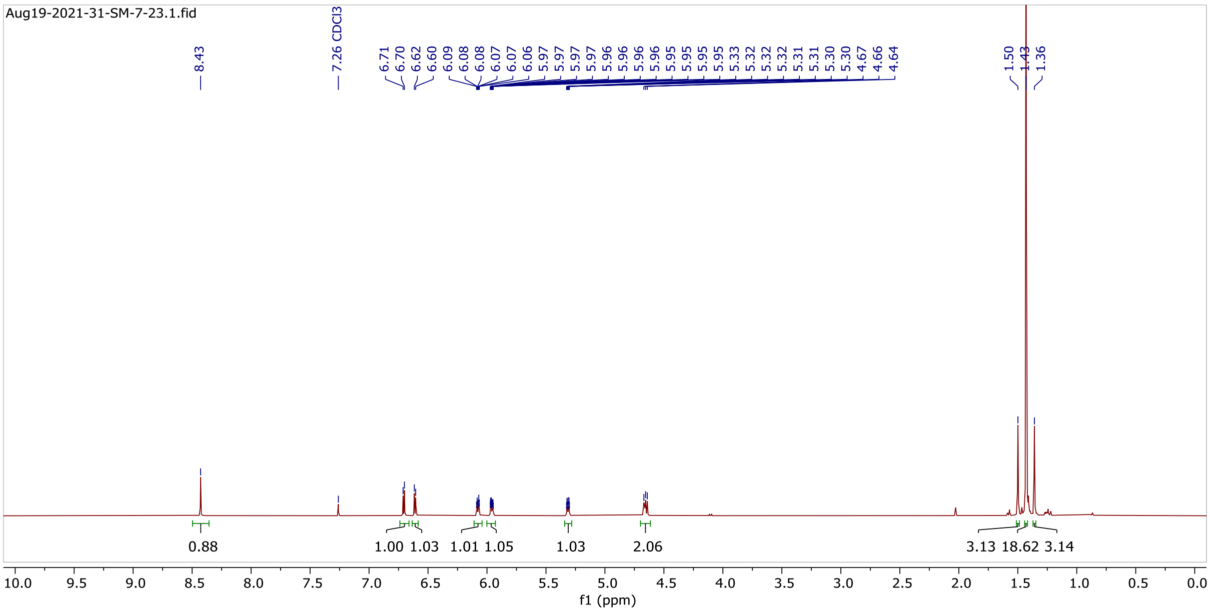

^{1}H NMR spectra of **3q**

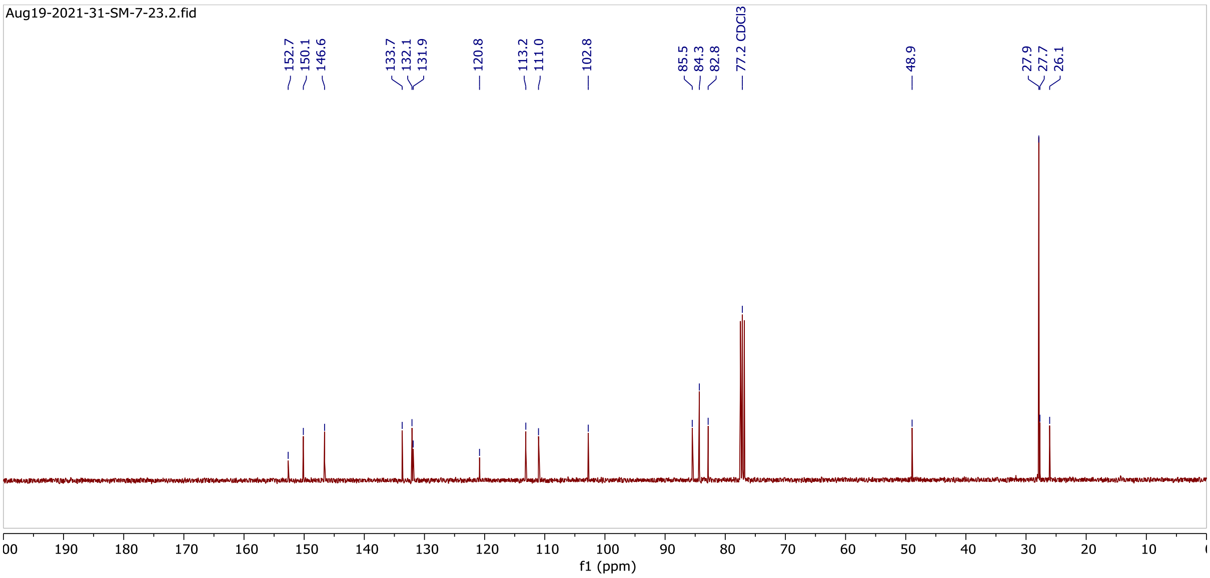

^{13}C NMR spectra of **3q**

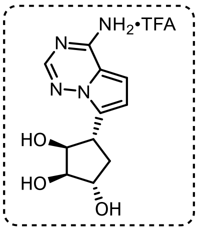

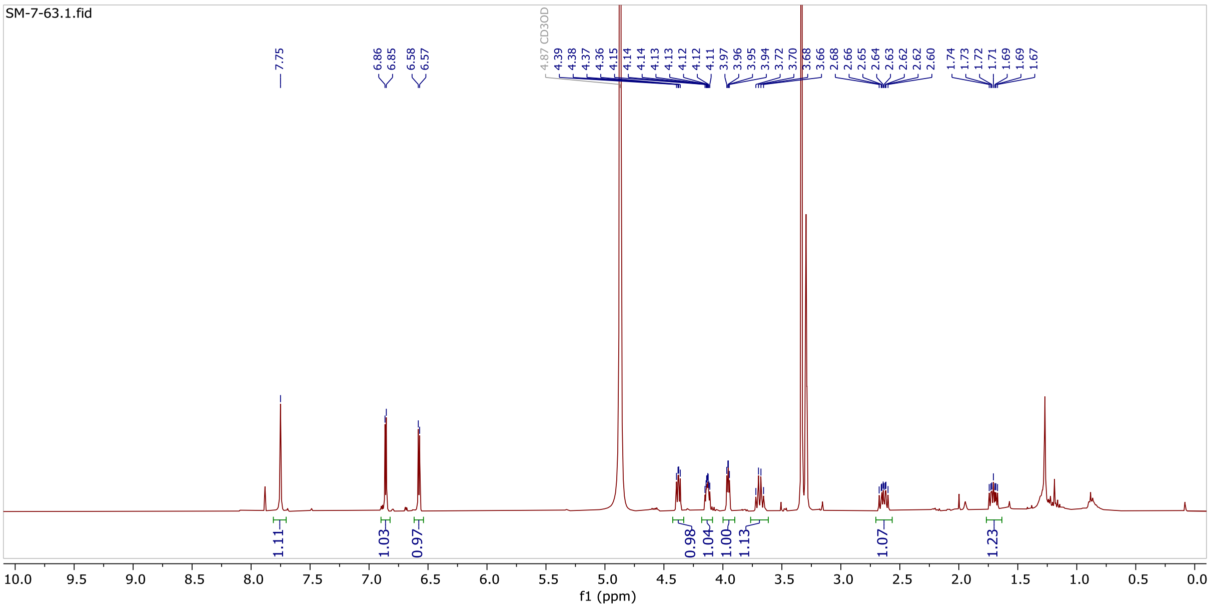

^{1}H NMR spectra of **10**

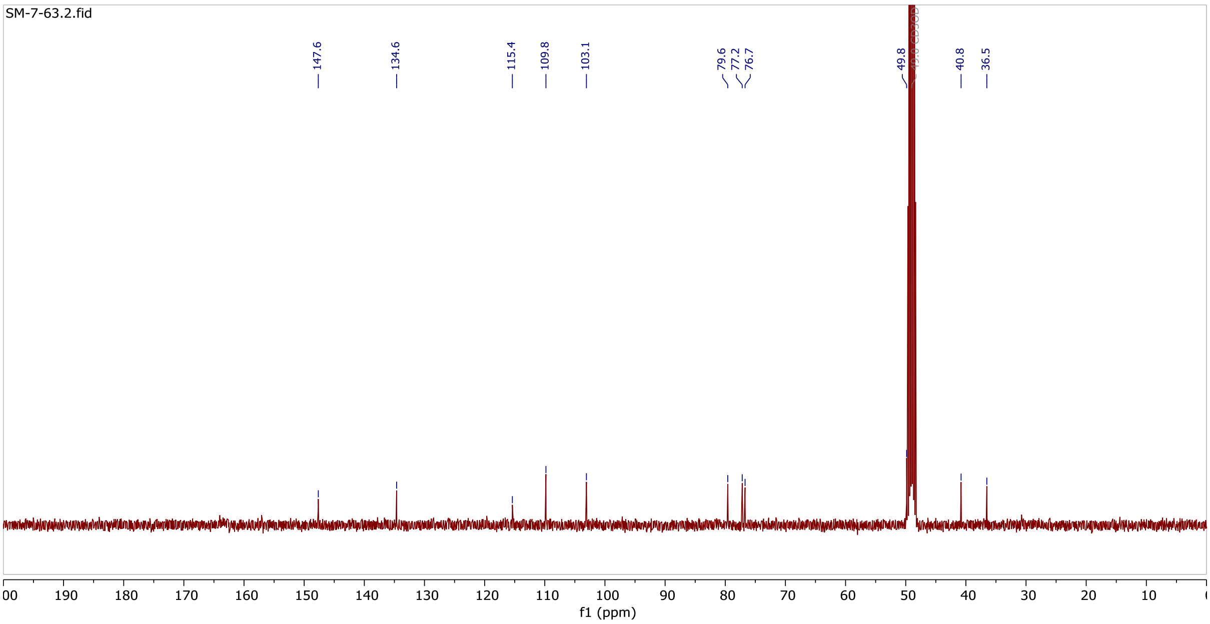

^{13}C NMR spectra of **10**

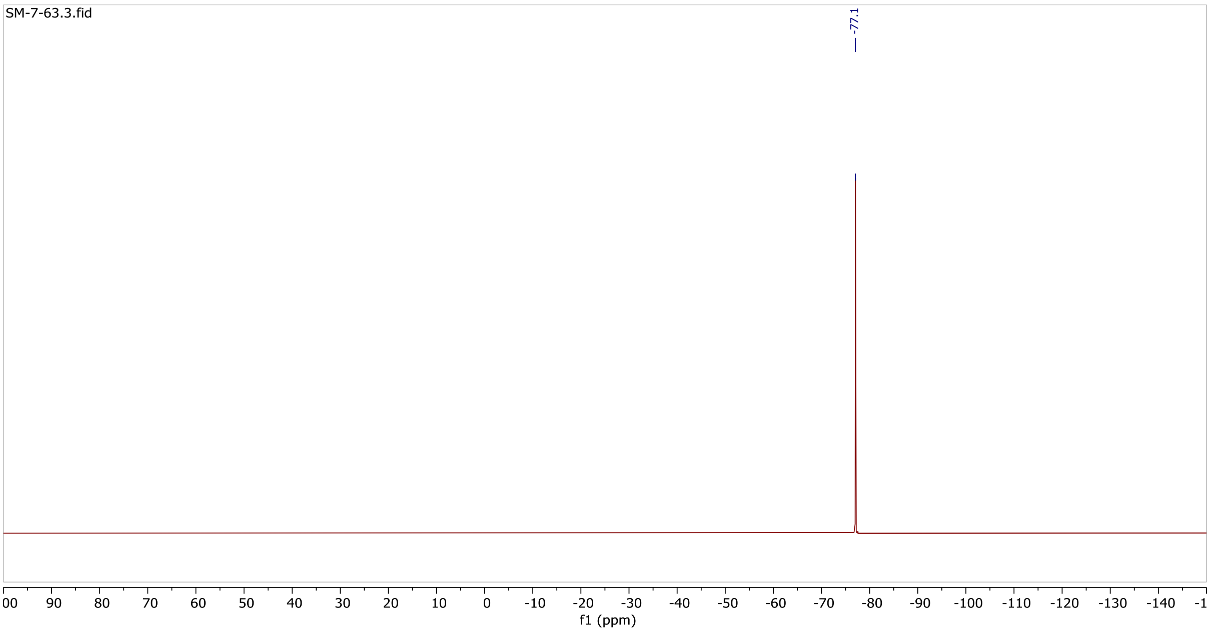

^{19}F NMR spectra of **10**

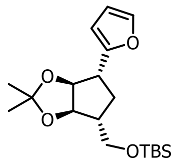

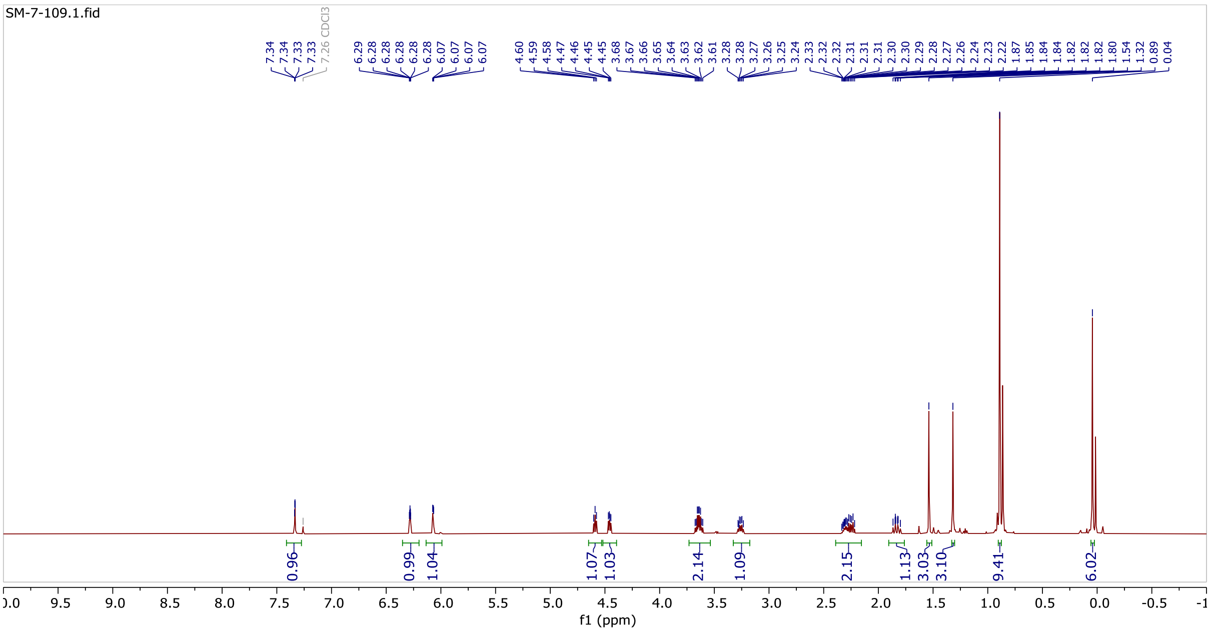

^{1}H NMR spectra of **11**

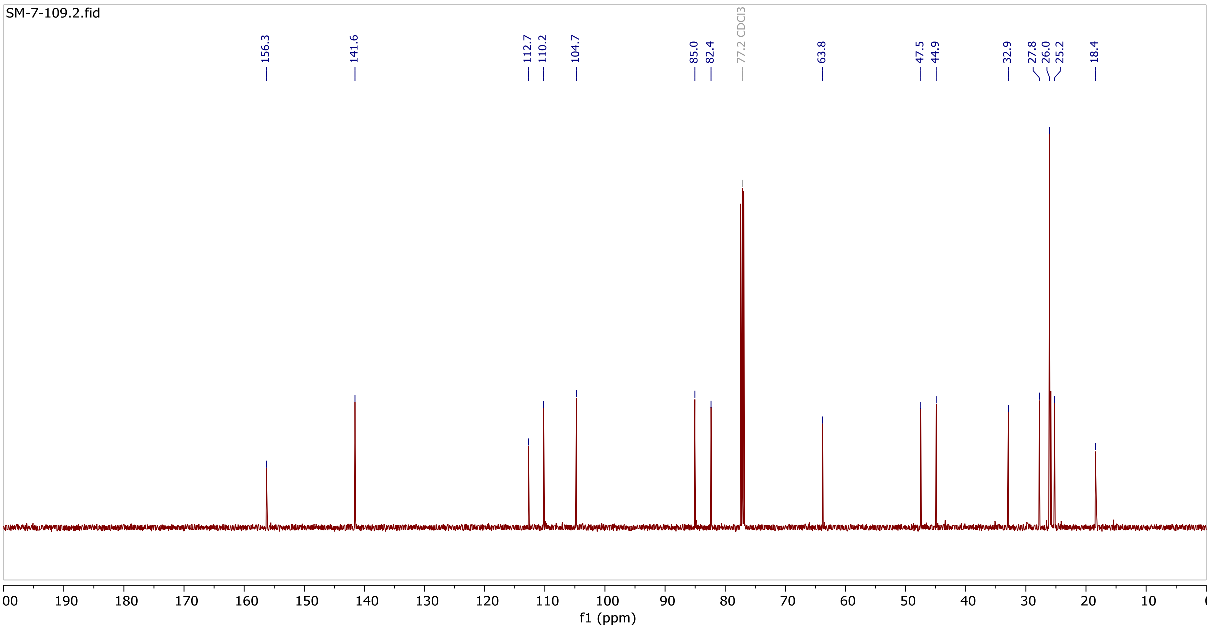

^{13}C NMR spectra of **11**

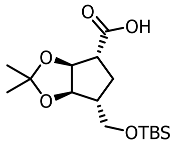

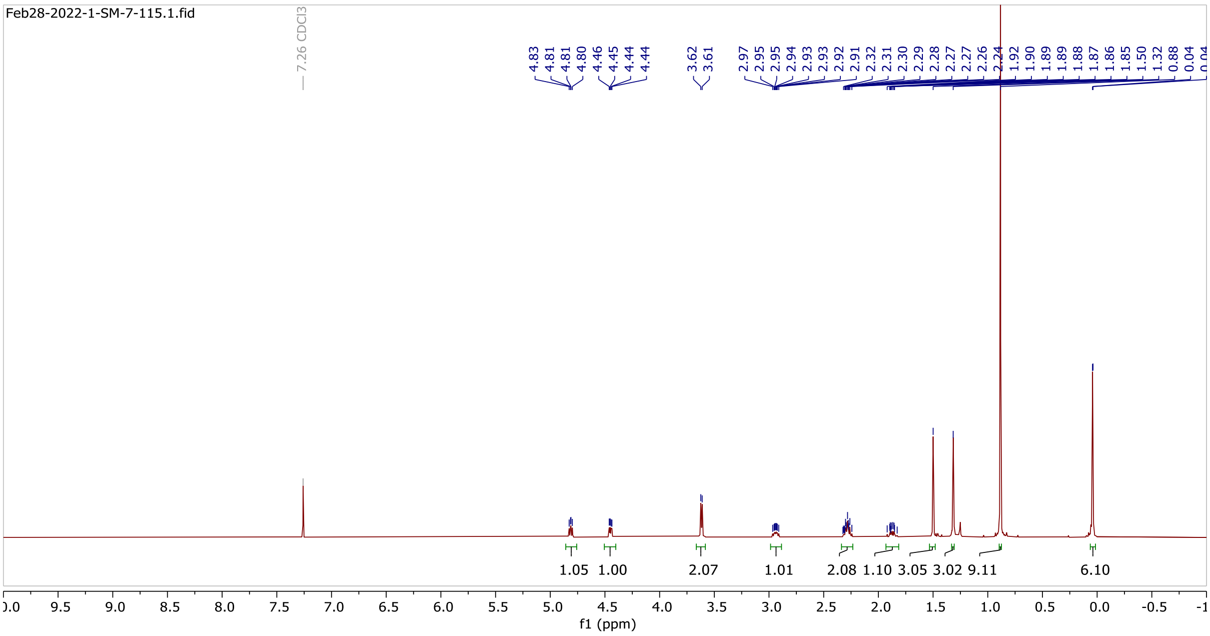

^{1}H NMR spectra of **12**

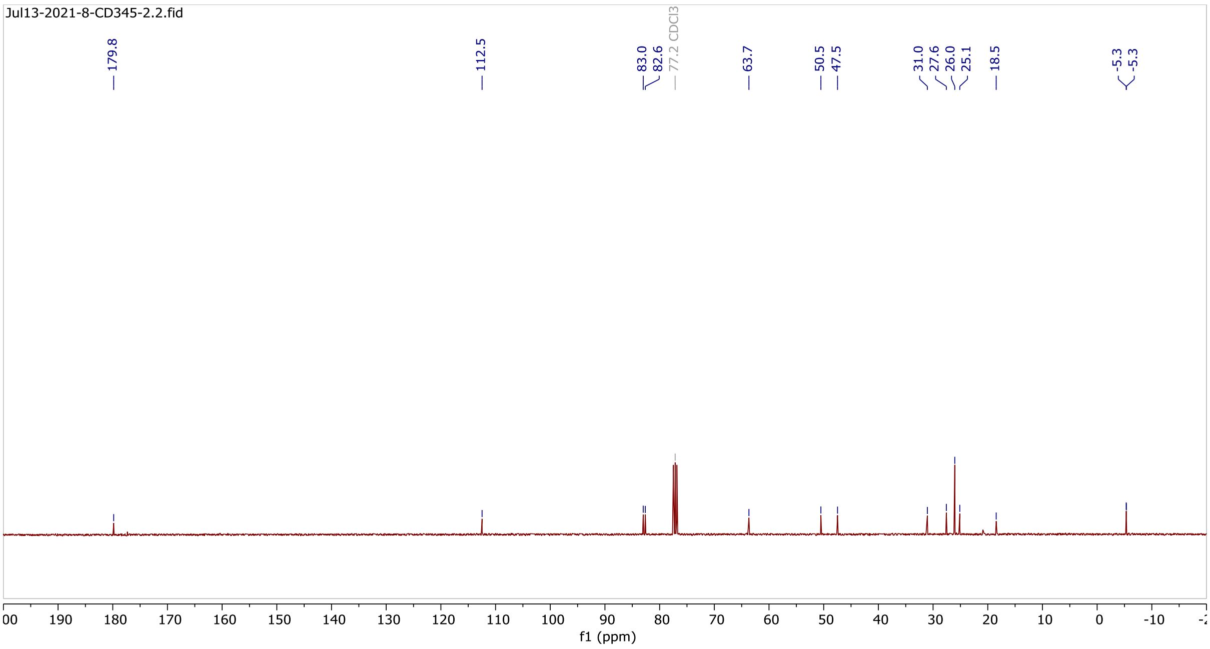

^{13}C NMR spectra of **12**

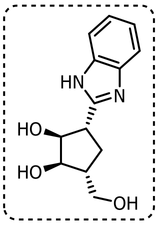

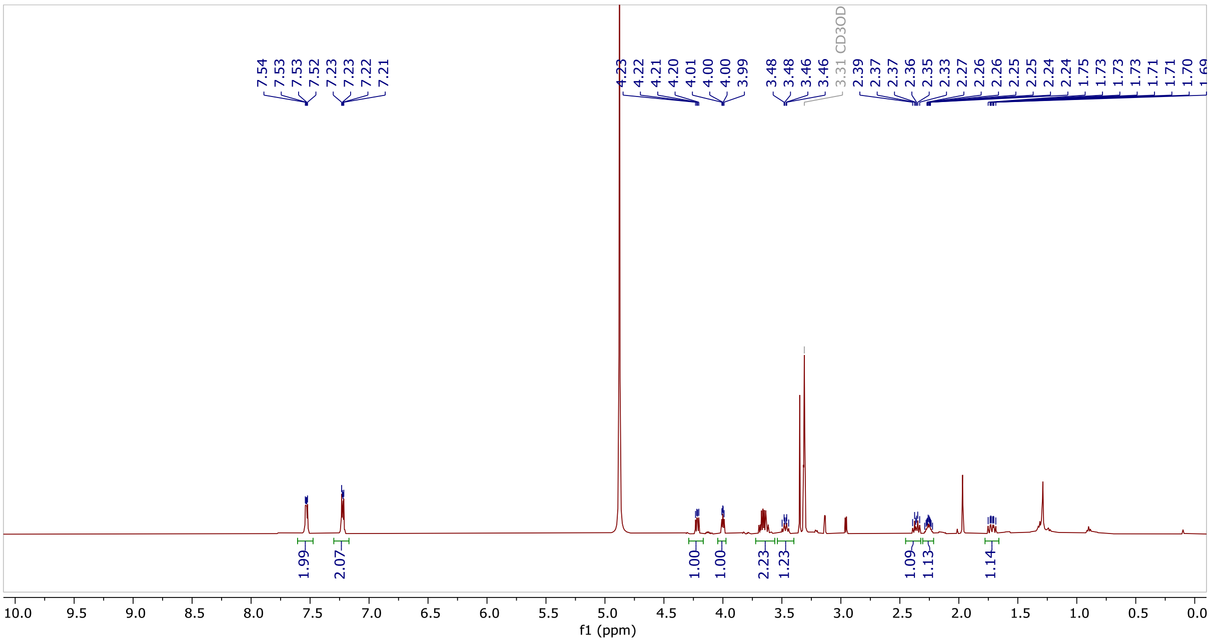

^{1}H NMR spectra of **13**

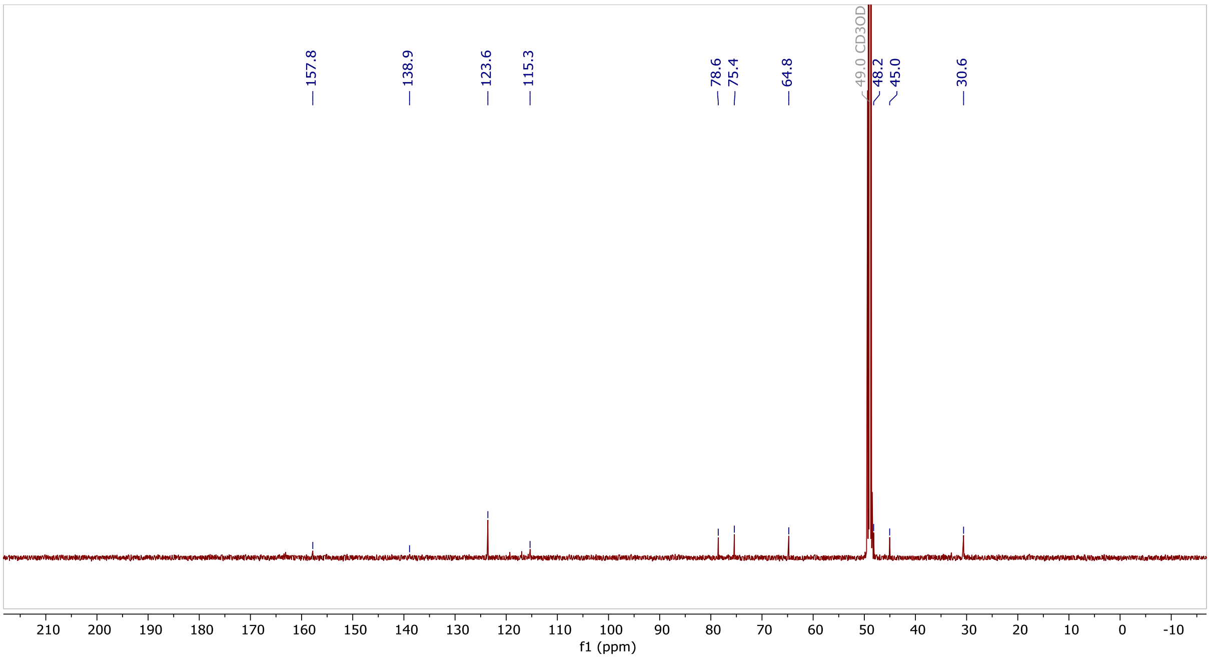

^{13}C NMR spectra of **13**

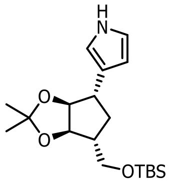

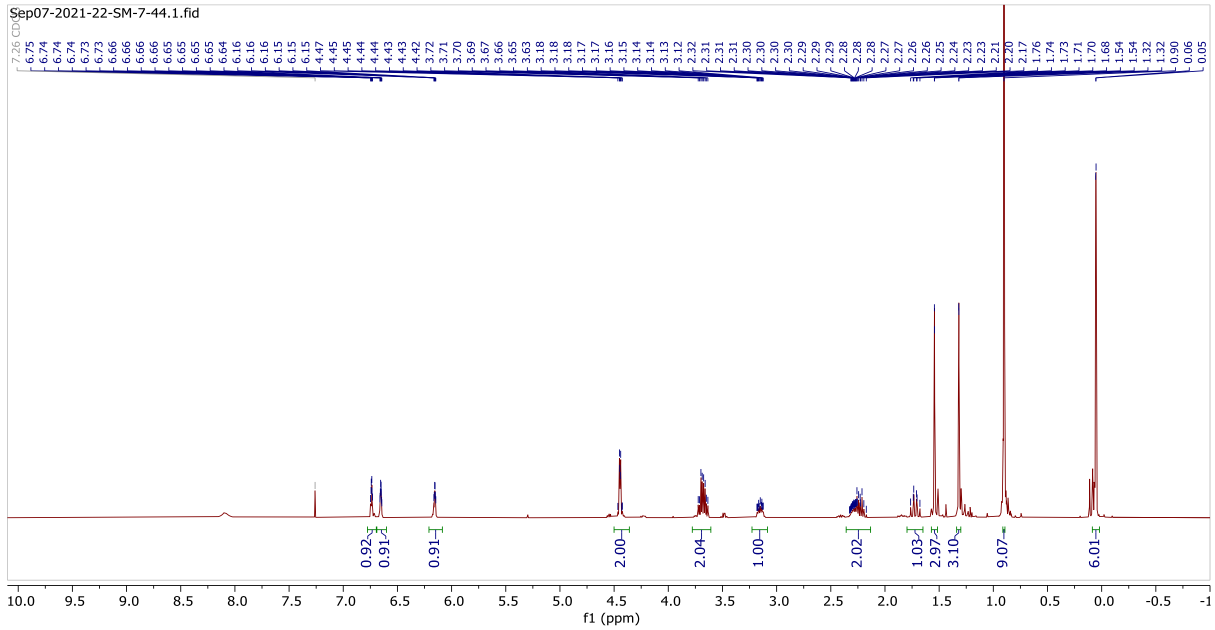

^{1}H NMR spectra of **14**

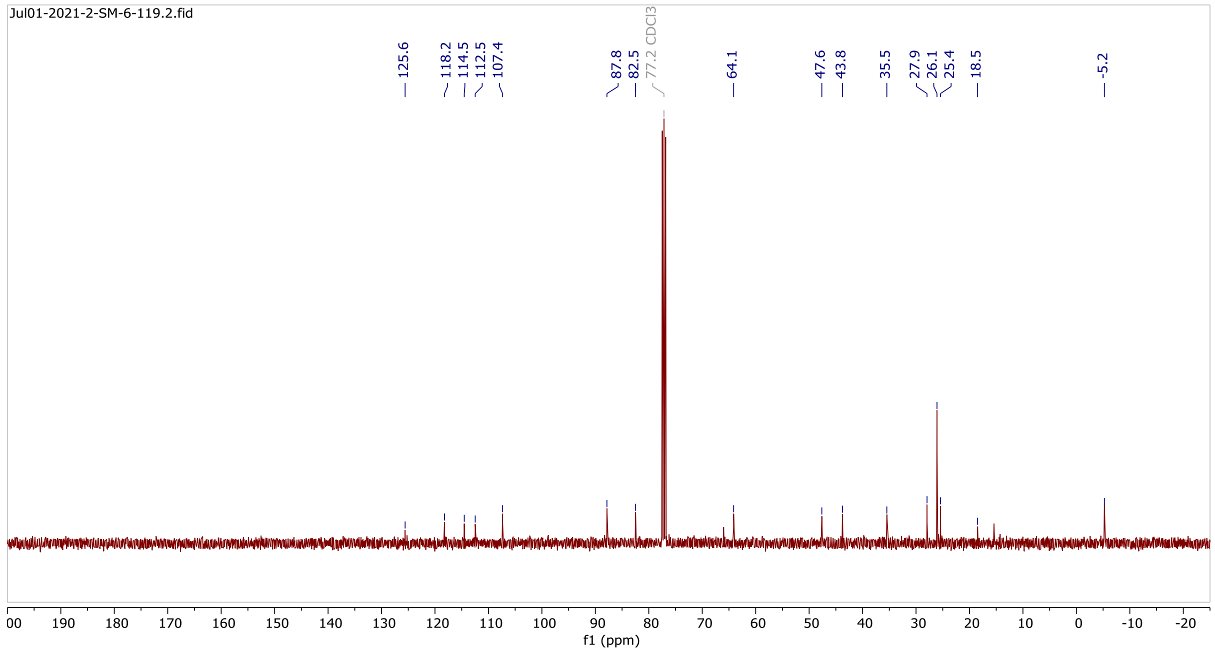

^{13}C NMR spectra of **14**

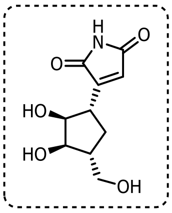

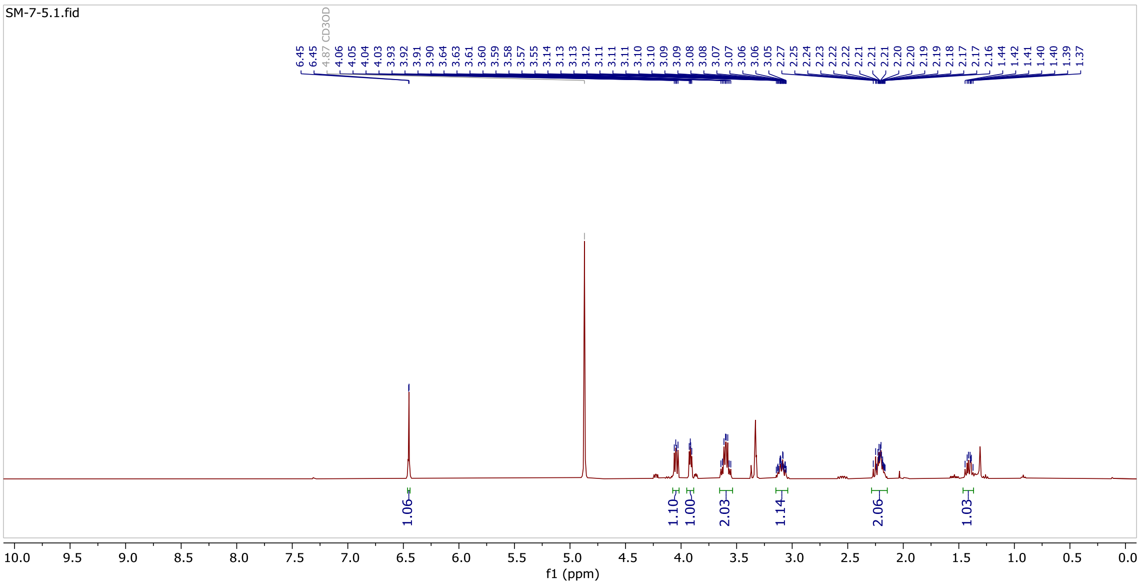

^{1}H NMR spectra of **16**

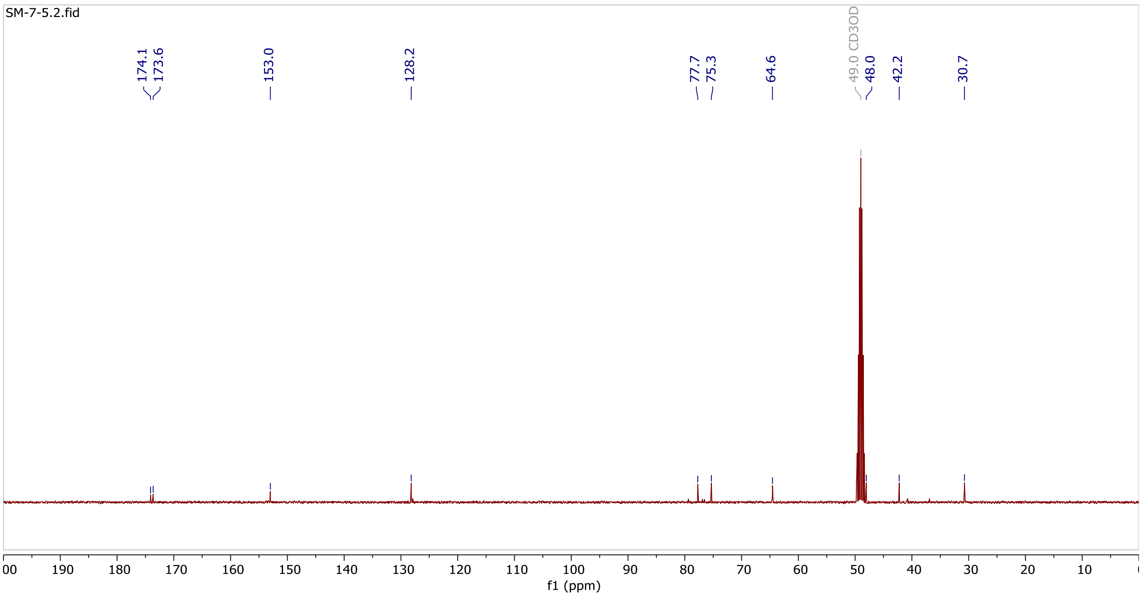

^{13}C NMR spectra of **16**
